# Phospholipid oxidation generates potent anti-inflammatory lipid mediators that mimic structurally related pro-resolving eicosanoids by activating Nrf2

**DOI:** 10.15252/emmm.201404702

**Published:** 2015-03-13

**Authors:** Peter Bretscher, Julian Egger, Abdijapar Shamshiev, Martin Trötzmüller, Harald Köfeler, Erick M Carreira, Manfred Kopf, Stefan Freigang

**Affiliations:** 1 Institute of Molecular Health Sciences, ETH ZurichZurich, Switzerland; 2 Laboratory of Organic Chemistry, Department of Chemistry, ETH ZurichZurich, Switzerland; 3 Core Facility for Mass Spectrometry, Medical University of GrazGraz, Austria

**Keywords:** inflammation, isoprostanes, lung injury, Nrf2, oxidized phospholipids

## Abstract

Exposure of biological membranes to reactive oxygen species creates a complex mixture of distinct oxidized phospholipid (OxPL) species, which contribute to the development of chronic inflammatory diseases and metabolic disorders. While the ability of OxPL to modulate biological processes is increasingly recognized, the nature of the biologically active OxPL species and the molecular mechanisms underlying their signaling remain largely unknown. We have employed a combination of mass spectrometry, synthetic chemistry, and immunobiology approaches to characterize the OxPL generated from the abundant phospholipid 1-palmitoyl-2-arachidonoyl-*sn*-glycero-3-phosphocholine (PAPC) and investigated their bioactivities and signaling pathways *in vitro* and *in vivo*. Our study defines epoxycyclopentenones as potent anti-inflammatory lipid mediators that mimic the signaling of endogenous, pro-resolving prostanoids by activating the transcription factor nuclear factor E2-related factor 2 (Nrf2). Using a library of OxPL variants, we identified a synthetic OxPL derivative, which alleviated endotoxin-induced lung injury and inhibited development of pro-inflammatory T helper (Th) 1 cells. These findings provide a molecular basis for the negative regulation of inflammation by lipid peroxidation products and propose a novel class of highly bioactive compounds for the treatment of inflammatory diseases.

## Introduction

It is now increasingly recognized that oxidized phospholipids (OxPL) do not just represent mere by-products of lipid peroxidation associated with inflammatory conditions or increased oxidative stress, but instead actively modulate cellular signaling processes and contribute to the initiation and amplification of inflammation (Berliner & Watson, 2005; Bochkov *et al*, 2010; Lee *et al*, 2012). Particularly the polyunsaturated fatty acid side chains of membrane phospholipids are highly susceptible to modification by reactive oxygen intermediates, which potentially yields an enormous array of distinct lipid oxidation products with diverse biological effects (Leitinger, 2003; Berliner & Watson, 2005; Bochkov *et al*, 2010; Lee *et al*, 2012). In contrast to the site-specific enzymatic oxidation reactions that convert arachidonic acid into a panel of endogenous inflammatory eicosanoid mediators, such radical-mediated oxidation is non-specific; hence, even the oxidation of a single phospholipid precursor containing arachidonic acid results in a very complex mixture of individual OxPL species (Bochkov *et al*, 2010). The pathophysiological relevance of OxPL was initially discovered in atherosclerosis (Witztum & Steinberg, 1991; Berliner & Watson, 2005), but is now clearly evident for other diseases with a prominent inflammatory component, including acute and chronic microbial infections, lung injury, and neurodegenerative disorders (Bochkov *et al*, 2002; Cruz *et al*, 2008; Imai *et al*, 2008; Weismann *et al*, 2012). The majority of reports suggests strong pro-inflammatory effects of OxPL, which might be explained by the interaction of OxPL with pattern recognition receptors of the innate immune system, such as Toll-like receptors (Imai *et al*, 2008; Seimon *et al*, 2010; Stewart *et al*, 2010), scavenger receptors (Podrez *et al*, 2002; Stewart *et al*, 2010), complement components (Weismann *et al*, 2012), or natural antibodies (Palinski *et al*, 1996; Binder *et al*, 2003). Nevertheless, several conflicting observations indicate that OxPL may also dampen inflammatory responses, and OxPL-mediated inhibition of TLR activation (Bochkov *et al*, 2002) as well as phagocytosis (Knapp *et al*, 2007) has been proposed as underlying mechanism. The discrepancy between potential pro- and anti-inflammatory bioactivities of OxPL is not yet resolved. One possible reason for such contradicting findings may lie in the different experimental conditions used by different groups to generate their respective OxPL preparations, which may lead to different compositions of the resulting bulk OxPL mixtures and could therefore explain the different pro- or anti-inflammatory activities. A solution to this problem would be the use of isolated, synthetic OxPL species to elucidate their mechanism of action and pathophysiological relevance. While the first bioactive OxPL species have been identified and associated with defined biological effects (Watson *et al*, 1997, 1999; Podrez *et al*, 2002; Seimon *et al*, 2010; Zhong *et al*, 2013), our knowledge on the contribution of such lipid species to the *in vivo* regulation of inflammation and on the responsible signaling pathways remains very limited.

Here, we have investigated the spectrum of OxPL species that is generated during oxidative modification of a single, defined phospholipid precursor. We chose 1-palmitoyl-2-arachidonoyl-*sn*-glycero-3-phosphocholine (PAPC) for these experiments because of its abundance in biological membranes and because of the relevance of its oxidation products for inflammatory diseases, including acute lung injury, microbial infection, and atherosclerosis (Watson *et al*, 1997; Bochkov *et al*, 2002; Podrez *et al*, 2002; Cruz *et al*, 2008; Imai *et al*, 2008). Our study characterizes an anti-inflammatory bioactivity of OxPL that can be attributed to a single type of OxPL. We find that this potent anti-inflammatory bioactivity is mediated by the prostanoid-like OxPL component epoxycyclopentenone, which activates the transcription factor nuclear factor E2-related factor 2 (Nrf2) to inhibit pro-inflammatory cytokine and chemokine responses in myeloid cells *in vitro* and *in vivo*. Using a library of epoxycyclopentenone variants, we have defined structural determinants of this bioactivity and developed an epoxycyclopentenone derivative with an unprecedented anti-inflammatory bioactivity. These results not only implicate OxPL/Nrf2 signaling in the negative regulation of inflammation, but also suggest a novel class of lipid mediators as therapeutic agents for the treatment of inflammatory diseases.

## Results

### Oxidized phospholipids are potent inhibitors of the pro-inflammatory response of myeloid cells

**Figure 1:**
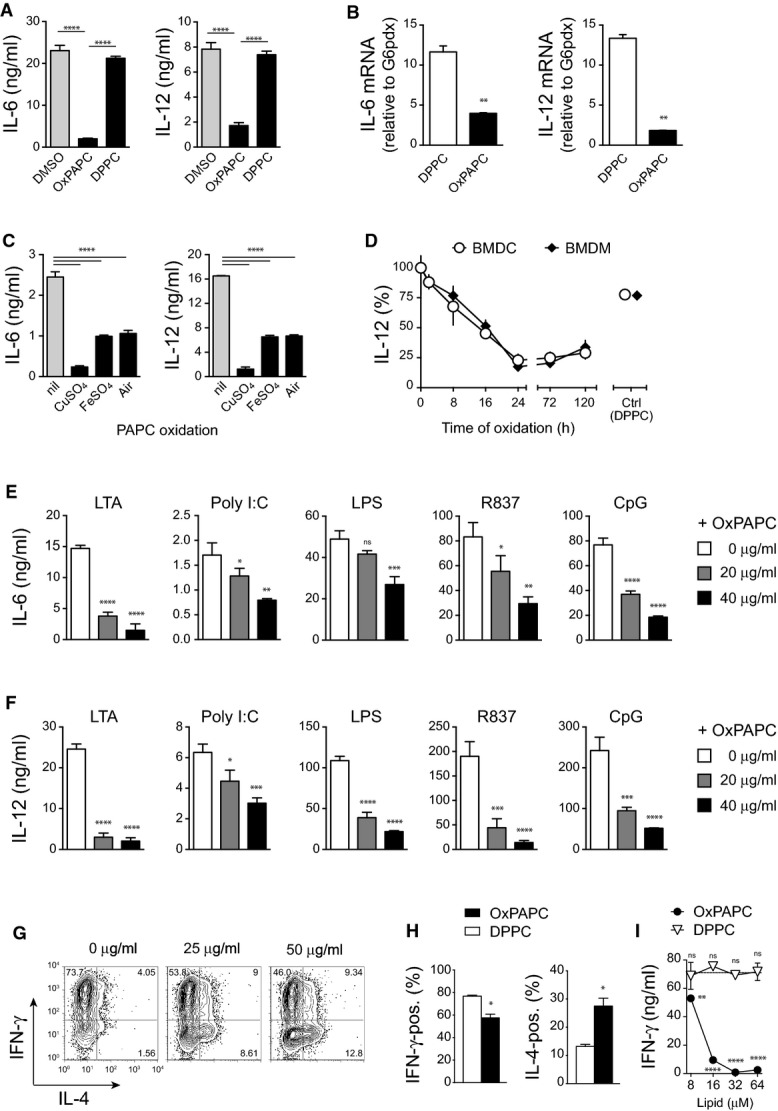
Oxidized phospholipids are potent inhibitors of the pro-inflammatory response of myeloid cells

To examine the effects of OxPL signaling on innate immune responses, synthetic PAPC was either oxidized by metal-catalyzed oxidation or autoxidized by exposure to ambient air, and the bioactivity of the resulting oxidized PAPC (OxPAPC) mixtures was evaluated *in vitro*. Exposure of bone marrow-derived dendritic cells (BMDC) to OxPAPC strongly inhibited their subsequent ability to produce the pro-inflammatory cytokines interleukin (IL)-6 and IL-12 in response to stimulation of the Toll-like receptor (TLR) 7 with imiquimod (Fig1A). This potent anti-inflammatory bioactivity of OxPAPC could be directly attributed to the oxidative modification of PAPC, since treatment with 1,2-di-palmitoyl-*sn*-glycero-3-phosphocholine (DPPC), a phospholipid with no unsaturated acyl chains that is therefore inert to oxidation, did not show such effect (Fig1A). OxPAPC-treated cells produced reduced levels of IL-6 and IL-12 messenger RNA upon TLR stimulation, indicating that OxPL signaling regulated these cytokine responses at the transcriptional level (Fig1B). While copper-catalyzed PAPC oxidation appeared to be the most rapid and potent method to generate anti-inflammatory OxPL species, also iron-oxidized and autoxidized OxPAPC preparations comparably inhibited the inflammatory response of myeloid cells (Fig1C). We concluded from this result that modification of PAPC by reactive oxygen species generates anti-inflammatory OxPL irrespective of the method used, albeit with different efficacy and kinetics. Weak anti-inflammatory bioactivity was already detectable after 2 h of copper-catalyzed PAPC oxidation (Fig1D). The bioactivity of the OxPAPC preparation progressively increased to reach its maximum at 24 h of oxidation and remained constant at this level during further oxidation for at least 5 days (Fig1D). This observation suggested that generation of the anti-inflammatory OxPL species required a certain degree of oxidation to occur. Still, once formed, the bioactive OxPL species appeared to be relatively stable, at least under the conditions used for PAPC oxidation *in vitro*.

A, B BMDCs were treated with OxPAPC or DPPC (40 μg/ml) for 60 min followed by R837 stimulation (5 μg/ml). (A) Supernatants were harvested after 18 h, and concentrations of IL-6 and IL-12 were quantified by ELISA. One-way ANOVA adjusted by Dunnett's multiple comparisons test. Mean ± SD of triplicate determinations from > 3 independent experiments are shown. (B) mRNA was harvested after 2 h, and expression of IL-6 and IL-12 was measured by real-time PCR and normalized to G6pdx. Unpaired two-tailed *t*-test. Data (mean ± SD) are representative of three independent experiments.

C Bioactive OxPAPC mixtures were obtained by various oxidation protocols from highly pure PAPC. Periods of oxidation were 24 h for CuSO_4_ (10 μM), 48 h for FeSO_4_ (10 μM), and 72 h for air. BMDCs were treated for 60 min with lipids prior stimulation with R837 (5 μg/ml) for 18 h. Cytokine concentrations in supernatants were quantified by ELISA. One-way ANOVA adjusted by Dunnett's multiple comparisons test. Data represent mean ± SEM of triplicate determinations.

D PAPC (40 μg/ml) oxidized with CuSO_4_ (10 μM) for the indicated times was used for treatment of BMDCs prior to R837 (5 μg/ml) stimulation and measurement of IL-12 secretion. Mean ± SEM of triplicate determinations from three different oxidation series are shown.

E, F Treatment of BMDCs with the indicated concentrations of CuSO_4_-oxidized PAPC suppressed IL-6 (E) and IL-12 (F) secretion triggered by a variety of different TLR agonists. After OxPAPC treatment for 60 min, BMDCs were stimulated for 18 h with LTA (500 ng/ml), Poly I:C (50 μg/ml), LPS (10 ng/ml), R837 (5 μg/ml), and CpG (100 nM). Cytokine concentrations in the supernatant were quantified by ELISA. Data are shown as mean ± SD of triplicate determinations from three independent experiments and were analyzed by one-way ANOVA adjusted by Dunnett's multiple comparisons test.

G–I Splenic dendritic cells were treated with OxPAPC or DPPC (40 μg/ml) before co-culturing with naïve transgenic SMARTA CD4 T cells in the presence of the specific peptide gp61. (G) After 4 days of cell culture, T-cell polarization was assessed by intracellular staining for the cytokines IL-4 (Th2) and IFN-γ (Th1). (H) Bar graphs represent the frequencies of IL-4- and IFN-γ-producing T cells after 4 days of co-culture with OxPAPC-treated and DPPC-treated splenic dendritic cells. Bars represent mean ± SD of duplicate experiments. *P ≤* *0.05 by unpaired two-tailed *t*-test. (I) IFN-γ production in supernatants of SMARTA CD4 T cells (stimulated with 1,000 nM gp61) and co-cultured with OxPAPC- and DPPC-treated splenic dendritic cells for 4 days. One-way ANOVA adjusted by Dunnett's multiple comparisons test. Bars represent mean ± SD. Data information: *P <* *0.05; **P <* *0.01; ***P <* *0.001; ****P <* *0.0001; ns, not significant.

The ability of OxPL to interfere with innate inflammation was not limited to cytokine responses induced via TLR 7 (Fig1A), since OxPAPC also strongly suppressed the IL-6 and IL-12 secretion elicited by microbial agonists of the TLRs 2, 3, 4, and 9 (Fig1E and F). A contribution of toxicity to these effects could be ruled out by performing live staining and by verifying cellular metabolic activity at the end of experiments (Supplementary Figs S1 and S2). Moreover, the fact that OxPAPC-mediated suppression similarly affected the inflammatory response to several TLR ligands also rendered the inactivation of a particular TLR ligand by OxPAPC (Bochkov *et al*, 2002; Blüml *et al*, 2005), an unlikely explanation for the strong effects observed in our experiments. Thus, the OxPL species formed during the oxidative modification of PAPC potently inhibited the innate inflammatory response of myeloid cells.

Dendritic cell (DC)-produced IL-12 is critical for directing the differentiation of naïve CD4^+^ T cells toward the T helper (Th) 1 cell subset and thus essentially contributes to the shaping of adaptive immune responses. We therefore next assessed whether the OxPL-inhibited IL-12 production would impact the ability of DCs to license Th1 polarization of naive T cells using an *in vitro* co-culture system in which naïve T-cell receptor transgenic CD4^+^ T cells are activated by splenic DCs in the presence of their cognate peptide. Indeed, prior exposure of DCs to OxPAPC inhibited their subsequent ability to drive the generation of interferon-gamma (IFN-γ)-producing Th1 T cells and instead promoted the generation of IL-4-producing Th2 T cells, whereas DPPC treatment showed no comparable effect (Fig1G and H). OxPAPC treatment not only reduced the frequency of T cells producing IFN-γ, but also diminished the absolute amount of T-cell-secreted IFN-γ protein (Fig1I). Altogether, these findings demonstrated a strong anti-inflammatory bioactivity of OxPAPC and suggested that OxPL may influence both innate and adaptive immune responses *in vivo*.

### *In vitro* generated OxPAPC preparations represent complex mixtures of OxPL species with distinct bioactivities

**Figure 2:**
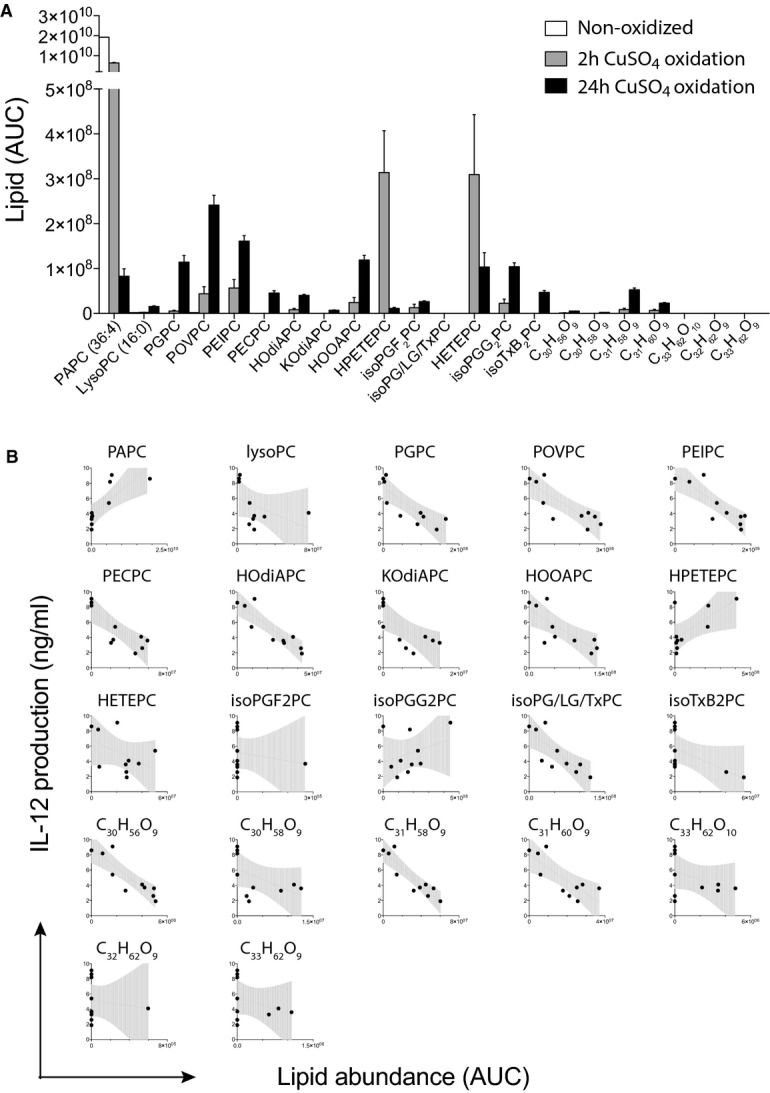
*In vitro* generated OxPAPC preparations represent complex mixtures of OxPL species with distinct bioactivities

Both pro- and anti-inflammatory activities of OxPAPC have been reported (Berliner & Watson, 2005; Bochkov *et al*, 2010), whereas our results primarily revealed an anti-inflammatory effect of various OxPAPC preparations. We reasoned that the overall bioactivity of a given OxPAPC preparation likely results from the combined properties of its components and is thus determined by the relative concentrations of individual pro- and/or anti-inflammatory OxPL species present within the respective OxPAPC mixture. For example, while OxPAPC generated using copper-catalyzed oxidation for 2 h predominantly contained intermediate oxidation products of PAPC, these OxPL species were almost absent after 24 h of oxidation when the strongest bioactivity was detected (Fig2A). Instead, OxPAPC oxidized with copper sulfate for 24 h contained substantial amounts of more complex oxidation products, such as 1-palmitoyl-2-(5-oxovaleroyl)-*sn*-glycero-3-phosphocholine (POVPC), 1-palmitoyl-2-glutaryl-*sn*-glycero-3-phosphocholine (PGPC), 1-palmitoyl-2-(5,6-epoxyisoprostane E2)-*sn*-glycero-3-phosphocholine (PEIPC), and 1-palmitoyl-2-(5,6-epoxyisoprostane A2)-*sn*-glycero-3-phosphocholine (PECPC) (Fig2A). To identify the anti-inflammatory OxPL species, we therefore deliberately varied both the oxidation times and oxidative agents in order to generate a panel of different OxPAPC preparations that exhibited titrated degrees of anti-inflammatory bioactivity. We then examined the OxPL composition of the differentially oxidized OxPAPC preparations by mass spectrometry analysis and investigated whether the overall bioactivity of these complex OxPL mixtures correlated with the abundance of any of their OxPL components (Fig2B). Our results suggested previously known OxPL, such as PGPC, POVPC, PEIPC, and 1-palmitoyl-2-(5-keto-6-octene-dioyl)-*sn*-glycero-3-phosphocholine (KOdiAPC) as likely candidates, but also less well-characterized OxPL species including PECPC, or even OxPL of yet undetermined structure. Thus, the correlative analysis of bulk OxPL mixtures indicated a limited number of candidate lipids as potential anti-inflammatory OxPAPC components (Fig2B).

Mass spectrometric quantification of a variety of OxPL species obtained by CuSO_4_-catalyzed oxidation of PAPC for 2 and 24 h. Mean ± SEM of duplicate determinations are shown.

Correlation between the abundance of individual OxPL species detected in mixtures of differentially oxidized OxPL preparations and the capacity of the respective overall OxPL mixtures to suppress the IL-12 secretion of thioglycollate-elicited macrophages. Dots represent data of individual OxPAPC preparations.

### An OxPL containing a fatty acid epoxycyclopentenone mediates the anti-inflammatory bioactivity of OxPAPC

**Figure 3:**
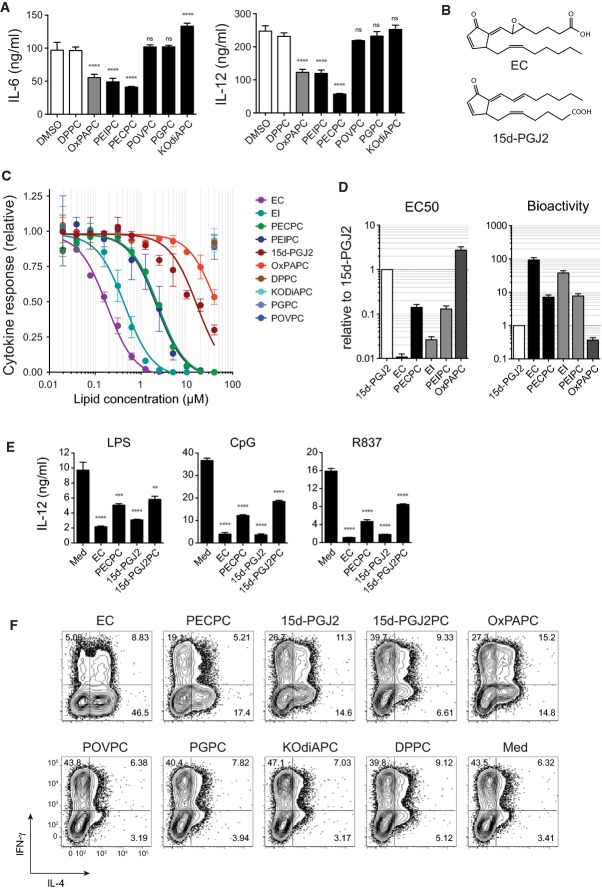
An OxPL containing a fatty acid epoxycyclopentenone mediates the anti-inflammatory bioactivity of OxPAPC

To unambiguously identify the relevant OxPL species, we next tested the bioactivity of each of these candidate lipids in isolated form using synthetic compounds. We focused our analysis on OxPL that was either commercially available or synthesized by us according to recently established routes (Egger *et al*, 2013). While PEIPC showed an anti-inflammatory bioactivity similar to that of OxPAPC, the less characterized PECPC exhibited slightly higher efficacy (Fig3A). In contrast, neither of the truncated OxPL associated with cardiovascular inflammation (Watson *et al*, 1997; Podrez *et al*, 2002), for example, POVPC, PGPC, or KOdiAPC, inhibited the TLR-induced inflammatory response in our experiments (Fig3A). The close structural homology of the fatty acid epoxycyclopentenone detected at the sn2 position of PECPC to the endogenous, pro-resolving prostaglandin 15-deoxy-Δ12,14-Prostaglandin J2 (15d-PGJ2) prompted us to further explore the functional relationship between PECPC and 15d-PGJ2. Given that 15d-PGJ2 is physiologically generated and active as the isolated prostanoid *in vivo*, we also examined the corresponding fatty acid epoxycyclopentenone (EC) and fatty acid epoxyisoprostane (EI) in our bioassays (Fig3C and D). While the inhibition of cytokine production provided by PECPC and PEIPC exceeded that of 15d-PGJ2 by an order of magnitude, the prostanoid-like EI and EC exhibited a 40-fold and 100-fold stronger bioactivity than 15d-PGJ2, respectively (Fig1C and D). Thus, EC and EI appeared to represent the active components of PECPC and PEIPC, as their efficacy greatly increased when provided to cells in isolated form, that is, not esterified to a lysophospholipid. Accordingly, also the bioactivity of the synthetic phospholipid 1-palmitoyl-2-(15-deoxy-Δ12,14-Prostaglandin J2)-*sn*-glycero-3-phosphocholine (15d-PGJ2-PC), which contains 15d-PGJ2 esterified at the sn2 position, showed much weaker effects than 15d-PGJ2 itself (Fig3E). Together, these findings identified PECPC and PEIPC as the OxPL species mediating the anti-inflammatory bioactivity of bulk OxPAPC preparations and suggested that they mimic the physiological activity of the endogenous, pro-resolving lipid mediator 15d-PGJ2. This notion was further corroborated by the ability of EC and 15d-PGJ2 to modulate Th-cell polarization in our *in vitro* co-culture system (Fig3F). As could have been anticipated from above observations, EC and 15d-PGJ2 as well as their respective OxPL, PECPC, and 15d-PGJ2PC efficiently limited Th1-cell polarization, whereas POVPC, PGPC, and KOdiAPC had no such effect (Fig3F) consistent with the inability to inhibit IL-12 production (Fig3A and C). Collectively, these data identified EC as the most potent anti-inflammatory OxPAPC component and implicated the molecular pathways that are physiologically targeted by 15d-PGJ2 as potential mechanism for this activity.

Selected candidate lipids were tested for their inhibitory activity on R837-induced (5 μg/ml; 18 h) cytokine secretion in BMDCs. Concentrations of indicated lipids: PECPC (10 μM), PEIPC (10 μM), OxPAPC (40 μg/ml), DPPC (40 μg/ml), POVPC (40 μM), PGPC (40 μM), and KOdiAPC (40 μM). Representative data (mean ± SD of triplicate determinations) from one of three independent experiments are shown. ****P < 0.0001; ns, not significant; as determined by one-way ANOVA adjusted by Sidak's multiple comparisons test.

Structures of the cyclopentenone-containing arachidonic acid-derived lipids EC and 15d-PGJ2.

Dose–response curves of IL-12 secretion in BMDCs pulsed for 60 min with the indicated lipids, followed by stimulation with R837 (5 μg/ml) for 18 h. Toxic concentrations of lipids were excluded from analysis. Mean ± SEM of triplicate determinations are shown.

EC50 values of anti-inflammatory lipid products normalized to the capacity of 15d-PGJ2 to suppress IL-12 production in BMDCs (left panel) and their respective bioactivities (right panel) depicted as the fold increase relative to 15d-PGJ2. Mean ± SEM of triplicate determinations are shown.

IL-12 production of BMDC stimulated via TLR 4 (LPS; 100 ng/ml), TLR 9 (CpG; 100 nM), and TLR 7 (R837; 5 μg/ml) after pretreatment with the indicated free and esterified versions of EC and 15d-PGJ2. Lipids were used at 1 μM (EC), 10 μM (PECPC), or 20 μM (15d-PGJ2 and 15d-PGJ2PC). Data (mean ± SEM) are representative of 3 independent experiments. **P ≤* *0.01; ***P ≤* *0.001; ****P ≤* *0.0001; determined by one-way ANOVA adjusted by Dunnett's multiple comparisons test.

Analysis of the capacity of various OxPL-derived species to license splenic dendritic cells to polarize naïve CD4 T cells toward the Th2 subset. Concentrations of indicated lipids: EC (1 μM), PECPC (10 μM), 15d-PGJ2 (20 μM), OxPAPC (40 μg/ml), DPPC (40 μg/ml), POVPC (40 μM), PGPC (40 μM), KOdiAPC (40 μM).

### Epoxycyclopentenone lipids inhibit the inflammatory response of myeloid cells via nuclear factor E2-related factor 2 (Nrf2) signaling

**Figure 4:**
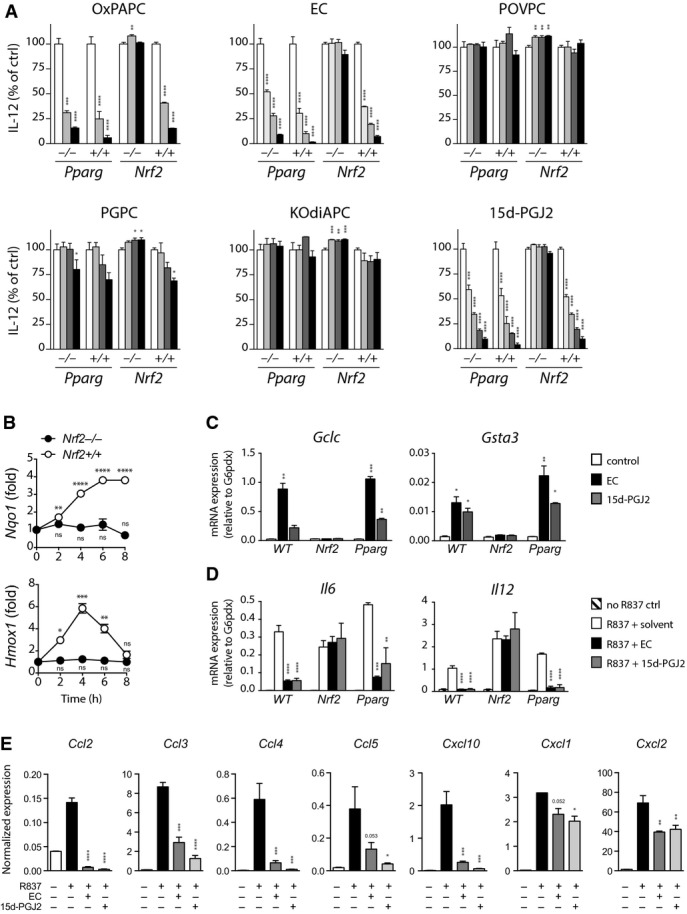
Epoxycyclopentenone lipids inhibit the inflammatory response of myeloid cells via Nrf2 signaling

Our previous results described a potent anti-inflammatory effect of OxPAPC and identified EC as principal mediator of this bioactivity. In addition, the close structural and functional similarity between EC and 15d-PGJ2 suggested both molecules induced these effects by activating similar signaling pathways. 15d-PGJ2 has been reported to interact with the nuclear hormone receptor peroxisome proliferator-activated receptor-gamma (PPAR-γ) as well as with the oxidative stress-responsive transcription factor Nrf2. Since both molecules have been implicated in the transcriptional regulation of inflammation, we next examined their contribution by assessing the anti-inflammatory activity of a series of synthetic OxPL in the respective gene-deficient BMDC (Fig4A). Whereas removal of PPAR-γ did not alter the OxPL-mediated inhibition of IL-12 production, the bioactivity of OxPAPC, EC, and 15d-PGJ2 was abrogated in the absence of Nrf2, demonstrating that EC and related OxPL signal through Nrf2 to mediate their anti-inflammatory effects. Indeed, stimulation with EC rapidly induced an Nrf2-dependent transcription of the prototypic Nrf2 targets Hmox1 and Nqo1 (Fig4B). Similar to EC, also 15d-PGJ2 triggered the expression of the Nrf2-regulated genes Gclc and Gsta3 in wild-type and PPAR-γ-deficient cells, but not in cells lacking Nrf2 (Fig4C), implying that both lipids mediated their anti-inflammatory activity via Nrf2 rather than PPAR-γ. Indeed, EC and 15d-PGJ2 inhibited the transcription of IL-6 and IL-12 genes in TLR-stimulated wild-type and PPAR-γ-deficient macrophages with comparable efficacy, while Nrf2-deficient cells remained unaffected by this treatment. Together, these results demonstrated that EC and 15d-PGJ2 inhibited pro-inflammatory cytokine responses through a shared mechanism and implicated OxPL/Nrf2 signaling in the regulation of inflammation. The anti-inflammatory effect of the Nrf2-activating prostanoids was not limited to IL-6 and IL-12 production, as both lipids virtually abolished the expression of several pro-inflammatory chemokines including CCL2, CCL3, CCL4, CCL5, and CXCL10 in TLR-stimulated BMDC (Fig4E). Notably, we observed that compared to wild-type controls, Nrf2-deficient cells generally exhibited enhanced TLR-induced cytokine and chemokine responses (Supplementary Fig S3), suggesting that Nrf2 signaling might act as a negative regulator that sets the inflammatory tone in response to endogenously generated OxPL or related prostanoid lipid mediators.

A IL-12 production of BMDC from wild-type, Nrf2^−/−^, Pparg^−/−^, and Pparg litter mate control mice normalized to medium control (open bars). Cells were treated with the indicated lipids (filled bars) for 60 min prior TLR 7 ligation with R837 (5 μg/ml) for 18 h. Lipids were used at starting concentrations of 40 μM (POVPC, PGPC, and KOdiAPC), 40 μg/ml (OxPAPC), 20 μM (15d-PGJ2), and 1.25 μM (EC), depicted as black bars, and 2-fold serial dilutions thereof (gray bars). Data represent mean ± SEM of triplicates from one of three independent experiments.

B Expression of Nrf2 target genes Hmox1 and Nqo1 in wild-type and Nrf2^−/−^ BMDM stimulated with EC (2 μM) for 60 min. Gene expression levels are presented relative to that of untreated cells after normalization to G6pdx. Data (mean ± SEM) are representative of two independent experiments.

C, D mRNA expression levels of the Nrf2 targets Gclc and Gsta3 (C) and of the pro-inflammatory cytokines IL-6 and IL-12 (D) in wild-type, PPAR-γ-deficient, and Nrf2-deficient BMDM after treatment with EC or 15d-PGJ2 for 60 min followed by LPS treatment for 3 h. Expression levels are normalized to G6pdx. Data represent mean ± SEM of triplicate cultures from one of two independent experiments.

E mRNA expression of the indicated chemokines as determined by qPCR. Wild-type BMDCs were treated with EC (1 μM) or 15d-PGJ2 (20 μM) for 60 min followed by R837 stimulation (5 μg/ml) for 3 h. Expression levels are shown normalized to G6pdx. Data (mean ± SEM, *n *=* *2) are representative of three independent experiments. Data information: *P <* *0.05; **P <* *0.01; ***P <* *0.001; ****P <* *0.0001; ns, not significant; as determined by one-way ANOVA adjusted by Dunnett's multiple comparisons test.

### EC mitigates sepsis-associated inflammation *in vivo*

**Figure 5:**
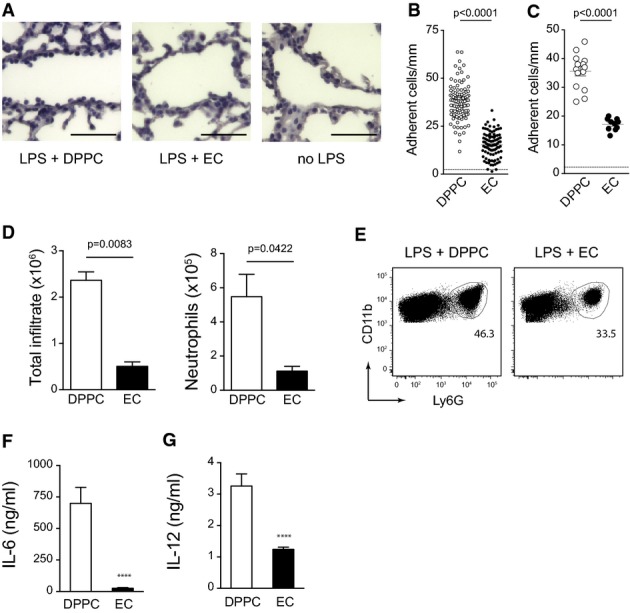
EC mitigates sepsis-associated inflammation *in vivo*

Our study so far established EC as a potent anti-inflammatory OxPL component that signals through Nrf2 to inhibit pro-inflammatory cytokine and chemokine responses of myeloid cells. We next sought to test the efficacy of EC to inhibit inflammatory responses in a model of sepsis-associated lung inflammation *in vivo*. For this purpose, mice were intravenously administered with EC or the control phospholipid DPPC 2 h before receiving an intra-peritoneal challenge with a lethal dose of LPS in the presence of D-galactosamine (D-Gal). While systemic LPS/D-Gal application resulted in the massive adhesion of blood mononuclear cells to the microvascular lung endothelium in DPPC-treated control mice, no comparable adhesion was observed after EC pretreatment (Fig5A), which efficiently induced Nrf2 signaling *in vivo* (Supplementary Fig S4). Instead, the extent of cellular adhesion observed in the lung vasculature of EC-treated animals rather resembled that of naïve controls not treated with LPS (Fig5A). This potent effect of EC was illustrated by a quantitative morphometric analysis confirming that EC pretreatment significantly reduced the number of adherent cells per defined vessel length (Fig5B and C). Prior i.t. administration of EC also efficiently interfered with leukocyte migration into the lung upon i.p. LPS challenge. In particular, EC-treated animals exhibited significantly smaller total infiltrates and reduced absolute neutrophil numbers in their lungs (Fig5D and E) as compared to DPPC-treated controls. Complementing our *in vitro* findings, EC also strongly decreased the LPS-induced secretion of the pro-inflammatory cytokines IL-6 (Fig5F) and IL-12 (Fig5G) *in vivo*. Thus, EC efficiently inhibited acute inflammatory responses *in vivo* and protected mice from sepsis-associated vascular and pulmonary inflammation.

A C57BL/6 mice were treated (i.v.) with 500 μg EC or DPPC control 2 h prior to i.p injection of 150 ng/g LPS together with 800 μg/g D-galactosamine. 4 h after LPS application, lungs were perfused with PBS and embedded in paraffin. Tissue sections were hematoxylin-stained to visualize adherent cells. Bars represent 100 ?m.

B, C Leukocyte adhesion to lung microvascular endothelium as determined by morphometric image analysis of lung tissue sections is presented for individual vessels in (B) and as averages of single mice in (C). Pooled data of two independent experiments are shown (*n *=* *10 for EC, *n *=* *14 for DPPC). Unpaired two-tailed *t*-test.

D, E C57BL/6 mice were treated with EC or DPPC by intra-tracheal instillation at 18 h (50 μg) and 1.5 h (100 μg) prior to i.p. injection of 150 ng/g LPS and 800 μg/g D-galactosamine. Bar graphs represent absolute numbers of total infiltrating cells and of neutrophils (D). Unpaired two-tailed *t*-test. Data represent mean ± SEM from one of two independent experiments with at least 6 mice per group. (E) Dot plots depict exemplary gating of lung neutrophils on pregated CD45^+^ CD11c^−^ SiglecF^−^ BAL cells of EC-/LPS-treated and DPPC-/LPS-treated mice.

F, G The concentrations of IL-6 (F) and IL-12 (G) in the BAL of mice treated as in (D) were quantified by ELISA. Data (mean ± SEM) are representative of two independent experiments with 3 mice per group. ****P < 0.0001; unpaired two-tailed *t*-test.

### Structure–function studies identify critical molecular determinants of the anti-inflammatory bioactivity

**Figure 6:**
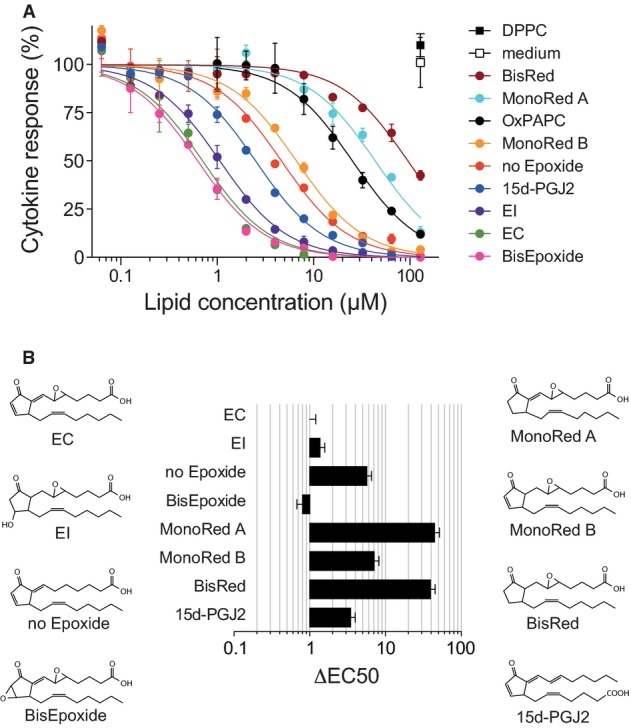
Structure–function studies identify critical molecular determinants of the anti-inflammatory bioactivity

These promising *in vivo* observations encouraged us to further investigate the structure–activity relationship of EC in order to elucidate key structural determinants mediating its potent bioactivity. We hypothesized a potential involvement of the epoxide group as well as the endocyclic and exocyclic enones and therefore evaluated the bioactivity of synthetic EC variants that selectively lacked these electrophilic sites (Fig6A and B). Our results revealed the cyclopentenone double bond as main driver of the overall bioactivity, since its removal in variant ‘MonoRed A’ completely abolished the anti-inflammatory property of EC. This notion was further supported by the fact that introduction of another electrophilic group, an epoxide, at this position into ‘MonoRed A’, which led to the variant ‘Bisepoxide’, restored its bioactivity. In addition, also the epoxide group and the extra-cyclic double bond in α,β position to the carbonyl group appeared to partially contribute to the overall bioactivity, as was illustrated by the reduced efficacy of the respective variants ‘No Epoxide’ and ‘MonoRed B’. However, additional removal of the double bond lacking in ‘MonoRed B’ from ‘MonoRed A’ did not further reduce the bioactivity of resulting variant ‘BisRed’, thus confirming the critical importance of the endocyclic enone for the overall bioactivity of the molecule. Moreover, the ability to trigger Nrf2 signaling and downstream anti-inflammatory effects appeared to be restricted to cyclopentenone-containing OxPL. For example, other prominent lipid mediators involved in the regulation of inflammation that lack a cyclopentenone moiety, such as the arachidonic acid-derived prostacyclin and lipoxin B4, or the omega-3 fatty acid-derived resolvin D2, neither affected the cytokine production nor triggered Nrf2 signaling in myeloid cells in our bioassay (Supplementary Figs S5 and S6). Taken together, these findings defined molecular determinants of the anti-inflammatory bioactivity of EC and provided a rationale to design customized EC variants as anti-inflammatory compounds with improved therapeutic potential.

Dose–response curves showing the modulation of R837-induced (5 μg/ml; 18 h) IL-12 secretion by prior treatment of BMDCs with the indicated synthetic lipids for 1 h.

Chemical structures and ΔEC50 values of the synthetic OxPL variant lipids analyzed in (A), presented relative to EC. Data information: Mean ± SEM of triplicate determinations are shown.

### Cyclo-EC, an EC variant with superior anti-inflammatory bioactivity *in vitro* and *in vivo*

**Figure 7:**
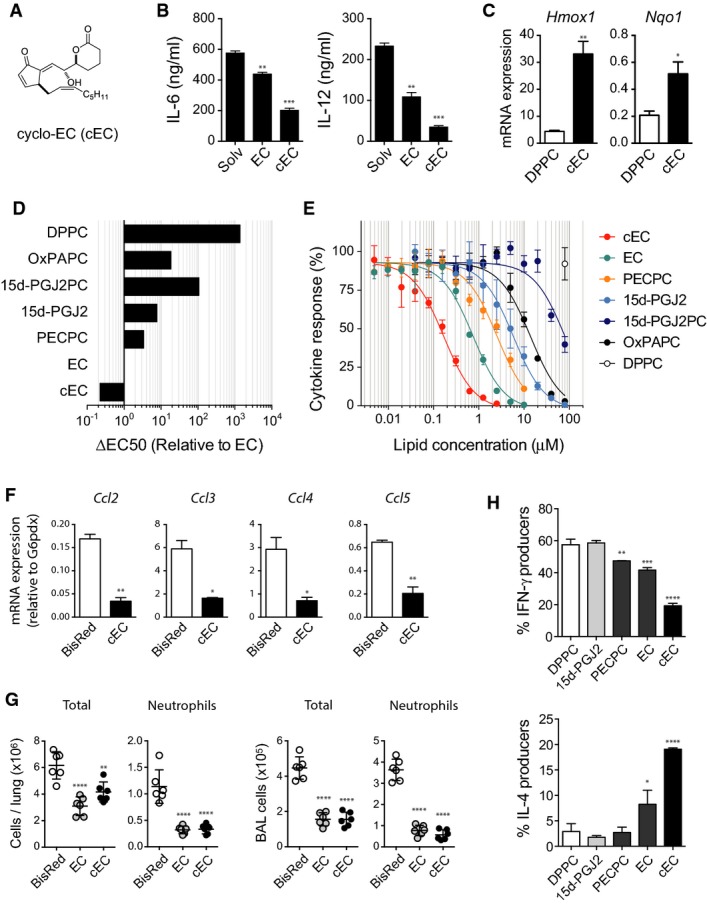
Cyclo-EC is an EC variant with superior anti-inflammatory bioactivity *in vitro* and *in vivo*

Considering the structural and functional similarity of EC and 15d-PGJ2, we also tested a series of chimeric molecules that combined features of EC and 15d-PGJ2, the synthesis of which is described in detail elsewhere (Egger *et al*, 2014). In this process, we developed the EC variant cyclo-EC (cEC), which hypothetically could be generated by an intra-molecular nucleophilic attack of the carboxylate-anion on one of the epoxide carbons, thus forming a 6-membered lactone ring (Fig7A) (Egger *et al*, 2014). Compared to EC, cEC exhibited superior anti-inflammatory capacity, as assessed by inhibition of IL-6 and IL-12 production (Fig7B), but also by the transcriptional regulation of IL-12, IL-6, IL-23, and TNFα (Supplementary Fig S7). The strong induction of several Nrf2 target genes including Hmox1 and Nqo1 indicated that like EC, also cEC triggered Nrf2 signaling to mediate these effects (Fig7C and Supplementary Fig S7). Moreover, cEC showed the most potent inhibition of IL-12 secretion among all fatty acid cyclopentenone OxPL tested (Fig7D and E). Expectedly, cEC also reduced the expression of pro-inflammatory chemokines (Fig7F) and decreased the infiltration of neutrophils into the lungs of LPS-challenged animals (Fig7G). The improved efficacy of cEC was further demonstrated by its superior capacity to modulate the DC-licensed T-cell differentiation *in vitro* (Fig7H). In particular, we observed that cEC still strongly biased the polarization of naïve T cells at concentrations at which EC only exhibited weak residual activity and no such effects could be detected for 15d-PGJ2 and PECPC. These data not only established cEC as a promising epoxycyclopentenone-derived compound to be further evaluated in the treatment of inflammatory disorders, but also recommended the class of epoxycyclopentenone-containing OxPL as potential basis for future anti-inflammatory therapeutics.

Chemical structure of the cyclic EC analog cyclo-EC.

Quantification of IL-6 and IL-12 secretion by BMDC treated with 250 nM cEC or EC for 60 min before stimulation with R837 (5 μg/ml; 18 h). Bars represent mean ± SEM from one of three independent experiments. **P < 0.01; ***P < 0.001; one-way ANOVA adjusted by Dunnett's multiple comparisons test.

mRNA expression of the Nrf2 targets Hmox1 and Nqo1 normalized to G6pdx expression. BMDCs were treated for 60 min with 500 nM cEC or DPPC followed by R837 stimulation (5 μg/ml) for 3 h. Data (mean ± SD) are representative of three independent experiments. *P < 0.05; **P < 0.01; unpaired two-tailed *t*-test.

ΔEC50 values of the indicated lipids and OxPL shown relative to that of EC as determined in (E).

Dose–response curves showing the modulation of R837-induced (5 μg/ml; 18 h) IL-12 secretion in BMDCs by prior treatment with the indicated OxPL derivatives for 1 h.

mRNA quantification of the indicated chemokines relative to G6pdx expression. BMDC were pretreated for 60 min with 500 nM cEC or the variant BisRed prior to R837 stimulation (5 μg/ml) for 3 h. Data (mean ± SD) are representative of three independent experiments. *P < 0.05; **P < 0.01; unpaired two-tailed *t*-test.

Quantification and characterization of cellular infiltrates in BAL. Groups of six C57BL/6 mice were pretreated i.t. with 50 μg cEC, EC, or BisRed 24 and 2 h before challenge with 150 ng/g LPS in the presence of 800 μg/g D-galactosamine. BAL was harvested 4 h after LPS injection, and inflammatory cells were characterized by FACS analysis. **P < 0.01; ****P < 0.0001; one-way ANOVA with Sidak's multiple comparisons test.

Comparison of the capacity of cEC, EC, and PECPC (1 μM) to license splenic dendritic cells to polarize naïve CD4 T cells into IFN-γ-producing (Th1) and IL-4-producing (Th2) effector cells. Data (mean ± SD) are representative of two independent experiments. One-way ANOVA adjusted by Dunnett's multiple comparisons test. *P < 0.05; **P < 0.01; ***P < 0.001; ****P < 0.0001.

## Discussion

In this study, we have characterized the potent anti-inflammatory activity of OxPAPC *in vitro* and *in vivo* and identified a distinct molecular OxPL species that mediates these effects by signaling via the oxidative stress-responsive transcription factor Nrf2. We show that this bioactivity is based on a common structural motif shared by anti-inflammatory OxPL and endogenous, pro-resolving lipid mediators, and generated a synthetic OxPL variant with increased anti-inflammatory potency.

Our understanding of the physiological significance of OxPL for biological processes has so far been greatly hampered by the experimental difficulties to monitor or even control the complexity and composition of experimentally produced OxPL mixtures. As a result, there is considerable discrepancy with regard to the reported biological effects of OxPL, as both pro-inflammatory and anti-inflammatory activities have been demonstrated. For example, the OxPL present in minimally oxidized LDL promote monocyte adhesion to the vascular endothelium in atherosclerosis (Watson *et al*, 1997) and induce the secretion of pro-inflammatory cytokines in endothelial cells (Subbanagounder *et al*, 2002). Furthermore, a direct recognition of OxPAPC by TLR4 or TLR2 has been shown to trigger severe inflammation *in vivo* (Imai *et al*, 2008; Kadl *et al*, 2011). Contrasting these observations, OxPAPC appears to inhibit the maturation and cytokine production of antigen-presenting cells *in vitro* (Blüml *et al*, 2005), and *in vivo* correlates of such activity have been reported for chronic inflammatory conditions, such as leprosy (Cruz *et al*, 2008) or dyslipidemia (Shamshiev *et al*, 2007), in which an increased generation of OxPL negatively influences disease outcomes. Still, the inhibitory effects of OxPAPC may also be beneficial for host survival by attenuating exacerbated immune activation, for example, during bacterial sepsis (Bochkov *et al*, 2002).

These conflicting findings clearly emphasize the importance of studying defined molecular OxPL species instead of bulk OxPAPC mixtures in order to elucidate their biological properties. We therefore chose a systematic approach to characterize the anti-inflammatory activity of OxPAPC. Starting from complex mixtures of *in vitro* generated OxPAPC preparations, we correlated the abundance of individual OxPL species to the overall bioactivity of the mixture and evaluated potential candidate lipids using synthetic compounds. In this process, we identified EC as the OxPL species that attenuates inflammatory responses through Nrf2 signaling. Its functional and structural homology to 15d-PGJ2 as well as the altered activity of variant lipids lacking specific electrophilic sites strongly suggested that the biological activities of OxPL are directly related to their chemical structure, for instance to the presence of the cyclopentenone motif in case of anti-inflammatory OxPL. This notion is supported by several studies reporting very specific bioactivities of defined OxPL, which are not shared by structurally unrelated OxPL. For example, the truncated OxPL POVPC and PGPC induce monocyte/endothelial cell interactions (Watson *et al*, 1997), but do not influence DC maturation (Cruz *et al*, 2008). Conversely, PEIPC has been reported to elicit IL-8 and MCP-1 secretion from endothelial cells by activating the EP2 receptor (Li *et al*, 2006), but also appears to negatively regulate CD1b expression and DC maturation in human leprosy (Cruz *et al*, 2008). In addition, PEIPC and PECPC were shown to restore endothelial barrier function *in vitro* (Birukov *et al*, 2004). Furthermore, KOdiAPC and structurally similar OxPL have been identified as a family of highly specific agonists for the scavenger receptor CD36 (Podrez *et al*, 2002) that triggers OxLDL uptake but also contributes to CD36-/TLR2-dependent apoptosis in macrophages (Seimon *et al*, 2010). Thus, diverse biological activities can be attributed to structurally distinct OxPL species, and several cellular receptors mediating such specific OxPL recognition have already been identified (Podrez *et al*, 2002; Li *et al*, 2006; Seimon *et al*, 2010). Our present study establishes Nrf2 signaling as an additional OxPL-sensing pathway that imparts the anti-inflammatory effects of a defined OxPL species. In particular, we found that the Nrf2-dependent inhibition of pro-inflammatory responses of myeloid cells was limited to OxPL containing an epoxycyclopentenone, but not shared by truncated OxPL such as POVPC, PGPC, or KOdiAPC. Furthermore, the observation that Nrf2-deficient DCs exhibited enhanced pro-inflammatory responses upon TLR stimulation implied an essential regulatory function of Nrf2 during inflammatory processes, presumably in response to endogenously formed OxPL or related prostanoids. This is in line with reports showing enhanced inflammatory responses and increased mortality of Nrf2-deficient mice during septic shock (Thimmulappa, 2006). Likewise, endogenous Nrf2 ligands, such as 15d-PGJ2, are generated during experimentally induced acute lung injury and have been shown to inhibit inflammation via Nrf2 (Itoh *et al*, 2003; Mochizuki *et al*, 2005). Importantly, relevant amounts of structurally related OxPL have been detected under chronic inflammatory conditions *in situ* (Bochkov *et al*, 2010) and were demonstrated to regulate antioxidant gene expression via Nrf2 signaling *in vivo* (Jyrkkänen *et al*, 2008).

In summary, we have identified from a complex mixture of OxPL a distinct OxPL species that efficiently suppresses the inflammatory responses of myeloid cells *in vitro* and *in vivo*. Our results provide insight to the essential structural characteristics and signaling of anti-inflammatory OxPL and demonstrate that both are shared with endogenous, pro-resolving lipid mediators. In addition, our structure–function studies facilitated the generation of an EC-derived variant with greatly enhanced bioactivity. Together, these findings not only highlight the potential of OxPL/Nrf2 signaling for the treatment of inflammatory disorders, but should also promote the development of novel anti-inflammatory compounds and of improved research tools to investigate the biology of OxPL in the future.

## Materials and Methods

### Mice, cells and reagents

Nrf2^−/−^ mice (Nfe2l2^tm1Mym^) (Itoh *et al*, 1997), backcrossed to C57BL/6 for more than eight generations, were obtained from the RIKEN BioResource Center, Japan. SMARTA mice (Tg(TcrLCMV)^Aox^) (Oxenius *et al*, 1998) were on a full C57BL/6 background. Pparg^fl/fl^CD11c-Cre mice were bred locally (using Pparg^tm1.2Mtz^ and Tg(Itgax-cre)^1-1Reiz^) ( Imai *et al*, 2004; Caton *et al*, 2007). The respective littermates were used as WT controls. Mice were age-/sex-matched and were taken into experiments when 6–12 weeks old. Randomization was not used to allocate animals to experimental groups. The group sizes for *in vivo* experiments were chosen based on prior experience and published literature in order to achieve the adequate statistical power. All animal experiments were performed according to institutional guidelines and Swiss federal regulations and were approved by the veterinary office of the Kanton of Zurich (permissions no. 167/2011 and 109/2012). Bone marrow cells from tibiae and femurs were isolated and differentiated *in vitro* into BMDC in the presence of 2 ng/ml recombinant mouse GM-CSF (BioLegend, 576308) in RPMI-1640 medium (supplemented with L-glutamine, HEPES, penicillin/streptomycin, and 10% FCS). Bone marrow-derived macrophages (BMDM) were differentiated from freshly isolated bone marrow cells in fully supplemented RPMI-1640 together with 10% L929-conditioned medium. Both cell types were harvested at day 7 and seeded at 10^5^ cells/well into 96-well round-bottom and flat-bottom plates, respectively. Primary splenic dendritic cells were obtained by 30-min collagenase IV digestion at 37°C followed by MACS sorting with anti-CD11c MicroBeads according to the manufacturer's instructions. Thioglycollate-elicited macrophages were harvested by peritoneal lavage 4 days after i.p. injection of 1 ml 3.8% thioglycollate broth. Cells were plated and adherent cells were used for *in vitro* bio-assays. SMARTA transgenic CD4^+^ T cells were obtained by processing whole spleens through 70-μm nylon cell strainers followed by separation with anti-CD4 MicroBeads.

### Lipids

PAPC (850459C) and DPPC (850355) were purchased from Avanti Polar lipids and stored in chloroform at −80°C. POVPC (10031), KOdiAPC (62945), and PGPC (10044) were all purchased from Cayman Chemicals. Lipids were stored at 10 mg/ml in EtOH at −20°C. The lipids EC, PECPC, EI, PEIPC, 15d-PGJ2, and 15d-PGJ2PC were synthesized as described (Egger *et al*, 2013, 2014). All lipids were stored at −80°C under nitrogen atmosphere. For experimental purposes, lipids were dissolved in DMSO to 20–50 mM in glass flasks (Carl Roth GmbH + Co) and then further diluted in pure RPMI-1640 medium before addition to cell suspensions. PAPC were oxidized by air exposure, with 5–10 μM iron(II)sulfate (Sigma, F8633-250G), or with 5–10 μM CuSO_4_ (Merck, 1.02790.0250) as oxidizing agents for various time intervals. Metal-catalyzed oxidation was performed in sterile PBS at 37°C in glass flasks using a rotary wheel set at 20 rpm/min.

### Mass spectrometric analysis

Data acquisition of oxidized PAPC species was performed by a Fourier transform ion cyclotron resonance mass spectrometer (FT-ICR-MS) (linear trap quadrupole-Fourier transform, LTQ-FT; Thermo Scientific) coupled to an UHPLC (Accela; Thermo Scientific) as described previously (Fauland *et al*, 2011). Briefly, chromatography was performed on C-18 reversed-phase HPLC and full-scan mass spectra were acquired at a resolution of 200,000 and < 2 ppm mass accuracy with external calibration. From the FT-ICR-MS preview scan, the five most abundant *m*/*z* values were picked in data-dependent acquisition (DDA) mode, fragmented in the linear ion trap analyzer, and ejected at nominal mass resolution. Analysis of high-resolution full-scan data of molecular ions was carried out by Lipid Data Analyzer (Hartler *et al*, 2011), while low-resolution MS/MS data were inspected manually for confirmation of molecular identity as described in more detail in the Supplementary Information (Supplementary Fig S8 and Supplementary Method).

### *In vitro* cytokine secretion assay

Unless otherwise stated, the experimental procedure for *in vitro* stimulation of lipid pulsed cells was performed as follows. Cells were counted and plated in 96-well plates at a density of 10^5^ cells/well. Macrophages were incubated for 2 h to allow for adherence, whereas dendritic cells were left to equilibrate at 37°C, 5% CO_2_ for 1 h. Subsequently, both cell types were pulsed for 60 min by addition of indicated concentrations of the various lipids dissolved in serum-free RPMI-1640 medium, as solubilization with BSA or incorporation into liposomes appeared not to enhance the observed bioactivity (Supplementary Fig S9). Cells were then washed twice with RPMI-1640 containing 2% FCS and stimulated with various TLR ligands for 18–20 h. Supernatants were removed, diluted if required, and subjected to ELISA for cytokine quantification.

### Lung inflammation models

C57BL/6 mice (Charles River) were injected with 50–500 μg EC/mouse 2 h prior to i.p. injection of ultra-pure LPS 0111:B4 (150 μg/kg; InvitroGen) and D-galactosamine (800 mg/kg; Carbosynth Limited) dissolved in PBS. After 4 h, mice were euthanized by injection of pentobarbital. Mice were perfused with 10 ml PBS to avoid contamination of lungs with blood leukocytes. Broncho-alveolar lavage (BAL) was performed by tracheal catheterization and repeated infusion and removal of 500 μl PBS. Lungs were paraffin-embedded for histological analysis or digested with 1 mg/ml collagenase IV (Bioconcept) in unsupplemented IMDM (1×) + GlutaMAX (Gibco Life Technologies). Digested lungs were processed through 70-μm nylon cell strainers. For flow cytometry, cells were counted and stained with antibodies against CD11b (BioLegend, 101228, 1:1,000), F4/80 (BioLegend, 123116 and 123120, 1:300), CD115 (eBioscience, 12-1152-81, 1:300), CD11c (eBioscience, 17-0114-82, 1:2,000), CD45 (BioLegend, 103114, 1:4,000), Siglec F (BD Bioscience, 562881, 1:300), Ly-6G (BioLegend, 127606, 1:300), Ly-6C (BioLegend, 128025, 1:2,000), GR-1 (eBioscience, 17-5931-82; 1:4,000), as well as NOS2 (Santa Cruz, sc-650, 1:500) and with the viability dye eFluor 780 (eBioscience, 65-0865-14, 1:4,000). Neutrophils were identified as eFluor780^−^CD45^+^CD11b^+^CD11c^−^Ly-6G^+^ cells. For intracellular stainings, cells were first fixed with 4% PFA for 5 min before permeabilization with 0.2% saponin. For histological analysis, lung sections were removed from PFA and embedded in paraffin. Leukocyte adhesion to the microvascular endothelium in lung was determined by morphometric image analysis of hematoxylin-stained paraffin sections. Inflammatory cell adhesion to the endothelium was counted in 15–20 vessels per mouse by an investigator blinded to the sample ID.

### Gene expression analysis

Tissues were lysed in 1 ml of TRIzol Reagent (Ambion Life Technologies), and mRNA was purified according to manufacturer's instructions. mRNA concentrations were measured with a NanoDrop device. Contaminating DNA was digested by RNase-free DNase (Life Technologies) and 2 μg mRNA/reaction was reverse-transcribed by GoScript™ Reverse Transcriptase (Promega) in the presence of RNase inhibitor (Bioconcept). Quantitative PCR was performed using KAPA SYBR FAST Bio-Rad iCycler Kit (Labgene Scientific, SA), and expression was normalized to *G6pdx* expression. Comparable results were obtained using other housekeeping genes as a reference (Supplementary Fig S10). The primer sets used in this study are shown as Supplementary Table S1.

### Co-culture experiments

CD4 T cells from SMARTA2 transgenic mice were obtained by MACS sorting of spleen with CD4^+^ beads (Miltenyi Biotec, 130-049-201). Dendritic cells were pulsed with the indicated concentrations of lipids for 60 min, washed with medium, and co-cultured together with CD4^+^ T cells in the presence of the cognate LCMV gp61-81 peptide for 4 days. Cells were then re-stimulated with PMA/ionomycin in the presence of brefeldin A for 4 h. Polarization of T cells was assessed by intracellular FACS staining for IL-4 (BioLegend, 504104, 1:300) and IFN-γ (BioLegend, 505810, 1:4,000).

**The paper explained**

**Problem**

Oxidative stress contributes to the pathogenesis of chronic inflammatory diseases, metabolic disorders, and cancer. The exposure of biological membranes to reactive oxygen species creates a complex mixture of distinct oxidized phospholipid (OxPL) species. It is now increasingly recognized that OxPL are not just by-products of lipid peroxidation associated with inflammatory conditions or increased oxidative stress, but instead actively modulate cellular signaling processes and contribute to the initiation and amplification of inflammation. However, the nature of the biologically active OxPL and the molecular mechanisms underlying their signaling remain largely unknown.

**Results**

Here, we characterize an anti-inflammatory bioactivity of OxPL that can be attributed to a specific category of OxPL. We show that this potent anti-inflammatory effect is mediated by the prostanoid-like OxPL component epoxycyclopentenone, which activates the transcription factor Nrf2 to inhibit pro-inflammatory cytokine and chemokine responses in myeloid cells *in vitro* and *in vivo*. Using a library of epoxycyclopentenone variants, we identified critical structural determinants of this bioactivity, thereby providing a molecular basis for the anti-inflammatory activity of lipid peroxidation products. Furthermore, we developed an epoxycyclopentenone-derived OxPL variant with an unprecedented anti-inflammatory bioactivity.

**Impact**

Our data provide insight to the essential structural characteristics and signaling of anti-inflammatory OxPL and demonstrate that both are shared with endogenous, pro-resolving lipid mediators. Together, these findings not only highlight the strategic potential of targeting OxPL/Nrf2 signaling in inflammation, but also suggest a novel class of highly bioactive compounds as promising therapeutic agents for the treatment of inflammatory diseases.

### Statistical analysis

All statistical tests were performed with GraphPad Prism version 6.0d for Mac OS X. For data sets with multiple comparisons and one control group, one-way ANOVA followed by Dunnett's correction for multiple comparisons was applied; for experiments with more than one control group, Sidak's correction was used. Equality of variances was tested using the Brown–Forsythe test. For comparison of means of two groups, the unpaired two-tailed Student's *t*-test was applied. The representation of the data as mean ± SD or SEM, the method of statistical evaluation as well as significance levels are indicated in each figure legend. For enhanced clarity, the exact *P*-values calculated throughout this study are provided in Supplementary Table S2.

## Supporting Information

Supplementary Figures S1–S10, Supplementary Method, Supplementary Tables S1, S2

Review Process File
