# Supplementary material for: Phospholipid oxidation generates potent anti-inflammatory lipid mediators that mimic structurally related pro-resolving eicosanoids by activating Nrf2
Source: EMBO Mol Med. 2015 Mar 13;7(5):593–607. doi: 10.15252/emmm.201404702 (PMC4492819; doi:10.15252/emmm.201404702)
Supplement: Supplementary file 1 [file emmm0007-0593-sd1.pdf]

Phospholipid oxidation generates potent anti-inflammatory lipid mediators that mimic structurally related pro-resolving eicosanoids by activating Nrf2

Peter Bretscher, Julian Egger, Abdijapar Shamshiev, Martin Trötzmüller, Harald Köfeler, Erick M. Carreira, Manfred Kopf, and Stefan Freigang

Supplementary information

Table of contents

Supplementary Figure S1. Anti-inflammatory bioactivity of OxPL and epoxycyclopentenone lipids is not caused by toxicity.

Supplementary Figure S2. Anti-inflammatory bioactivity of OxPL and epoxycyclopentenone lipids is not caused by toxicity.

Supplementary Figure S3. Enhanced inflammatory cytokine and chemokine responses of Nrf2-deficient as compared to wild type BMDC.

Supplementary Figure S4. Epoxycyclopentenone-containing OxPL induce Nrf2-mediated gene expression in vivo.

Supplementary Figure S5. The anti-inflammatory effect of OxPAPC is restricted to cyclopentenone-containing lipid mediators.

Supplementary Figure S6. The induction of Nrf2-signaling is restricted to cyclopentenone-containing lipid mediators.

Supplementary Figure S7. Epoxycyclopentenone lipids negatively regulate the expression of multiple cytokines.

Supplementary Figure S8 and supplementary method. High resolution mass chromatogram of m/z 666.397 ± 0.005 (upper panel) and low energy LTQ CID spectrum of P-HOdiA-PC at m/z 666.4 and a retention time of 4.90 min (lower panel).

Supplementary Figure S9. Effect of solubilization on the bioactivity of the cyclopentenone lipid 15d-PGJ2.

Supplementary Figure S10. Expression of housekeeping genes and absence of ER stress in cyclopentenone-stimulated cells.

Supplementary Figure Legends.

Supplementary Table 1. Sets of primers used for quantitative PCR in this study.

Supplementary Table 2. Exact p values.

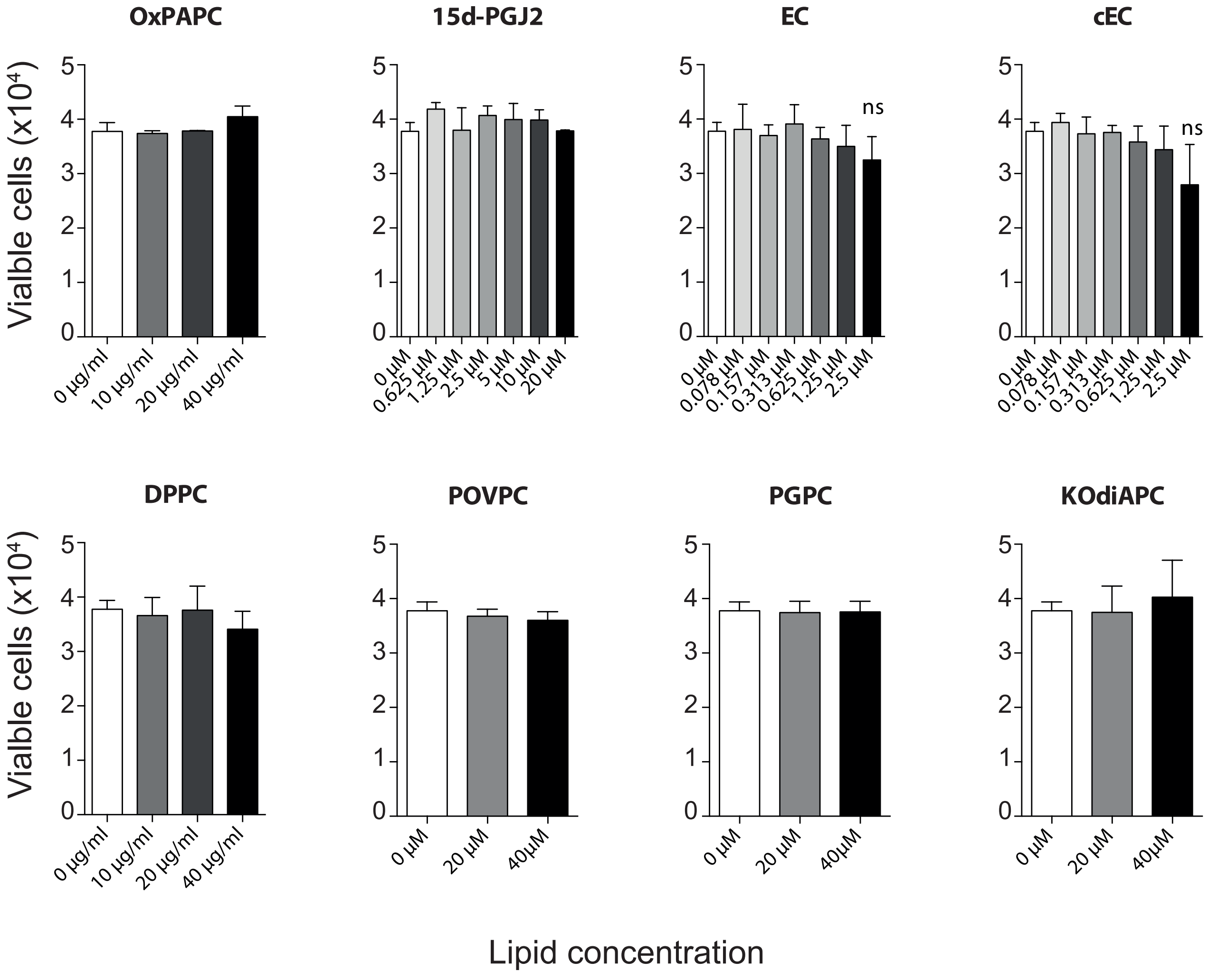

Bretscher et al., 2014 Supplementary Figure S1

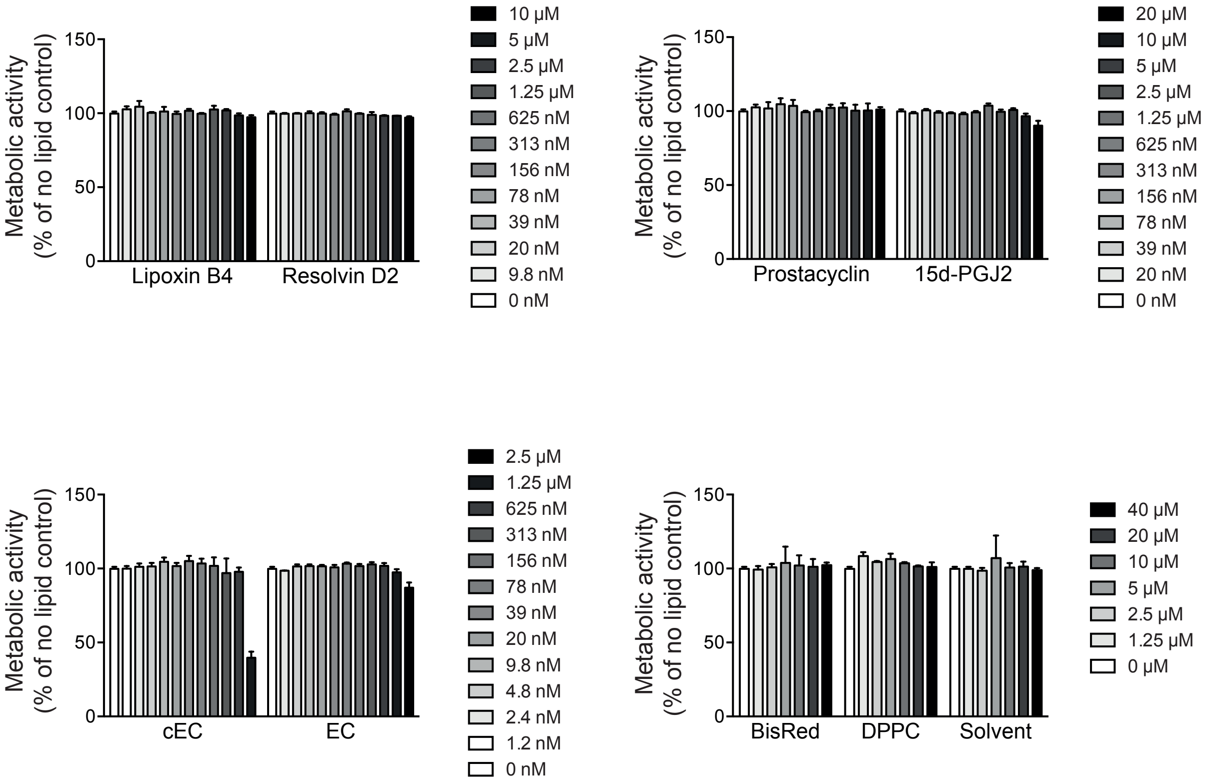

Bretscher et al., 2014 Supplementary Figure S2

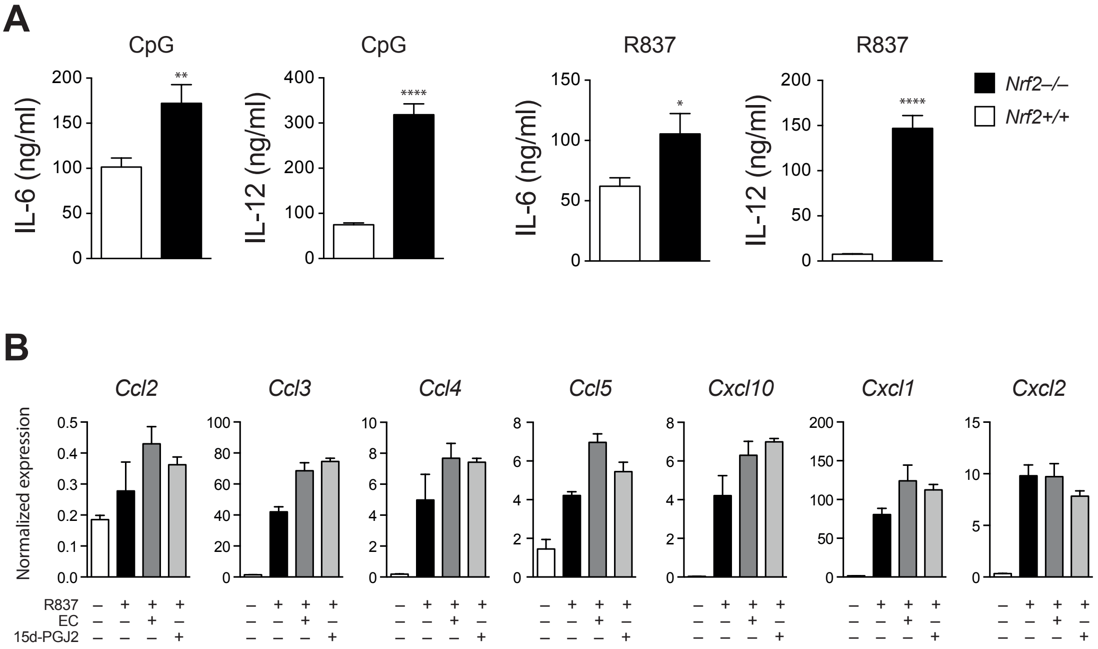

Bretscher et al., 2014

Supplementary Figure S3

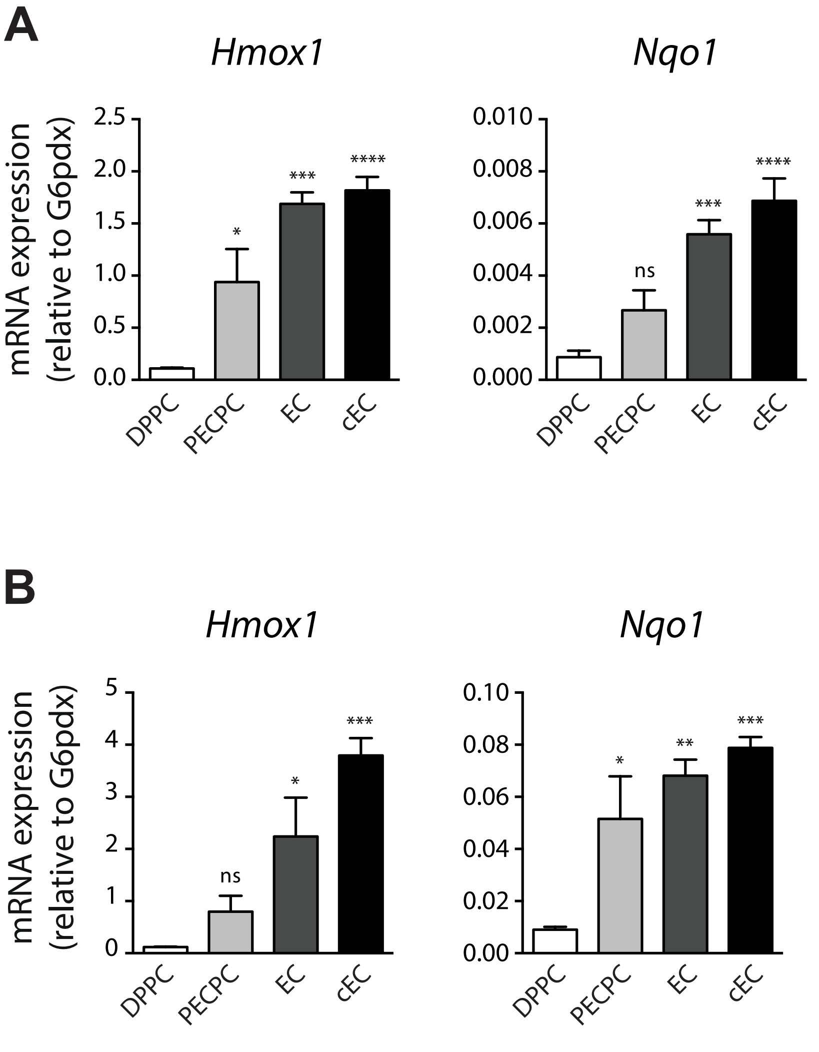

Bretscher et al., 2014 Supplementary Figure S4

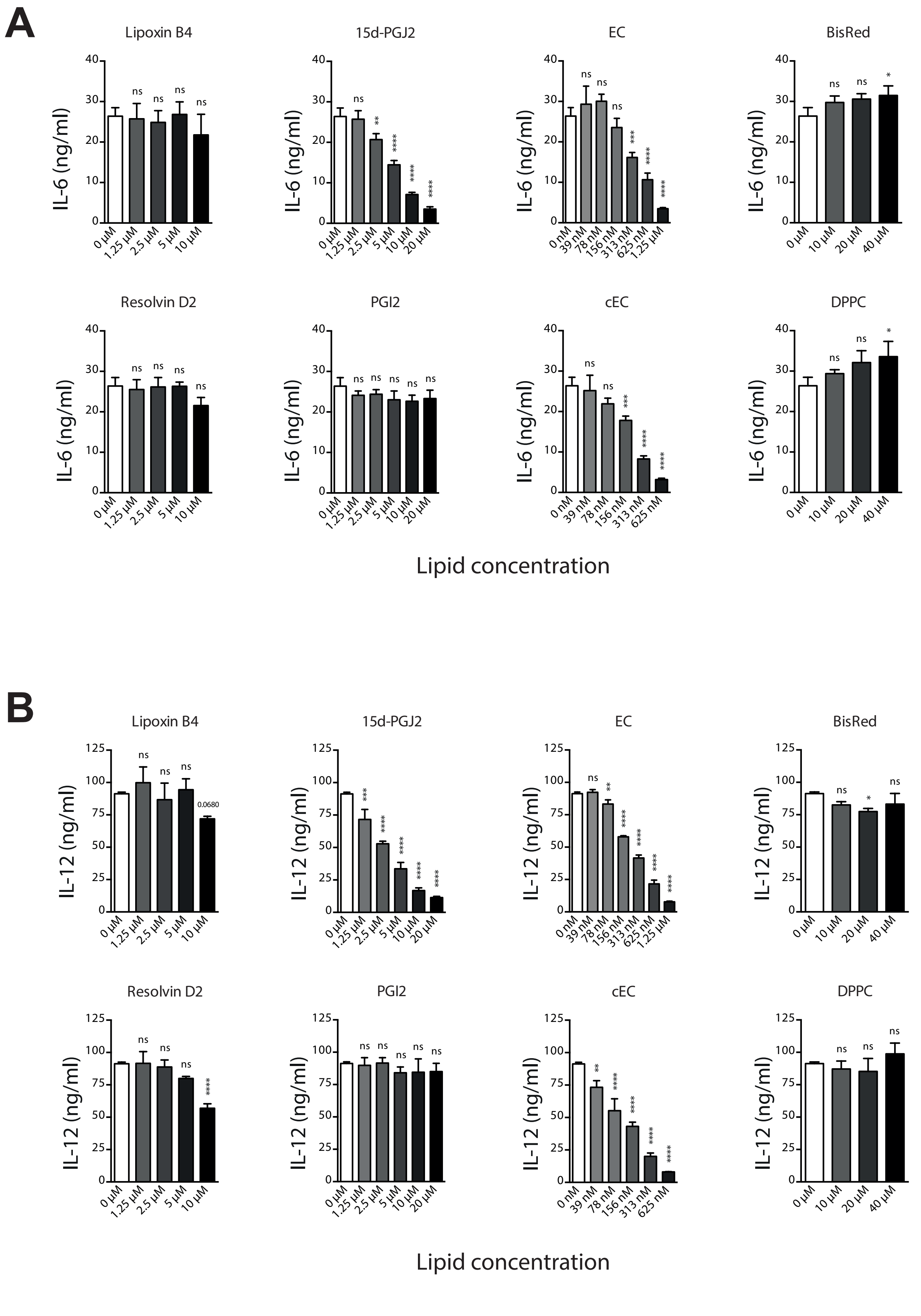
A
Lipoxin B4
15d-PGJ2
EC
BisRed
Resolvin D2
PGI2
cEC
DPPC
IL-6 (ng/ml)
0 μM
1.25 μM
2.5 μM
5 μM
10 μM
20 μM
40 μM
0 nM
39 nM
78 nM
156 nM
313 nM
625 nM
1.25 μM
ns
Lipid concentration
B
Lipoxin B4
15d-PGJ2
EC
BisRed
Resolvin D2
PGI2
cEC
DPPC
IL-12 (ng/ml)
0.0680
Lipid concentration

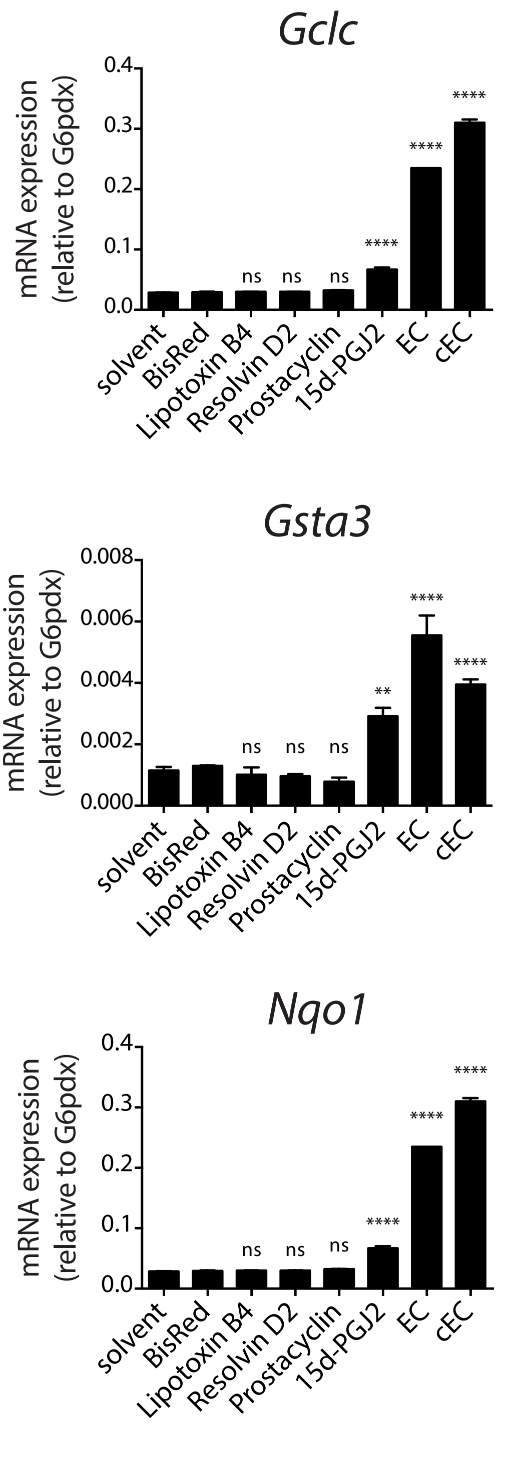

Bretscher et al., 2014 Supplementary Figure S6

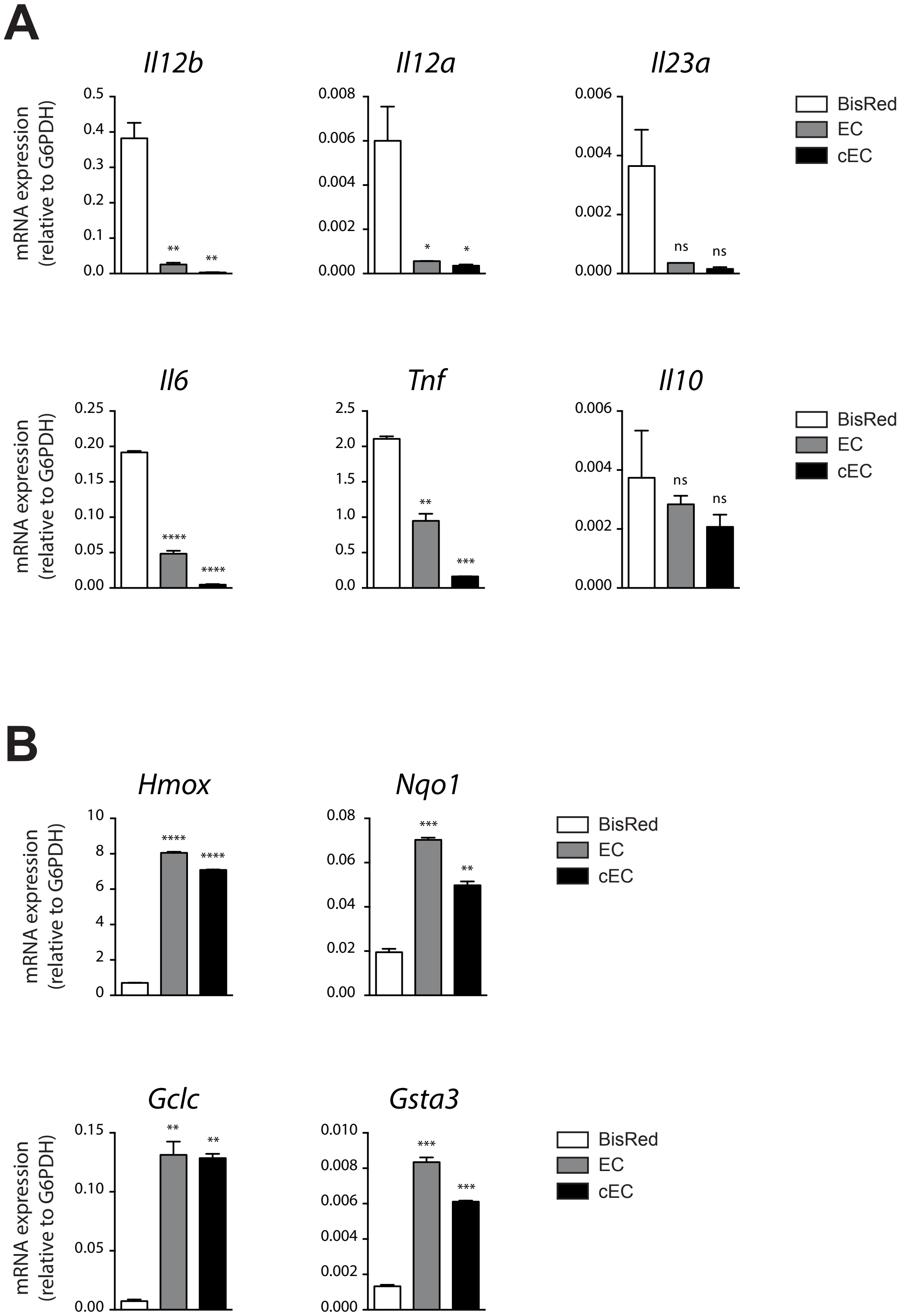

Bretscher et al., 2014 Supplementary Figure S7

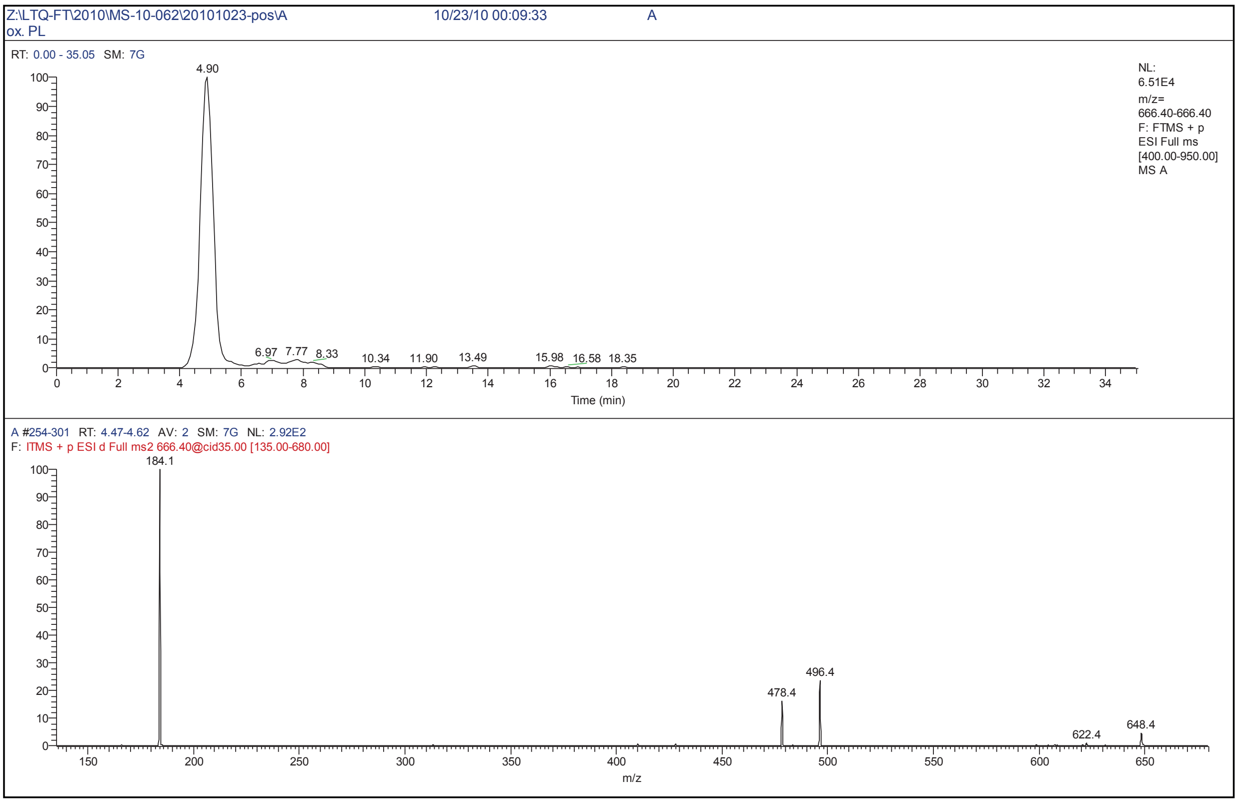

Bretscher et al., 2014 Supplementary Figure S8

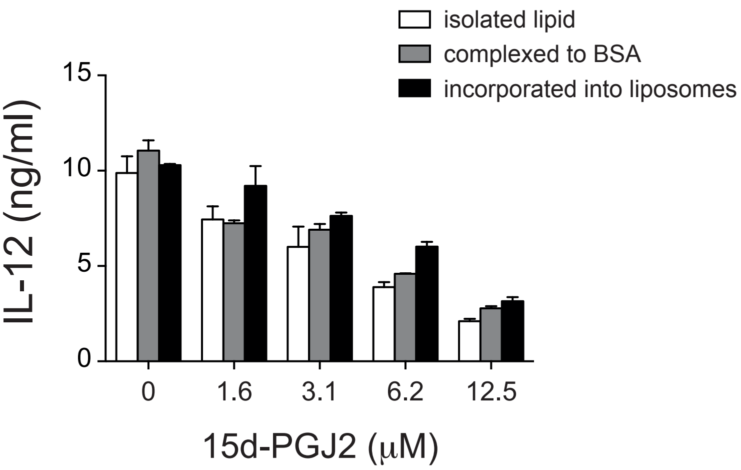

Bretscher et al., 2014 Supplementary Figure S9

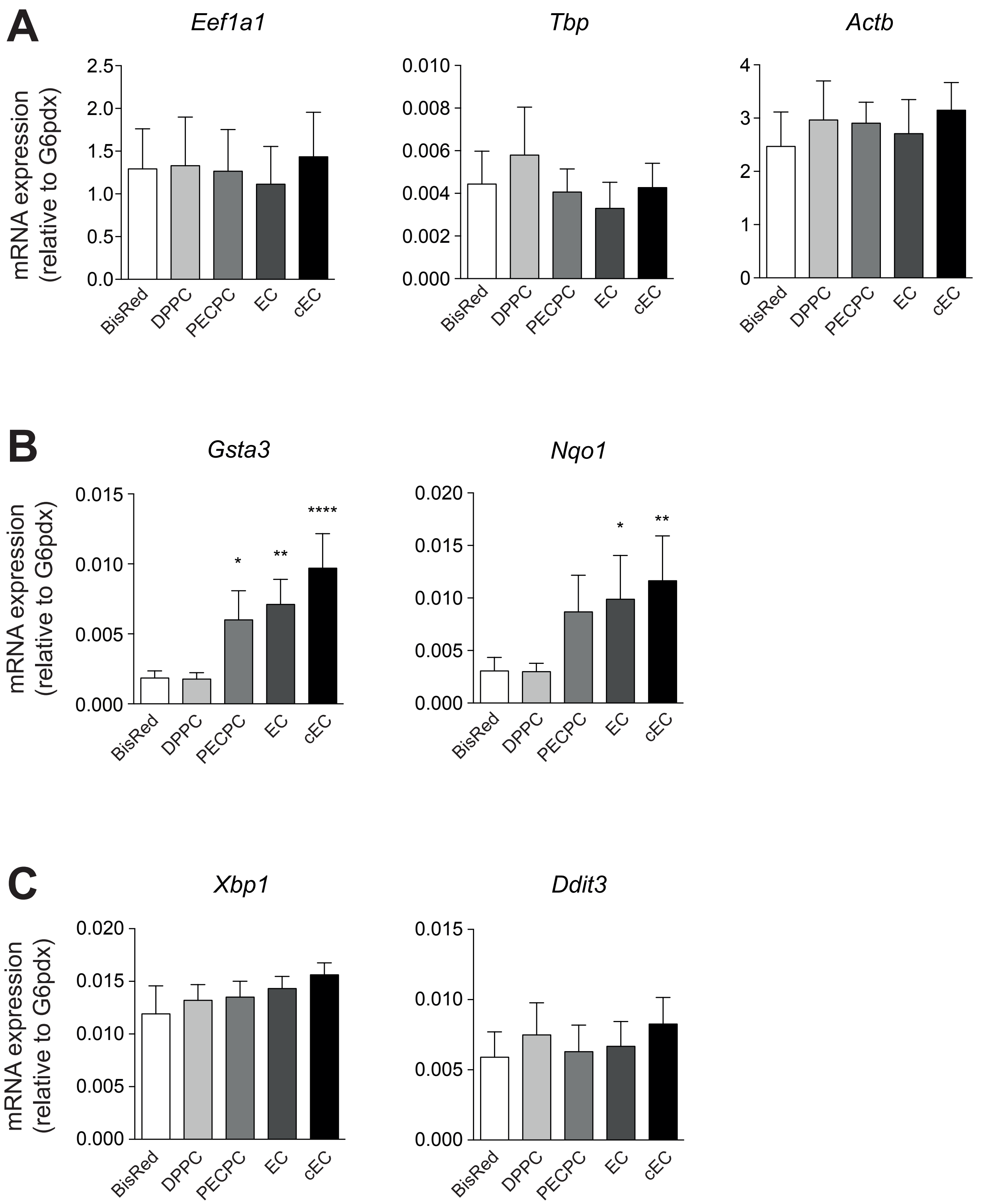

Bretscher et al., 2014 Supplementary Figure S10

Supplementary Figure Legends

Supplementary Figure S1. Anti-inflammatory bioactivity of OxPL and epoxycyclopentenone lipids is not caused by toxicity. Wild type BMDM were treated with indicated concentrations of lipids for 60 min prior to TLR7 stimulation with 5 μg/ml R837 for 18h. Cells were then harvested and stained with the viability dye eFluor780; and absolute numbers of viable, eFluor780-negative cells were determined by FACS. Data (mean ± SD) were analyzed by one-way ANOVA with Dunett's multiple comparisons test.

Supplementary Figure S2. Anti-inflammatory bioactivity of OxPL and epoxycyclopentenone lipids is not caused by toxicity.
Wild type BMDC were treated with indicated concentrations of lipids for 60 min prior to TLR7 stimulation with 5 μg/ml R837 for 18h. Metabolic activity was the determined using the alamar blue assay. Bars represent average ± SEM of triplicate cultures. One-way ANOVA adjusted by Dunnett's multiple comparisons test was performed to analyze statistical significance.

Supplementary Figure S3. Enhanced inflammatory cytokine and chemokine responses of Nrf2-deficient as compared to wild type BMDC.
(A) BMDCs of wild type and Nrf2-deficient mice were stimulated with R837 (5 μg/ml). Supernatants were harvested after 18h, and IL-12 levels were quantified by ELISA. (B) mRNA expression of indicated chemokines in Nrf2-deficient BMDC after treatment with EC (1 μM) or 15d-PGJ2 (20 μM) for 60 min followed by R837 (5 μg/ml) stimulation for 3h. Experiment was performed in parallel to Fig 4E. Expression levels are normalized to G6pdx.

Supplementary Figure S4. Epoxycyclopentenone-containing OxPL induce Nrf2-mediated gene expression in vivo.
(A, B) C57BL/6 mice were treated (i.t) with 50 μg PECPC, EC, cEC or control lipid DPPC. Three hours later, the expression of Nrf2 target genes Hmox1 and Nqo1 in BAL (A) and perfused lung (B) was quantified by real-time PCR. Expression levels are normalized to G6pdx. Bars represent mean ± SEM of groups of four animals. *, *P*

< 0.05; **, *P* < 0.01; ***, *P* < 0.001; ****, *P* < 0.0001; ns, not significant; as determined by one-way ANOVA adjusted by Dunnett's multiple comparisons test.

Supplementary Figure S5. The anti-inflammatory effect of OxPAPC is restricted to cyclopentenone-containing lipid mediators.
(A, B) Wild type BMDC were treated with titrated amounts of the indicated lipid mediators for 60 min prior to TLR7 stimulation with 5 μg/ml R837 for 18h. The concentrations of IL-6 (A) and IL-12 (B) in cell culture supernatants were determined by ELISA. Bars represent mean ± SD of triplicate cultures. *, *P* < 0.05; **, *P* < 0.01; ***, *P* < 0.001; ****, *P* < 0.0001; ns, not significant; as determined by one-way ANOVA adjusted by Dunnett's multiple comparisons test.

Supplementary Figure S6. The induction of Nrf2-signaling is restricted to cyclopentenone-containing lipid mediators.
Wild type BMDC were treated with the indicated lipid mediators for 60 min prior to TLR7 stimulation with 5 μg/ml R837 for 2.5h. The mRNA expression of the Nrf2 targets Gclc, Gsta3 and Nqo1 was quantified by quantitative real-time PCR. Gene expression levels were normalized to G6pdx and are shown as mean ± SD. **, *P* < 0.01; ****, *P* < 0.0001; ns, not significant; as determined by one-way ANOVA adjusted by Sidak's multiple comparisons test.

Supplementary Figure S7. Epoxycyclopentenone lipids negatively regulate the expression of multiple cytokines.
(A, B) Wild type BMDC were treated with EC, cEC or BisRed (all 1 μM) for 60 min prior TLR7 ligation with R837 (5 μg/ml) for 2h. The mRNA expression of (A) the cytokines IL-12, IL-23, IL-6, TNFα and IL-10 and of (B) the Nrf2 target genes Hmox1, Nqo1, Gclc and Gsta3 and Nqo1 was quantified by quantitative real-time PCR. Gene expression levels were normalized to G6pdx and are shown as mean ± SEM. ** *P* < 0.01; *** *P* < 0.001; **** *P* < 0.0001; ns, not significant; by one-way ANOVA adjusted by Dunnett's multiple comparisons test.

Supplementary Figure S8 and supplementary method. High resolution mass chromatogram of m/z 666.397 ± 0.005 (upper panel) and low energy LTQ CID spectrum of P-HOdiA-PC at m/z 666.4 and a retention time of 4.90 min (lower panel).

Identification of oxidized PAPC products was performed by high resolution LC-MS/MS in positive electrospray ionization according to the following selectivity criteria. High resolution FT-ICR mass spectra confirmed the elemental composition of molecular ions from which truncations and additional oxygen atoms in PAPC could be confirmed. The second criterion was retention time which was for oxidized species on a C-18 reversed phase column lower than for non-oxidized species. Oxidized PAPC species eluted in the retention time range between 3 and 10 min whereas non-oxidized phospholipids elute between 10 and 20 min with the chosen HPLC settings. The third selectivity criterion were linear ion trap collision induced dissociation (CID) spectra of the oxidized molecular ions previously determined in high resolution FT-ICR-MS. All spectra of oxidized species showed fragment ions at m/z 184, m/z 478 and m/z 496 corresponding to the choline phosphate headgroup, dehydrolized 16:0 LPC and 16:0 LPC. These three diagnostic fragments together with the elemental composition indicated the rest of the phospholipid to be an oxidized and / or truncated arachidonic acid. Since it is not possible to do a full structural elucidation including stereochemistry by low energy CID spectra (35% of maximum energy), we factored in all literature known characterized oxidized PAPC species, which is a comprehensive list of 30 compounds. With these pieces of information we matched the oxidized PAPC list with the elemental compositions of our mass chromatographic peaks. This process left mostly one in some cases a maximum of up to three possible underlying structures for each molecular elemental composition determined by FT-ICR-MS. Next we manually inspected each fragment spectrum for possible characteristic fragments of the oxidized fatty acid as paradigmatically exemplified for P-HOdiA-PC (Fig. S1). The protonated molecular weight of 666.39762 corresponds to the elemental composition $C_{32}H_{60}O_{11}N_1P_1$ which leaves $C_8H_{12}O_5$ for the oxidized fatty acid moiety after deduction of 16:0 LPC. According to literature this corresponds to HOdiA. Additionally the fragment m/z 648 indicates a neutral loss of H_2O derived from the hydroxy group and m/z 622 corresponds to a neutral loss of M.W. 44 indicative for decarboxylation, presumably at the terminal carboxylic group of HOdiA. These fragments confirmed the presence of HOdiA although it does not confirm the position of the double bond or the hydroxy group. This procedure of identification was performed for all other oxidized species. For presumably oxidized PAPC species with no literature match we only indicated the elemental composition.

Supplementary Figure S9. Effect of solubilization on the bioactivity of the cyclopentenone lipid 15d-PGJ2.

Wild type BMDC were treated with equimolar concentrations of 15d-PGJ2 either as the free lipid, as lipid complexed to fatty acid-free BSA, or in form of lipid incorporated into 100 nm liposomes, for 60 min before activation of TLR7 with R837 (5 μg/ml, 18h). IL-12 production in culture supernatant was determined by ELISA. Bars represent mean ± SD with of duplicate experiments.

Supplementary Figure S10. Expression of housekeeping genes and absence of ER stress in cyclopentenone-stimulated cells.

(A,B,C) Wild type BMDM were treated with cEC (1 μM), EC (1 μM), PECPC (5 μM), BisRed (5 μM) and DPPC (5 μM) for 60 min prior TLR7 ligation with R837 (5 μg/ml) for 2h. Expression of (A) the housekeeping genes eukaryotic elongation factor 1A1 (Eef1a1), TATA-binding protein (Tbp) and β-actin (Actb); of (B) the Nrf2 targets Gsta3 and Nqo1; and (C) of the ER stress regulated genes X-box binding protein 1 (Xbp1) and C/EBP homologous protein (Chop) was determined by qPCR relative to G6pdx. Data represent mean ± SEM for at least triplicate cultures. *, $P < 0.05$; **, $P < 0.01$; ****, $P < 0.0001$; by one-way ANOVA adjusted by Dunett's multiple comparisons test.

gene	Primer fwd	Primer rev
Actb	5'-CCC TGA AGT ACC CCA TTG AAC-3'	5'-CTT TTC ACG GTT GGC CTT AG-3'
Ccl2	5’-AGG TCC CTG TCA TGC TTC TG-3'	5’-ATT GGG ATC ATC TTG CTG GT-3'
Ccl3	5’-AGA TTC CAC GCC AAT TCA TC-3'	5’-CCC AGG TCT CTT TGG AGT CA-3'
Ccl4	5’-TTC TGT GCT CCA GGG TTC TC-3'	5’-AGC AAA GAC TGC TGG TCT CA-3'
Ccl5	5’-CGC ACC TGC CTC ACC ATA-3'	5’-CTG CAA GAT TGG AGC ACT TG-3'
Cxcl1	5’-GCC TAT CGC CAA TGA GCT G-3'	5’-ATT CTT GAG TGT GGC TAT GA-3'
Cxcl10	5’-AAG TGC TGC CGT CAT TTT CT-3'	5’-CCT ATG GCC CTC ATT CTC AC-3'
Cxcl2	5’-AGT GAA CTG CGC TGT CAA TG-3'	5’-GCC CTT GAG AGT GGC TAT GAC-3'
Ddit3	5'-AGC GAC AGA GCC AGA ATA AC-3'	5'-CCA GGT TCT GCT TTC AGG-3'
Eef1a1	5’-TCCACTTGGTCGCTTTGCT-3’	5’-CTT CTT GTC CAC AGC TTT GAT GA-3’
G6pdx	5'-ATG GTG AAG GTC GGT GTG AA-3'	5'-TAG ACC ATG TAG TTG AGG TC-3'
Gclc	5’-AAC AAG AAA CAT CCG GCA TC-3’	5’-CGT AGC CTC GGT AAA ATG GA-3’
Gsta3	5’-GCA CTT GCT GGA ACA TCA GA-3’	5’-TAC TTT GAT GGC AGG GGA AG-3’
Hmox-1	5'-TGC TCG AAT GAA CAC TCT GG-3'	5'-TCC TCT GTC ACG ATC ACC TG-3'
IL10	5'-GCA GGA CTT TAA GGG TTA CTT G-3'	5'-GAG GGT CTT CAG CTT CTC AC-3'
Il12a	5'-ATG TGT CAA TCA CGC TAC CTC-3'	5'-ACC ATG TCA TCT GTG GTC TTC-3'
Il12b	5'-TCA TCA GGG ACA TCA TCA AAC-3'	5'-TTG AGG GAG AAG TAG GAA TGG-3'
Il23	5`-CAC CTC CCT ACT AGG ACT CAG C-3`	5`-CTG CCA CTG CTG ACT AGA AC-3`
Il6	5'-TTC CAT CCA GT TGCC TTC TTG -3'	5'-TCA TTT CCA CGA TTT CCC AGA G-3'
Nqo1	5'-TTA CAG CAT TGG CCA CAC TC-3'	5'-GGC TGC TTG GAG CAA AAT AG-3'
Tbp	5'-TTG ACC TAA AGA CCA TTG CAC TTC-3'	5'-TTC TCA TGA TGA CTG CAG CAA A-3'
Tnf	5'-CCA AAG GGA TGA GAA GTT CC-3'	5'-GGG CCA TAG AAC TGA TGA GAG-3'
Xbp1	5'-GAT CCT GAC GAG GTT CCA GA-3'	5'-ACA GGG TCC AAC TTG TCC AG-3'

Figure	Panel	Genotype	Active Lipid	Read-out	Stimulus	Comparison	SD / SEM	n (replicates)	n (experiments)	Statistical Test	Exact p value	Stars
1	a	wt	OxPAPC	IL-6	R837	OxPAPC vs DMSO	SD	3	n > 3	1-way ANOVA, Dunnett	< 0.0001	****
		wt	OxPAPC	IL-6	R837	OxPAPC vs DPPC	SD	3		1-way ANOVA, Dunnett	< 0.0001	****
		wt	OxPAPC	IL-12	R837	OxPAPC vs DMSO	SD	3		1-way ANOVA, Dunnett	< 0.0001	****
		wt	OxPAPC	IL-12	R837	OxPAPC vs DPPC	SD	3		1-way ANOVA, Dunnett	< 0.0001	****
1	b	wt	OxPAPC	IL-6	R837	OxPAPC vs DPPC	SD	2	n = 3	unpaired, two-tailed t-test	0.0095	**
		wt	OxPAPC	IL-12	R837	OxPAPC vs DPPC	SD	2		unpaired, two-tailed t-test	0.0015	**
1	c	wt	OxPAPC	IL-6	R837	Nil vs. $CuSO_4$	SEM	3	n = 2	1-way ANOVA, Dunnett	< 0.0001	****
		wt	OxPAPC	IL-6	R837	Nil vs. $FeSO_4$	SEM	3		1-way ANOVA, Dunnett	< 0.0001	****
		wt	OxPAPC	IL-6	R837	Nil vs. O_2	SEM	3		1-way ANOVA, Dunnett	< 0.0001	****
		wt	OxPAPC	IL-12	R837	Nil vs. $CuSO_4$	SEM	3		1-way ANOVA, Dunnett	< 0.0001	****
		wt	OxPAPC	IL-12	R837	Nil vs. $FeSO_4$	SEM	3		1-way ANOVA, Dunnett	< 0.0001	****
		wt	OxPAPC	IL-12	R837	Nil vs. O_2	SEM	3		1-way ANOVA, Dunnett	< 0.0001	****
1	e	wt	OxPAPC	IL-6	LTA	0 ug/ml vs. 40 ug/ml	SD	3	n = 3	1-way ANOVA, Dunnett	< 0.0001	****
		wt	OxPAPC	IL-6	LTA	0 ug/ml vs. 20 ug/ml	SD	3		1-way ANOVA, Dunnett	< 0.0001	****
		wt	OxPAPC	IL-6	Poly I:C	0 ug/ml vs. 40 ug/ml	SD	3		1-way ANOVA, Dunnett	0.0011	**
		wt	OxPAPC	IL-6	Poly I:C	0 ug/ml vs. 20 ug/ml	SD	3		1-way ANOVA, Dunnett	0.0404	*
		wt	OxPAPC	IL-6	LPS	0 ug/ml vs. 40 ug/ml	SD	3		1-way ANOVA, Dunnett	0.0004	***
		wt	OxPAPC	IL-6	LPS	0 ug/ml vs. 20 ug/ml	SD	3		1-way ANOVA, Dunnett	0.0658	ns
		wt	OxPAPC	IL-6	R837	0 ug/ml vs. 40 ug/ml	SD	3		1-way ANOVA, Dunnett	0.0013	**
		wt	OxPAPC	IL-6	R837	0 ug/ml vs. 20 ug/ml	SD	3		1-way ANOVA, Dunnett	0.0298	*
		wt	OxPAPC	IL-6	CpG	0 ug/ml vs. 40 ug/ml	SD	3		1-way ANOVA, Dunnett	< 0.0001	****
		wt	OxPAPC	IL-6	CpG	0 ug/ml vs. 20 ug/ml	SD	3		1-way ANOVA, Dunnett	< 0.0001	****
1	f	wt	OxPAPC	IL-12	LTA	0 ug/ml vs. 40 ug/ml	SD	3	n = 3	1-way ANOVA, Dunnett	< 0.0001	****
		wt	OxPAPC	IL-12	LTA	0 ug/ml vs. 20 ug/ml	SD	3		1-way ANOVA, Dunnett	< 0.0001	****
		wt	OxPAPC	IL-12	Poly I:C	0 ug/ml vs. 40 ug/ml	SD	3		1-way ANOVA, Dunnett	0.0006	***
		wt	OxPAPC	IL-12	Poly I:C	0 ug/ml vs. 20 ug/ml	SD	3		1-way ANOVA, Dunnett	0.0109	*
		wt	OxPAPC	IL-12	LPS	0 ug/ml vs. 40 ug/ml	SD	3		1-way ANOVA, Dunnett	< 0.0001	****
		wt	OxPAPC	IL-12	LPS	0 ug/ml vs. 20 ug/ml	SD	3		1-way ANOVA, Dunnett	< 0.0001	****
		wt	OxPAPC	IL-12	R837	0 ug/ml vs. 40 ug/ml	SD	3		1-way ANOVA, Dunnett	< 0.0001	****
		wt	OxPAPC	IL-12	R837	0 ug/ml vs. 20 ug/ml	SD	3		1-way ANOVA, Dunnett	0.0002	***
		wt	OxPAPC	IL-12	CpG	0 ug/ml vs. 40 ug/ml	SD	3		1-way ANOVA, Dunnett	< 0.0001	****
		wt	OxPAPC	IL-12	CpG	0 ug/ml vs. 20 ug/ml	SD	3		1-way ANOVA, Dunnett	0.0002	***
1	h	wt	OxPAPC	IL-4	gp61 peptide	OxPAPC vs DPPC	SD	2	n > 3	unpaired, two-tailed t-test	0.0202	*
		wt	OxPAPC	IFNg	gp61 peptide	OxPAPC vs DPPC	SD	2		unpaired, two-tailed t-test	0.0151	*
1	i	wt	OxPAPC	IFNg	gp61 peptide	0 µg/ml vs. 64 µg/ml	SD	2	n = 2	1-way ANOVA, Dunnett	< 0.0001	****
		wt	OxPAPC	IFNg	gp61 peptide	0 µg/ml vs. 32 µg/ml	SD	2		1-way ANOVA, Dunnett	< 0.0001	****
		wt	OxPAPC	IFNg	gp61 peptide	0 µg/ml vs. 16 µg/ml	SD	2		1-way ANOVA, Dunnett	< 0.0001	****
		wt	OxPAPC	IFNg	gp61 peptide	0 µg/ml vs. 8 µg/ml	SD	2		1-way ANOVA, Dunnett	0.0052	**
		wt	DPPC	IFNg	gp61 peptide	0 µg/ml vs. 64 µg/ml	SD	2		1-way ANOVA, Dunnett	0.9999	ns
		wt	DPPC	IFNg	gp61 peptide	0 µg/ml vs. 32 µg/ml	SD	2		1-way ANOVA, Dunnett	0.9979	ns
		wt	DPPC	IFNg	gp61 peptide	0 µg/ml vs. 16 µg/ml	SD	2		1-way ANOVA, Dunnett	0.927	ns
		wt	DPPC	IFNg	gp61 peptide	0 µg/ml vs. 8 µg/ml	SD	2		1-way ANOVA, Dunnett	0.9947	ns
3	a	wt	OxPAPC	IL-6	R837	DMSO vs OxPAPC	SD	3	n = 3	1-way ANOVA, Sidak	< 0.0001	****
		wt	PEIPC	IL-6	R837	DMSO vs. PEIPC	SD	3		1-way ANOVA, Sidak	< 0.0001	****
		wt	PECPC	IL-6	R837	DMSO vs. PECPC	SD	3		1-way ANOVA, Sidak	< 0.0001	****

		wt	POVPC	IL-6	R837	DMSO vs. POVPC	SD	3		1-way ANOVA, Sidak	0.9855	ns
		wt	PGPC	IL-6	R837	DMSO vs. PGPC	SD	3		1-way ANOVA, Sidak	0.9828	ns
		wt	KOdiAPC	IL-6	R837	DMSO vs. KOdiAPC	SD	3		1-way ANOVA, Sidak	< 0.0001	****
		wt	OxPAPC	IL-6	R837	DPPC vs. OxPAPC	SD	3		1-way ANOVA, Sidak	< 0.0001	****
		wt	PEIPC	IL-6	R837	DPPC vs. PEIPC	SD	3		1-way ANOVA, Sidak	< 0.0001	****
		wt	PECPC	IL-6	R837	DPPC vs. PECPC	SD	3		1-way ANOVA, Sidak	< 0.0001	****
		wt	POVPC	IL-6	R837	DPPC vs. POVPC	SD	3		1-way ANOVA, Sidak	0.9736	ns
		wt	PGPC	IL-6	R837	DPPC vs. PGPC	SD	3		1-way ANOVA, Sidak	0.9694	ns
		wt	KOdiAPC	IL-6	R837	DPPC vs. KOdiAPC	SD	3		1-way ANOVA, Sidak	< 0.0001	****
		wt	OxPAPC	IL-12	R837	DMSO vs. OxPAPC	SD	3		1-way ANOVA, Sidak	< 0.0001	ns
		wt	PEIPC	IL-12	R837	DMSO vs. PEIPC	SD	3		1-way ANOVA, Sidak	< 0.0001	****
		wt	PECPC	IL-12	R837	DMSO vs. PECPC	SD	3		1-way ANOVA, Sidak	< 0.0001	****
		wt	POVPC	IL-12	R837	DMSO vs. POVPC	SD	3		1-way ANOVA, Sidak	0.0643	****
		wt	PGPC	IL-12	R837	DMSO vs. PGPC	SD	3		1-way ANOVA, Sidak	0.7859	ns
		wt	KOdiAPC	IL-12	R837	DMSO vs. KOdiAPC	SD	3		1-way ANOVA, Sidak	> 0.9999	ns
		wt	OxPAPC	IL-12	R837	DPPC vs. OxPAPC	SD	3		1-way ANOVA, Sidak	< 0.0001	ns
		wt	PEIPC	IL-12	R837	DPPC vs. PEIPC	SD	3		1-way ANOVA, Sidak	< 0.0001	****
		wt	PECPC	IL-12	R837	DPPC vs. PECPC	SD	3		1-way ANOVA, Sidak	< 0.0001	****
		wt	POVPC	IL-12	R837	DPPC vs. POVPC	SD	3		1-way ANOVA, Sidak	0.9002	****
		wt	PGPC	IL-12	R837	DPPC vs. PGPC	SD	3		1-way ANOVA, Sidak	> 0.9999	ns
		wt	KOdiAPC	IL-12	R837	DPPC vs. KOdiAPC	SD	3		1-way ANOVA, Sidak	0.3455	ns
3	e	wt	EC	IL-12	LPS	Med vs. EC	SEM	3	n = 3	1-way ANOVA, Dunnett	< 0.0001	****
		wt	PECPC	IL-12	LPS	Med vs. PECPC	SEM	3		1-way ANOVA, Dunnett	0.0003	***
		wt	15d-PGJ2	IL-12	LPS	Med vs. 15d-PGJ2	SEM	3		1-way ANOVA, Dunnett	< 0.0001	****
		wt	15d-PGJ2PC	IL-12	LPS	Med vs. 15d-PGJ2PC	SEM	3		1-way ANOVA, Dunnett	0.0013	**
		wt	EC	IL-12	CpG	Med vs. EC	SEM	3		1-way ANOVA, Dunnett	< 0.0001	****
		wt	PECPC	IL-12	CpG	Med vs. PECPC	SEM	3		1-way ANOVA, Dunnett	< 0.0001	****
		wt	15d-PGJ2	IL-12	CpG	Med vs. 15d-PGJ2	SEM	3		1-way ANOVA, Dunnett	< 0.0001	****
		wt	15d-PGJ2PC	IL-12	CpG	Med vs. 15d-PGJ2PC	SEM	3		1-way ANOVA, Dunnett	< 0.0001	****
		wt	EC	IL-12	R837	Med vs. EC	SEM	3		1-way ANOVA, Dunnett	< 0.0001	****
		wt	PECPC	IL-12	R837	Med vs. PECPC	SEM	3		1-way ANOVA, Dunnett	< 0.0001	****
		wt	15d-PGJ2	IL-12	R837	Med vs. 15d-PGJ2	SEM	3		1-way ANOVA, Dunnett	< 0.0001	****
		wt	15d-PGJ2PC	IL-12	R837	Med vs. 15d-PGJ2PC	SEM	3		1-way ANOVA, Dunnett	< 0.0001	****
4	a	pparg -/-	OxPAPC	IL-12	R837	no lipid vs. 40 ug/ml	SEM	3	n = 3	1-way ANOVA, Dunnett	< 0.0001	****
		pparg -/-	OxPAPC	IL-12	R837	no lipid vs. 20 ug/ml	SEM	3		1-way ANOVA, Dunnett	0.0003	***
		pparg +/+	OxPAPC	IL-12	R837	no lipid vs. 40 ug/ml	SEM	3		1-way ANOVA, Dunnett	< 0.0001	****
		pparg +/+	OxPAPC	IL-12	R837	no lipid vs. 20 ug/ml	SEM	3		1-way ANOVA, Dunnett	< 0.0001	****
		nrf2 -/-	OxPAPC	IL-12	R837	no lipid vs. 40 ug/ml	SEM	3		1-way ANOVA, Dunnett	0.7537	ns
		nrf2 -/-	OxPAPC	IL-12	R837	no lipid vs. 20 ug/ml	SEM	3		1-way ANOVA, Dunnett	0.0091	**
		nrf2 +/+	OxPAPC	IL-12	R837	no lipid vs. 40 ug/ml	SEM	3		1-way ANOVA, Dunnett	< 0.0001	****
		nrf2 +/+	OxPAPC	IL-12	R837	no lipid vs. 20 ug/ml	SEM	3		1-way ANOVA, Dunnett	< 0.0001	****
		pparg -/-	EC	IL-12	R837	no lipid vs. 1.25 uM	SEM	3		1-way ANOVA, Dunnett	< 0.0001	****
		pparg -/-	EC	IL-12	R837	no lipid vs. 0.625 uM	SEM	3		1-way ANOVA, Dunnett	< 0.0001	****
		pparg -/-	EC	IL-12	R837	no lipid vs. 0.313 uM	SEM	3		1-way ANOVA, Dunnett	< 0.0001	****
		pparg +/+	EC	IL-12	R837	no lipid vs. 1.25 uM	SEM	3		1-way ANOVA, Dunnett	< 0.0001	****
		pparg +/+	EC	IL-12	R837	no lipid vs. 0.625 uM	SEM	3		1-way ANOVA, Dunnett	< 0.0001	****
		pparg +/+	EC	IL-12	R837	no lipid vs. 0.313 uM	SEM	3		1-way ANOVA, Dunnett	< 0.0001	****
		nrf2 -/-	EC	IL-12	R837	no lipid vs. 1.25 uM	SEM	3		1-way ANOVA, Dunnett	0.1619	ns

		nrf2 -/-	EC	IL-12	R837	no lipid vs. 0.625 uM	SEM	3		1-way ANOVA, Dunnett	0.9752	ns
		nrf2 -/-	EC	IL-12	R837	no lipid vs. 0.313 uM	SEM	3		1-way ANOVA, Dunnett	0.9977	ns
		nrf2 +/+	EC	IL-12	R837	no lipid vs. 1.25 uM	SEM	3		1-way ANOVA, Dunnett	< 0.0001	****
		nrf2 +/+	EC	IL-12	R837	no lipid vs. 0.625 uM	SEM	3		1-way ANOVA, Dunnett	< 0.0001	****
		nrf2 +/+	EC	IL-12	R837	no lipid vs. 0.313 uM	SEM	3		1-way ANOVA, Dunnett	< 0.0001	****
		pparg -/-	POVPC	IL-12	R837	no lipid vs. 40 uM	SEM	3		1-way ANOVA, Dunnett	0.6435	ns
		pparg -/-	POVPC	IL-12	R837	no lipid vs. 20 uM	SEM	3		1-way ANOVA, Dunnett	0.2514	ns
		pparg -/-	POVPC	IL-12	R837	no lipid vs. 10 uM	SEM	3		1-way ANOVA, Dunnett	0.908	ns
		pparg +/+	POVPC	IL-12	R837	no lipid vs. 40 uM	SEM	3		1-way ANOVA, Dunnett	0.9996	ns
		pparg +/+	POVPC	IL-12	R837	no lipid vs. 20 uM	SEM	3		1-way ANOVA, Dunnett	0.9239	ns
		pparg +/+	POVPC	IL-12	R837	no lipid vs. 10 uM	SEM	3		1-way ANOVA, Dunnett	0.8813	ns
		nrf2 -/-	POVPC	IL-12	R837	no lipid vs. 40 uM	SEM	3		1-way ANOVA, Dunnett	0.0015	**
		nrf2 -/-	POVPC	IL-12	R837	no lipid vs. 20 uM	SEM	3		1-way ANOVA, Dunnett	0.003	**
		nrf2 -/-	POVPC	IL-12	R837	no lipid vs. 10 uM	SEM	3		1-way ANOVA, Dunnett	0.0028	**
		nrf2 +/+	POVPC	IL-12	R837	no lipid vs. 40 uM	SEM	3		1-way ANOVA, Dunnett	0.8039	ns
		nrf2 +/+	POVPC	IL-12	R837	no lipid vs. 20 uM	SEM	3		1-way ANOVA, Dunnett	0.6191	ns
		nrf2 +/+	POVPC	IL-12	R837	no lipid vs. 10 uM	SEM	3		1-way ANOVA, Dunnett	> 0.9999	ns
		pparg -/-	PGPC	IL-12	R837	no lipid vs. 40 uM	SEM	3		1-way ANOVA, Dunnett	0.0493	*
		pparg -/-	PGPC	IL-12	R837	no lipid vs. 20 uM	SEM	3		1-way ANOVA, Dunnett	0.391	ns
		pparg -/-	PGPC	IL-12	R837	no lipid vs. 10 uM	SEM	3		1-way ANOVA, Dunnett	0.9843	ns
		pparg +/+	PGPC	IL-12	R837	no lipid vs. 40 uM	SEM	3		1-way ANOVA, Dunnett	0.1661	ns
		pparg +/+	PGPC	IL-12	R837	no lipid vs. 20 uM	SEM	3		1-way ANOVA, Dunnett	> 0.9999	ns
		pparg +/+	PGPC	IL-12	R837	no lipid vs. 10 uM	SEM	3		1-way ANOVA, Dunnett	0.9789	ns
		nrf2 -/-	PGPC	IL-12	R837	no lipid vs. 40 uM	SEM	3		1-way ANOVA, Dunnett	0.0148	*
		nrf2 -/-	PGPC	IL-12	R837	no lipid vs. 20 uM	SEM	3		1-way ANOVA, Dunnett	0.016	*
		nrf2 -/-	PGPC	IL-12	R837	no lipid vs. 10 uM	SEM	3		1-way ANOVA, Dunnett	0.0539	ns
		nrf2 +/+	PGPC	IL-12	R837	no lipid vs. 40 uM	SEM	3		1-way ANOVA, Dunnett	0.0161	*
		nrf2 +/+	PGPC	IL-12	R837	no lipid vs. 20 uM	SEM	3		1-way ANOVA, Dunnett	0.1546	ns
		nrf2 +/+	PGPC	IL-12	R837	no lipid vs. 10 uM	SEM	3		1-way ANOVA, Dunnett	0.9645	ns
		pparg -/-	KOdiAPC	IL-12	R837	no lipid vs. 40 uM	SEM	3		1-way ANOVA, Dunnett	0.682	ns
		pparg -/-	KOdiAPC	IL-12	R837	no lipid vs. 20 uM	SEM	3		1-way ANOVA, Dunnett	0.2602	ns
		pparg -/-	KOdiAPC	IL-12	R837	no lipid vs. 10 uM	SEM	3		1-way ANOVA, Dunnett	> 0.9999	ns
		pparg +/+	KOdiAPC	IL-12	R837	no lipid vs. 40 uM	SEM	3		1-way ANOVA, Dunnett	0.9253	ns
		pparg +/+	KOdiAPC	IL-12	R837	no lipid vs. 20 uM	SEM	3		1-way ANOVA, Dunnett	0.7503	ns
		pparg +/+	KOdiAPC	IL-12	R837	no lipid vs. 10 uM	SEM	3		1-way ANOVA, Dunnett	0.8048	ns
		nrf2 -/-	KOdiAPC	IL-12	R837	no lipid vs. 40 uM	SEM	3		1-way ANOVA, Dunnett	0.0008	***
		nrf2 -/-	KOdiAPC	IL-12	R837	no lipid vs. 20 uM	SEM	3		1-way ANOVA, Dunnett	0.0021	**
		nrf2 -/-	KOdiAPC	IL-12	R837	no lipid vs. 10 uM	SEM	3		1-way ANOVA, Dunnett	0.001	***
		nrf2 +/+	KOdiAPC	IL-12	R837	no lipid vs. 40 uM	SEM	3		1-way ANOVA, Dunnett	0.5674	ns
		nrf2 +/+	KOdiAPC	IL-12	R837	no lipid vs. 20 uM	SEM	3		1-way ANOVA, Dunnett	0.4192	ns
		nrf2 +/+	KOdiAPC	IL-12	R837	no lipid vs. 10 uM	SEM	3		1-way ANOVA, Dunnett	0.4722	ns
		pparg -/-	15d-PGJ2	IL-12	R837	no lipid vs. 20 uM	SEM	3		1-way ANOVA, Dunnett	< 0.0001	****
		pparg -/-	15d-PGJ2	IL-12	R837	no lipid vs. 10 uM	SEM	3		1-way ANOVA, Dunnett	< 0.0001	****
		pparg -/-	15d-PGJ2	IL-12	R837	no lipid vs. 5 uM	SEM	3		1-way ANOVA, Dunnett	< 0.0001	****
		pparg -/-	15d-PGJ2	IL-12	R837	no lipid vs. 2.5 uM	SEM	3		1-way ANOVA, Dunnett	0.0005	***
		pparg +/+	15d-PGJ2	IL-12	R837	no lipid vs. 20 uM	SEM	3		1-way ANOVA, Dunnett	< 0.0001	****
		pparg +/+	15d-PGJ2	IL-12	R837	no lipid vs. 10 uM	SEM	3		1-way ANOVA, Dunnett	< 0.0001	****
		pparg +/+	15d-PGJ2	IL-12	R837	no lipid vs. 5 uM	SEM	3		1-way ANOVA, Dunnett	< 0.0001	****

		pparg +/+	15d-PGJ2	IL-12	R837	no lipid vs. 2.5 uM	SEM	3		1-way ANOVA, Dunnett	< 0.0001	****
		nrf2 -/-	15d-PGJ2	IL-12	R837	no lipid vs. 20 uM	SEM	3		1-way ANOVA, Dunnett	0.3625	ns
		nrf2 -/-	15d-PGJ2	IL-12	R837	no lipid vs. 10 uM	SEM	3		1-way ANOVA, Dunnett	0.7379	ns
		nrf2 -/-	15d-PGJ2	IL-12	R837	no lipid vs. 5 uM	SEM	3		1-way ANOVA, Dunnett	0.8127	ns
		nrf2 -/-	15d-PGJ2	IL-12	R837	no lipid vs. 2.5 uM	SEM	3		1-way ANOVA, Dunnett	0.2942	ns
		nrf2 +/+	15d-PGJ2	IL-12	R837	no lipid vs. 20 uM	SEM	3		1-way ANOVA, Dunnett	< 0.0001	****
		nrf2 +/+	15d-PGJ2	IL-12	R837	no lipid vs. 10 uM	SEM	3		1-way ANOVA, Dunnett	< 0.0001	****
		nrf2 +/+	15d-PGJ2	IL-12	R837	no lipid vs. 5 uM	SEM	3		1-way ANOVA, Dunnett	< 0.0001	****
		nrf2 +/+	15d-PGJ2	IL-12	R837	no lipid vs. 2.5 uM	SEM	3		1-way ANOVA, Dunnett	< 0.0001	****
4	b	wt	EC	nqo1	R837	0h vs. 2h	SD	2	n = 2	1-way ANOVA, Dunnett	0.0019	**
		wt	EC	nqo1	R837	0h vs. 4h	SD	2		1-way ANOVA, Dunnett	< 0.0001	****
		wt	EC	nqo1	R837	0h vs. 6h	SD	2		1-way ANOVA, Dunnett	< 0.0001	****
		wt	EC	nqo1	R837	0h vs. 8h	SD	2		1-way ANOVA, Dunnett	< 0.0001	****
		nrf2 -/-	EC	nqo1	R837	0h vs. 2h	SD	2		1-way ANOVA, Dunnett	0.5881	ns
		nrf2 -/-	EC	nqo1	R837	0h vs. 4h	SD	2		1-way ANOVA, Dunnett	0.9634	ns
		nrf2 -/-	EC	nqo1	R837	0h vs. 6h	SD	2		1-way ANOVA, Dunnett	0.6533	ns
		nrf2 -/-	EC	nqo1	R837	0h vs. 8h	SD	2		1-way ANOVA, Dunnett	0.629	ns
		wt	EC	hmox-1	R837	0h vs. 2h	SD	2		1-way ANOVA, Dunnett	0.0202	*
		wt	EC	hmox-1	R837	0h vs. 4h	SD	2		1-way ANOVA, Dunnett	0.0004	***
		wt	EC	hmox-1	R837	0h vs. 6h	SD	2		1-way ANOVA, Dunnett	0.0032	**
		wt	EC	hmox-1	R837	0h vs. 8h	SD	2		1-way ANOVA, Dunnett	0.4701	ns
		nrf2 -/-	EC	hmox-1	R837	0h vs. 2h	SD	2		1-way ANOVA, Dunnett	0.6491	ns
		nrf2 -/-	EC	hmox-1	R837	0h vs. 4h	SD	2		1-way ANOVA, Dunnett	0.2282	ns
		nrf2 -/-	EC	hmox-1	R837	0h vs. 6h	SD	2		1-way ANOVA, Dunnett	0.7083	ns
		nrf2 -/-	EC	hmox-1	R837	0h vs. 8h	SD	2		1-way ANOVA, Dunnett	> 0.9999	ns
4	c	wt	EC	gclc	LPS	BisRed vs. 15d-PGJ2	SD	2	n = 2	1-way ANOVA, Dunnett	0.1836	ns
		wt	15d-PGJ2	gclc	LPS	BisRed vs. EC	SD	2		1-way ANOVA, Dunnett	0.0039	**
		nrf2 -/-	EC	gclc	LPS	BisRed vs. 15d-PGJ2	SD	2		1-way ANOVA, Dunnett	0.3133	ns
		nrf2 -/-	15d-PGJ2	gclc	LPS	BisRed vs. EC	SD	2		1-way ANOVA, Dunnett	0.5486	ns
		pparg -/-	EC	gclc	LPS	BisRed vs. 15d-PGJ2	SD	2		1-way ANOVA, Dunnett	0.0042	**
		pparg -/-	15d-PGJ2	gclc	LPS	BisRed vs. EC	SD	2		1-way ANOVA, Dunnett	0.0002	***
		wt	EC	gsta3	LPS	BisRed vs. 15d-PGJ2	SD	2		1-way ANOVA, Dunnett	0.0372	*
		wt	15d-PGJ2	gsta3	LPS	BisRed vs. EC	SD	2		1-way ANOVA, Dunnett	0.0156	*
		nrf2 -/-	EC	gsta3	LPS	BisRed vs. 15d-PGJ2	SD	2		1-way ANOVA, Dunnett	0.253	ns
		nrf2 -/-	15d-PGJ2	gsta3	LPS	BisRed vs. EC	SD	2		1-way ANOVA, Dunnett	0.1433	ns
		pparg -/-	EC	gsta3	LPS	BisRed vs. 15d-PGJ2	SD	2		1-way ANOVA, Dunnett	0.0417	*
		pparg -/-	15d-PGJ2	gsta3	LPS	BisRed vs. EC	SD	2		1-way ANOVA, Dunnett	0.0078	**
4	d	wt	EC	il6	LPS	R837 + solvent vs. R837 + EC	SD	3,3	n = 2	1-way ANOVA, Dunnett	< 0.0001	****
		wt	15d-PGJ2	il6	LPS	R837 + solvent vs. R837 + 15d-PGJ2	SD	3,3		1-way ANOVA, Dunnett	< 0.0001	****
		nrf2 -/-	EC	il6	LPS	R837 + solvent vs. R837 + EC	SD	3,3		1-way ANOVA, Dunnett	0.9639	ns
		nrf2 -/-	15d-PGJ2	il6	LPS	R837 + solvent vs. R837 + 15d-PGJ2	SD	3,3		1-way ANOVA, Dunnett	0.8219	ns
		pparg -/-	EC	il6	LPS	R837 + solvent vs. R837 + EC	SD	2,3		1-way ANOVA, Dunnett	0.0003	***
		pparg -/-	15d-PGJ2	il6	LPS	R837 + solvent vs. R837 + 15d-PGJ2	SD	2,2		1-way ANOVA, Dunnett	0.0018	**
		wt	EC	il12	LPS	R837 + solvent vs. R837 + EC	SD	3,3		1-way ANOVA, Dunnett	< 0.0001	****
		wt	15d-PGJ2	il12	LPS	R837 + solvent vs. R837 + 15d-PGJ2	SD	3,3		1-way ANOVA, Dunnett	< 0.0001	****
		nrf2 -/-	EC	il12	LPS	R837 + solvent vs. R837 + EC	SD	3,3		1-way ANOVA, Dunnett	0.9999	ns
		nrf2 -/-	15d-PGJ2	il12	LPS	R837 + solvent vs. R837 + 15d-PGJ2	SD	3,3		1-way ANOVA, Dunnett	0.7874	ns
		pparg -/-	EC	il12	LPS	R837 + solvent vs. R837 + EC	SD	2,3		1-way ANOVA, Dunnett	< 0.0001	****

		pparg -/-	15d-PGJ2	il12	LPS	R837 + solvent vs. R837 + 15d-PGJ2	SD	2,2		1-way ANOVA, Dunnett	< 0.0001	****
4	e	wt	15d-PGJ2	ccl2	R837	R837 vs. R837 + PGJ2	SEM	2,3	n = 3	1-way ANOVA, Dunnett	< 0.0001	****
		wt	EC	ccl2	R837	R837 vs. R837 + EC	SEM	2,3		1-way ANOVA, Dunnett	< 0.0001	****
		wt	15d-PGJ2	ccl3	R837	R837 vs. R837 + PGJ2	SEM	2,3		1-way ANOVA, Dunnett	< 0.0001	****
		wt	EC	ccl3	R837	R837 vs. R837 + EC	SEM	2,3		1-way ANOVA, Dunnett	0.0003	***
		wt	15d-PGJ2	ccl4	R837	R837 vs. R837 + PGJ2	SEM	2,3		1-way ANOVA, Dunnett	0.0005	***
		wt	EC	ccl4	R837	R837 vs. R837 + EC	SEM	2,3		1-way ANOVA, Dunnett	0.0008	***
		wt	15d-PGJ2	ccl5	R837	R837 vs. R837 + PGJ2	SEM	2,3		1-way ANOVA, Dunnett	0.0142	*
		wt	EC	ccl5	R837	R837 vs. R837 + EC	SEM	2,3		1-way ANOVA, Dunnett	0.0531	ns
		wt	15d-PGJ2	cxcl10	R837	R837 vs. R837 + PGJ2	SEM	2,3		1-way ANOVA, Dunnett	0.0003	***
		wt	EC	cxcl10	R837	R837 vs. R837 + EC	SEM	2,3		1-way ANOVA, Dunnett	0.0005	***
		wt	15d-PGJ2	cxcl1	R837	R837 vs. R837 + PGJ2	SEM	2,3		1-way ANOVA, Dunnett	0.0163	*
		wt	EC	cxcl1	R837	R837 vs. R837 + EC	SEM	2,3		1-way ANOVA, Dunnett	0.0523	ns
		wt	15d-PGJ2	cxcl2	R837	R837 vs. R837 + PGJ2	SEM	2,3		1-way ANOVA, Dunnett	0.0069	**
		wt	EC	cxcl2	R837	R837 vs. R837 + EC	SEM	2,3		1-way ANOVA, Dunnett	0.0042	**
5	b	wt	EC	adherent cell no	LPS/GalN	EC vs DPPC	SEM	92'109	n = 2	unpaired, two-tailed t-test	< 0.0001	****
5	c	wt	EC	adherent cell no	LPS/GalN	EC vs DPPC	SEM	10,14	n = 2	unpaired, two-tailed t-test	< 0.0001	****
5	d	wt	EC	lung tot no.	LPS/GalN	DPPC vs EC	SEM	7,6	n = 2	unpaired, two-tailed t-test	0.0083	**
		wt	EC	lung neutrophil no	LPS/GalN	DPPC vs EC	SEM	7,6		unpaired, two-tailed t-test	0.0422	*
5	f	wt	EC	IL-6	R837	DPPC vs EC	SEM	3	n = 2	unpaired, two-tailed t-test	< 0.0001	****
5	g	wt	EC	IL-12	R837	DPPC vs EC	SEM	3	n = 2	unpaired, two-tailed t-test	< 0.0001	****
7	b	wt	EC	IL-6	R837	Solv vs. EC	SEM	2	n = 3	1-way ANOVA, Dunnett	0.0082	**
		wt	cEC	IL-6	R837	Solv vs. cEC	SEM	2	n = 3	1-way ANOVA, Dunnett	0.0004	***
		wt	EC	IL-12	R837	Solv vs. EC	SEM	2		1-way ANOVA, Dunnett	0.0023	**
		wt	cEC	IL-12	R837	Solv vs. cEC	SEM	2		1-way ANOVA, Dunnett	0.0006	***
7	c	wt	cEC	hmox-1	R837	DPPC vs cEC	SD	3	n = 3	unpaired, two-tailed t-test	0.0038	**
		wt	cEC	nqo1	R837	DPPC vs cEC	SD	3		unpaired, two-tailed t-test	0.0204	*
7	f	wt	cEC	ccl2	LPS	0 nM vs 500 nM	SD	2	n = 3	unpaired, two-tailed t-test	0.0043	**
		wt	cEC	ccl3	LPS	0 nM vs 500 nM	SD	2		unpaired, two-tailed t-test	0.0141	*
		wt	cEC	ccl4	LPS	0 nM vs 500 nM	SD	2		unpaired, two-tailed t-test	0.0273	*
		wt	cEC	ccl5	LPS	0 nM vs 500 nM	SD	2		unpaired, two-tailed t-test	0.0083	**
7	g	wt	EC	lung cell no	LPS/GalN	BisRed vs. EC	SD	6	n = 2	1-way ANOVA, Dunnett	< 0.0001	****
		wt	cEC	lung cell no	LPS/GalN	BisRed vs. cEC	SD	6		1-way ANOVA, Dunnett	0.0017	**
		wt	EC	lung neutrophil no	LPS/GalN	BisRed vs. EC	SD	6		1-way ANOVA, Dunnett	< 0.0001	****
		wt	cEC	ung neutrophil no	LPS/GalN	BisRed vs. cEC	SD	6		1-way ANOVA, Dunnett	< 0.0001	****
		wt	EC	BAL cell no	LPS/GalN	BisRed vs. EC	SD	6		1-way ANOVA, Dunnett	< 0.0001	****
		wt	cEC	BAL cell no	LPS/GalN	BisRed vs. cEC	SD	6		1-way ANOVA, Dunnett	< 0.0001	****
		wt	EC	BAL neutrophils	LPS/GalN	BisRed vs. EC	SD	6		1-way ANOVA, Dunnett	< 0.0001	****
		wt	cEC	BAL neutrophils	LPS/GalN	BisRed vs. cEC	SD	6		1-way ANOVA, Dunnett	< 0.0001	****
7	h	wt	15d-PGJ2	IFNg	gp61	DPPC vs. 15d-PGJ2	SD	4,2	n = 2	1-way ANOVA, Dunnett	0.9524	ns
		wt	PECPC	IFNg	gp61	DPPC vs. PECPC	SD	4,2		1-way ANOVA, Dunnett	0.0067	**
		wt	EC	IFNg	gp61	DPPC vs. EC	SD	4,2		1-way ANOVA, Dunnett	0.0005	***
		wt	cEC	IFNg	gp61	DPPC vs. cEC	SD	4,2		1-way ANOVA, Dunnett	< 0.0001	****
		wt	15d-PGJ2	IL-4	gp61	DPPC vs. 15d-PGJ2	SD	4,2		1-way ANOVA, Dunnett	0.8152	ns
		wt	PECPC	IL-4	gp61	DPPC vs. PECPC	SD	4,2		1-way ANOVA, Dunnett	0.9998	ns
		wt	EC	IL-4	gp61	DPPC vs. EC	SD	4,2		1-way ANOVA, Dunnett	0.0144	*
		wt	cEC	IL-4	gp61	DPPC vs. cEC	SD	4,2		1-way ANOVA, Dunnett	< 0.0001	****
Suppl. 3	a	wt / nrf2-/-		IL-6	CpG	wt vs nrf2-/-	SD	3		unpaired, two-tailed t-test	0.0059	**

		wt / nrf2-/-		IL-12	CpG	wt vs nrf2-/-	SD	3		unpaired, two-tailed t-test	< 0.0001	****
		wt / nrf2-/-		IL-6	R837	wt vs nrf2-/-	SD	3		unpaired, two-tailed t-test	0.0144	*
		wt / nrf2-/-		IL-12	R837	wt vs nrf2-/-	SD	3		unpaired, two-tailed t-test	< 0.0001	****
Suppl. 4		BAL wt		hmox-1	LPS	DPPC vs. cEC	SEM	4		1-way ANOVA, Dunnett	< 0.0001	****
		BAL wt		hmox-1	LPS	DPPC vs. EC	SEM	4		1-way ANOVA, Dunnett	0.0001	***
		BAL wt		hmox-1	LPS	DPPC vs. PECPC	SEM	4		1-way ANOVA, Dunnett	0.0185	*
		BAL wt		nqo1	LPS	DPPC vs. cEC	SEM	4		1-way ANOVA, Dunnett	< 0.0001	****
		BAL wt		nqo1	LPS	DPPC vs. EC	SEM	4		1-way ANOVA, Dunnett	0.0007	***
		BAL wt		nqo1	LPS	DPPC vs. PECPC	SEM	4		1-way ANOVA, Dunnett	0.1701	ns
		Lung wt		hmox-1	LPS	DPPC vs. cEC	SEM	4		1-way ANOVA, Dunnett	0.0002	***
		Lung wt		hmox-1	LPS	DPPC vs. EC	SEM	4		1-way ANOVA, Dunnett	0.0124	*
		Lung wt		hmox-1	LPS	DPPC vs. PECPC	SEM	4		1-way ANOVA, Dunnett	0.5654	ns
		Lung wt		nqo1	LPS	DPPC vs. cEC	SEM	4		1-way ANOVA, Dunnett	0.0004	***
		Lung wt		nqo1	LPS	DPPC vs. EC	SEM	4		1-way ANOVA, Dunnett	0.0015	**
		Lung wt		nqo1	LPS	DPPC vs. PECPC	SEM	4		1-way ANOVA, Dunnett	0.0151	*
Suppl 5	a	wt	Lipoxin B4	IL-6	R837	0 μM vs. 10 μM	SD	3		1-way ANOVA, Dunnett	0.3648	ns
		wt	Lipoxin B4	IL-6	R837	0 μM vs. 5 μM	SD	3		1-way ANOVA, Dunnett	0.9993	ns
		wt	Lipoxin B4	IL-6	R837	0 μM vs. 2.5 μM	SD	3		1-way ANOVA, Dunnett	0.9554	ns
		wt	Lipoxin B4	IL-6	R837	0 μM vs. 1.25 μM	SD	3		1-way ANOVA, Dunnett	0.9976	ns
		wt	15d-PGJ2	IL-6	R837	0 μM vs. 20 μM	SD	3		1-way ANOVA, Dunnett	< 0.0001	****
		wt	15d-PGJ2	IL-6	R837	0 μM vs. 10 μM	SD	3		1-way ANOVA, Dunnett	< 0.0001	****
		wt	15d-PGJ2	IL-6	R837	0 μM vs. 5 μM	SD	3		1-way ANOVA, Dunnett	< 0.0001	****
		wt	15d-PGJ2	IL-6	R837	0 μM vs. 2.5 μM	SD	3		1-way ANOVA, Dunnett	0.0018	**
		wt	15d-PGJ2	IL-6	R837	0 μM vs. 1.25 μM	SD	3		1-way ANOVA, Dunnett	0.9706	ns
		wt	EC	IL-6	R837	0 nM vs. 1.25 μM	SD	3		1-way ANOVA, Dunnett	< 0.0001	****
		wt	EC	IL-6	R837	0 nM vs. 625 nM	SD	3		1-way ANOVA, Dunnett	< 0.0001	****
		wt	EC	IL-6	R837	0 nM vs. 313 nM	SD	3		1-way ANOVA, Dunnett	0.0004	***
		wt	EC	IL-6	R837	0 nM vs. 156 nM	SD	3		1-way ANOVA, Dunnett	0.4883	ns
		wt	EC	IL-6	R837	0 nM vs. 78 nM	SD	3		1-way ANOVA, Dunnett	0.2549	ns
		wt	EC	IL-6	R837	0 nM vs. 39 nM	SD	3		1-way ANOVA, Dunnett	0.4558	ns
		wt	BisRed	IL-6	R837	0 μM vs. 40 μM	SD	3		1-way ANOVA, Dunnett	0.0268	*
		wt	BisRed	IL-6	R837	0 μM vs. 20 μM	SD	3		1-way ANOVA, Dunnett	0.0641	ns
		wt	BisRed	IL-6	R837	0 μM vs. 10 μM	SD	3		1-way ANOVA, Dunnett	0.1431	ns
		wt	Resolvin D2	IL-6	R837	0 μM vs. 10 μM	SD	3		1-way ANOVA, Dunnett	0.0504	ns
		wt	Resolvin D2	IL-6	R837	0 μM vs. 5 μM	SD	3		1-way ANOVA, Dunnett	> 0.9999	ns
		wt	Resolvin D2	IL-6	R837	0 μM vs. 2.5 μM	SD	3		1-way ANOVA, Dunnett	0.9998	ns
		wt	Resolvin D2	IL-6	R837	0 μM vs. 1.25 μM	SD	3		1-way ANOVA, Dunnett	0.958	ns
		wt	PGI2	IL-6	R837	0 μM vs. 20 μM	SD	3		1-way ANOVA, Dunnett	0.1782	ns
		wt	PGI2	IL-6	R837	0 μM vs. 10 μM	SD	3		1-way ANOVA, Dunnett	0.0822	ns
		wt	PGI2	IL-6	R837	0 μM vs. 5 μM	SD	3		1-way ANOVA, Dunnett	0.124	ns
		wt	PGI2	IL-6	R837	0 μM vs. 2.5 μM	SD	3		1-way ANOVA, Dunnett	0.5139	ns
		wt	PGI2	IL-6	R837	0 μM vs. 1.25 μM	SD	3		1-way ANOVA, Dunnett	0.4043	ns
		wt	cEC	IL-6	R837	0 nM vs. 625 nM	SD	3		1-way ANOVA, Dunnett	< 0.0001	****
		wt	cEC	IL-6	R837	0 nM vs. 313 nM	SD	3		1-way ANOVA, Dunnett	< 0.0001	****
		wt	cEC	IL-6	R837	0 nM vs. 156 nM	SD	3		1-way ANOVA, Dunnett	0.0007	***
		wt	cEC	IL-6	R837	0 nM vs. 78 nM	SD	3		1-way ANOVA, Dunnett	0.0587	ns

		wt	cEC	IL-6	R837	0 nM vs. 39 nM	SD	3		1-way ANOVA, Dunnett	0.9064	ns
		wt	DPPC	IL-6	R837	0 µM vs. 40 µM	SD	3		1-way ANOVA, Dunnett	0.0261	*
		wt	DPPC	IL-6	R837	0 µM vs. 20 µM	SD	3		1-way ANOVA, Dunnett	0.0703	ns
		wt	DPPC	IL-6	R837	0 µM vs. 10 µM	SD	3		1-way ANOVA, Dunnett	0.4182	ns
Suppl 5	b	wt	Lipoxin B4	IL-12	R837	0 µM vs. 10 µM	SD	3		1-way ANOVA, Dunnett	0.068	ns
		wt	Lipoxin B4	IL-12	R837	0 µM vs. 5 µM	SD	3		1-way ANOVA, Dunnett	0.9758	ns
		wt	Lipoxin B4	IL-12	R837	0 µM vs. 2.5 µM	SD	3		1-way ANOVA, Dunnett	0.9139	ns
		wt	Lipoxin B4	IL-12	R837	0 µM vs. 1.25 µM	SD	3		1-way ANOVA, Dunnett	0.5986	ns
		wt	15d-PGJ2	IL-12	R837	0 µM vs. 20 µM	SD	3		1-way ANOVA, Dunnett	< 0.0001	****
		wt	15d-PGJ2	IL-12	R837	0 µM vs. 10 µM	SD	3		1-way ANOVA, Dunnett	< 0.0001	****
		wt	15d-PGJ2	IL-12	R837	0 µM vs. 5 µM	SD	3		1-way ANOVA, Dunnett	< 0.0001	****
		wt	15d-PGJ2	IL-12	R837	0 µM vs. 2.5 µM	SD	3		1-way ANOVA, Dunnett	< 0.0001	****
		wt	15d-PGJ2	IL-12	R837	0 µM vs. 1.25 µM	SD	3		1-way ANOVA, Dunnett	0.0003	***
		wt	EC	IL-12	R837	0 nM vs. 1.25 µM	SD	3		1-way ANOVA, Dunnett	< 0.0001	****
		wt	EC	IL-12	R837	0 nM vs. 625 nM	SD	3		1-way ANOVA, Dunnett	< 0.0001	****
		wt	EC	IL-12	R837	0 nM vs. 313 nM	SD	3		1-way ANOVA, Dunnett	< 0.0001	****
		wt	EC	IL-12	R837	0 nM vs. 156 nM	SD	3		1-way ANOVA, Dunnett	< 0.0001	****
		wt	EC	IL-12	R837	0 nM vs. 78 nM	SD	3		1-way ANOVA, Dunnett	0.0018	**
		wt	EC	IL-12	R837	0 nM vs. 39 nM	SD	3		1-way ANOVA, Dunnett	0.9769	ns
		wt	BisRed	IL-12	R837	0 µM vs. 40 µM	SD	3		1-way ANOVA, Dunnett	0.135	ns
		wt	BisRed	IL-12	R837	0 µM vs. 20 µM	SD	3		1-way ANOVA, Dunnett	0.0138	*
		wt	BisRed	IL-12	R837	0 µM vs. 10 µM	SD	3		1-way ANOVA, Dunnett	0.1048	ns
		wt	Resolvin D2	IL-12	R837	0 µM vs. 10 µM	SD	3		1-way ANOVA, Dunnett	< 0.0001	****
		wt	Resolvin D2	IL-12	R837	0 µM vs. 5 µM	SD	3		1-way ANOVA, Dunnett	0.0608	ns
		wt	Resolvin D2	IL-12	R837	0 µM vs. 2.5 µM	SD	3		1-way ANOVA, Dunnett	0.9168	ns
		wt	Resolvin D2	IL-12	R837	0 µM vs. 1.25 µM	SD	3		1-way ANOVA, Dunnett	0.9999	ns
		wt	PGI2	IL-12	R837	0 µM vs. 20 µM	SD	3		1-way ANOVA, Dunnett	0.6035	ns
		wt	PGI2	IL-12	R837	0 µM vs. 10 µM	SD	3		1-way ANOVA, Dunnett	0.5383	ns
		wt	PGI2	IL-12	R837	0 µM vs. 5 µM	SD	3		1-way ANOVA, Dunnett	0.4834	ns
		wt	PGI2	IL-12	R837	0 µM vs. 2.5 µM	SD	3		1-way ANOVA, Dunnett	> 0.9999	ns
		wt	PGI2	IL-12	R837	0 µM vs. 1.25 µM	SD	3		1-way ANOVA, Dunnett	0.9979	ns
		wt	cEC	IL-12	R837	0 nM vs. 625 nM	SD	3		1-way ANOVA, Dunnett	< 0.0001	****
		wt	cEC	IL-12	R837	0 nM vs. 313 nM	SD	3		1-way ANOVA, Dunnett	< 0.0001	****
		wt	cEC	IL-12	R837	0 nM vs. 156 nM	SD	3		1-way ANOVA, Dunnett	< 0.0001	****
		wt	cEC	IL-12	R837	0 nM vs. 78 nM	SD	3		1-way ANOVA, Dunnett	< 0.0001	****
		wt	cEC	IL-12	R837	0 nM vs. 39 nM	SD	3		1-way ANOVA, Dunnett	0.002	**
		wt	DPPC	IL-12	R837	0 µM vs. 40 µM	SD	3		1-way ANOVA, Dunnett	0.4789	ns
		wt	DPPC	IL-12	R837	0 µM vs. 20 µM	SD	3		1-way ANOVA, Dunnett	0.6268	ns
		wt	DPPC	IL-12	R837	0 µM vs. 10 µM	SD	3		1-way ANOVA, Dunnett	0.8151	ns
Suppl 6		wt	EC	Gclc	R837	BisRed vs. EC	SD	2		1-way ANOVA, Sidak	< 0.0001	****
		wt	cEC	Gclc	R837	BisRed vs. cEC	SD	2		1-way ANOVA, Sidak	< 0.0001	****
		wt	BisRed	Gclc	R837	BisRed vs. 15d-PGJ2	SD	2		1-way ANOVA, Sidak	< 0.0001	****
		wt	15d-PGJ2	Gclc	R837	BisRed vs. PGI2	SD	2		1-way ANOVA, Sidak	0.8687	ns
		wt	Lipoxin B4	Gclc	R837	BisRed vs. LB4	SD	2		1-way ANOVA, Sidak	> 0.9999	ns
		wt	Resolvin D2	Gclc	R837	BisRed vs. PD2	SD	2		1-way ANOVA, Sidak	> 0.9999	ns
		wt	solvent	Gclc	R837	BisRed vs. solvent	SD	2		1-way ANOVA, Sidak	> 0.9999	ns

		wt	EC	Gsta3	R837	BisRed vs. EC	SD	2		1-way ANOVA, Sidak	< 0.0001	****
		wt	cEC	Gsta3	R837	BisRed vs. cEC	SD	2		1-way ANOVA, Sidak	< 0.0001	****
		wt	BisRed	Gsta3	R837	BisRed vs. 15d-PGJ2	SD	2		1-way ANOVA, Sidak	0.0029	**
		wt	15d-PGJ2	Gsta3	R837	BisRed vs. PGI2	SD	2		1-way ANOVA, Sidak	0.5302	ns
		wt	Lipoxin B4	Gsta3	R837	BisRed vs. LB4	SD	2		1-way ANOVA, Sidak	0.9376	ns
		wt	Resolvin D2	Gsta3	R837	BisRed vs. PD2	SD	2		1-way ANOVA, Sidak	0.8764	ns
		wt	solvent	Gsta3	R837	BisRed vs. solvent	SD	2		1-way ANOVA, Sidak	0.9985	ns
		wt	EC	Gsta3	R837	BisRed vs. EC	SD	2		1-way ANOVA, Sidak	< 0.0001	****
		wt	cEC	Gsta3	R837	BisRed vs. cEC	SD	2		1-way ANOVA, Sidak	< 0.0001	****
		wt	BisRed	Gsta3	R837	BisRed vs. 15d-PGJ2	SD	2		1-way ANOVA, Sidak	< 0.0001	****
		wt	15d-PGJ2	Gsta3	R837	BisRed vs. PGI2	SD	2		1-way ANOVA, Sidak	0.8687	ns
		wt	Lipoxin B4	Gsta3	R837	BisRed vs. LB4	SD	2		1-way ANOVA, Sidak	> 0.9999	ns
		wt	Resolvin D2	Gsta3	R837	BisRed vs. PD2	SD	2		1-way ANOVA, Sidak	> 0.9999	ns
		wt	solvent	Gsta3	R837	BisRed vs. solvent	SD	2		1-way ANOVA, Sidak	> 0.9999	ns
		wt	EC	Il12b	R837	BisRed vs. EC	SEM	2		1-way ANOVA, Dunnett	0.0036	**
		wt	cEC	Il12b	R837	BisRed vs. cEC	SEM	2		1-way ANOVA, Dunnett	0.003	**
		wt	EC	Il12a	R837	BisRed vs. EC	SEM	2		1-way ANOVA, Dunnett	0.0376	*
		wt	cEC	Il12a	R837	BisRed vs. cEC	SEM	2		1-way ANOVA, Dunnett	0.0342	*
		wt	EC	Il23	R837	BisRed vs. EC	SEM	2		1-way ANOVA, Dunnett	0.076	ns
		wt	cEC	Il23	R837	BisRed vs. cEC	SEM	2		1-way ANOVA, Dunnett	0.0653	ns
		wt	EC	Il6	R837	BisRed vs. EC	SEM	2		1-way ANOVA, Dunnett	< 0.0001	****
		wt	cEC	Il6	R837	BisRed vs. cEC	SEM	2		1-way ANOVA, Dunnett	< 0.0001	****
		wt	EC	Tnf	R837	BisRed vs. EC	SEM	2		1-way ANOVA, Dunnett	0.0016	**
		wt	cEC	Tnf	R837	BisRed vs. cEC	SEM	2		1-way ANOVA, Dunnett	0.0004	***
		wt	EC	Il10	R837	BisRed vs. EC	SEM	2		1-way ANOVA, Dunnett	0.7574	ns
		wt	cEC	Il10	R837	BisRed vs. cEC	SEM	2		1-way ANOVA, Dunnett	0.4651	ns
		wt	EC	hmox-1	R837	BisRed vs. EC	SEM	2		1-way ANOVA, Dunnett	< 0.0001	****
		wt	cEC	hmox-1	R837	BisRed vs. cEC	SEM	2		1-way ANOVA, Dunnett	< 0.0001	****
		wt	EC	nqo1	R837	BisRed vs. EC	SEM	2		1-way ANOVA, Dunnett	0.0003	***
		wt	cEC	nqo1	R837	BisRed vs. cEC	SEM	2		1-way ANOVA, Dunnett	0.0011	**
		wt	EC	Gclc	R837	BisRed vs. EC	SEM	2		1-way ANOVA, Dunnett	0.0018	**
		wt	cEC	Gclc	R837	BisRed vs. cEC	SEM	2		1-way ANOVA, Dunnett	0.0019	**
Suppl 10	b	wt	EC	Gsta3	R837	BisRed vs. EC	SEM	2		1-way ANOVA, Dunnett	0.0002	***
		wt	cEC	Gsta3	R837	BisRed vs. cEC	SEM	2		1-way ANOVA, Dunnett	0.0004	***
		wt	cEC	Gsta3	R837	BisRed vs. cEC	SEM	3,4		1-way ANOVA, Dunnett	< 0.0001	****
		wt	EC	Gsta3	R837	BisRed vs. EC	SEM	3,4		1-way ANOVA, Dunnett	0.0019	**
		wt	PECPC	Gsta3	R837	BisRed vs. PECPC	SEM	3,4		1-way ANOVA, Dunnett	0.0134	*
		wt	DPPC	Gsta3	R837	BisRed vs. DPPC	SEM	3,4		1-way ANOVA, Dunnett	> 0.9999	ns
		wt	cEC	nqo1	R837	BisRed vs. cEC	SEM	3,4		1-way ANOVA, Dunnett	0.0031	**
		wt	EC	nqo1	R837	BisRed vs. EC	SEM	3,4		1-way ANOVA, Dunnett	0.0179	*
		wt	PECPC	nqo1	R837	BisRed vs. PECPC	SEM	3,4		1-way ANOVA, Dunnett	0.0574	ns
		wt	DPPC	nqo1	R837	BisRed vs. DPPC	SEM	3,4		1-way ANOVA, Dunnett	> 0.9999	ns
