# Supplementary material for: Phospholipid oxidation generates potent anti-inflammatory lipid mediators that mimic structurally related pro-resolving eicosanoids by activating Nrf2
Source: EMBO Mol Med. 2015 Mar 13;7(5):593–607. doi: 10.15252/emmm.201404702 (PMC4492819; doi:10.15252/emmm.201404702)
Supplement: Supplementary file 2 [file emmm0007-0593-sd2.pdf]

Phospholipid oxidation generates potent anti-inflammatory lipid mediators that mimic structurally related pro-resolving eicosanoids by activating Nrf2

Peter Bretscher, Julian Egger, Abdijapar Shamshiev, Martin Trötzmüller, Harald Köfeler, Erick M. Carreira, Manfred Kopf, and Stefan Freigang

Corresponding authors: Manfred Kopf and Stefan Freigang, ETH Zurich

Review timeline:

Submission date:	01 October 2014
Editorial Decision:	28 October 2015
Revision received:	26 December 2014
Editorial Decision:	22 January 2015
Revision received:	15 February 2015
Accepted:	16 February 2015

Transaction Report:

(Note: With the exception of the correction of typographical or spelling errors that could be a source of ambiguity, letters and reports are not edited. The original formatting of letters and referee reports may not be reflected in this compilation.)

Editor: Céline Carret

1st Editorial Decision 28 October 2015

Thank you for the submission of your manuscript to EMBO Molecular Medicine. We have now heard back from the three referees whom we asked to evaluate your manuscript. Although the referees find the study to be of potential interest, they also raise a number of concerns that must be addressed in the next version of your study.

As you will see from the reports below, important issues regarding the conclusiveness and interpretation of the data have been raised that preclude publication at this stage. To further consider the manuscript, better statistical analyses are needed (ref.1) as well as convincing data reporting (please note our item number 8 below). Referee 2 highlights some technical issues that must be addressed to clarify the main message of the findings and thereby provide some mechanism. Finally referee 3 while more supportive, still requests additional details and clarifications, along with more appropriate referencing of previously reported findings.

Given the balance of these evaluations, we feel that we can consider a revision of your manuscript if you address the issues that have been raised to satisfy the referees. Please note that it is EMBO Molecular Medicine policy to allow only a single round of revision and that, as acceptance or rejection of the manuscript will depend on another round of review, your responses should be as complete as possible.

EMBO Molecular Medicine has a "scooping protection" policy, whereby similar findings that are published by others during review or revision are not a criterion for rejection. Should you decide to submit a revised version, I do ask that you get in touch after three months if you have not completed it, to update us on the status.

Please also contact us as soon as possible if similar work is published elsewhere. If other work is published we may not be able to extend the revision period beyond three months.

I look forward to receiving your revised manuscript.

***** Reviewer's comments *****

Referee #1 (Comments on Novelty/Model System):

Model of ALI is fine. The issue relies with the lack of proper negative (and positive? PGJ2 or LXA4) control.

Referee #1 (Remarks):

Bretscher et al have prepared a manuscript that illustrates a very interesting study where the Authors have employed multiple technologies to identify novel species of oxidised phospholipids with anti-inflammatory properties. A link with activation of a specific pathway leading to Nrf2 engagement with its downstream genes, ultimately leading to inhibition of cytokine synthesis in response to distinct TLR agonists, is unveiled.

1. I am very uneasy with the lack of statistical information. Methods do not tell us which stats was applied: this is not surprising because bar one figure, there is no statistical analysis in any figure!

Therefore, what is the N number of experiments tested? What is the number of replicates in each single experiment? Are data shown mean {plus minus} SEM or SD? What is the P value? In some instances it is quite evident that differences MIGHT be statistically significance (e.g. Fig 1E and 1F for LTA, or Fig 1I) but in many other instances this is unclear, e.g. Fig 1E for Poly-IC, LPS and R837 at 20 micrograms/ml, or indeed Figure 1H. We will all agree that if two groups are not statistically significance, when experiments have been repeated at least 3 times with an appropriate number of replicates, then discussion can take a certain angle or indeed, data may no merit discussion at all.

2. Throughout the study the Authors use the word 'potency' or 'potent effect' in a manner that is not pharmacologically accepted. In some cases, like Fig 3C or 7E, proper comparison for potency can be made since multiple concentrations were used, hence the ED50 can be derived with confidence. However in other cases, for instance on Page 9, is the effect of OxPAPC truly potent when indeed activity is seen at 1 microM?

3. The in vivo data are interesting and well presented, with some statistical analysis in the graphs (however, the reader does not learn how many mice were used, if the data are mean {plus minus} SEM or SD, which statistical assay was applied [likely student's T test]) but I have a concern on the control used. Is DPPC - a phospholipid - the best negative control for EC, which is a single unsaturated fatty acid derivative? Surely, an oxidised version of the epoxycyclopentenone would have been better rather than a glycerol base phospholipid.

One senses that the in-vivo experiments have been performed out of phase with other sections of the study (which is fine and it may happen). However, would not have been of fundamental importance establish if Nrf2 and downstream genes expression in lung tissue extracts was modulated by EC? I

would think this is required to close the circle and demonstrate causality versus casualty.

4. Page 5. The first paragraph of the Results seems more appropriate within the Introduction, and the Authors may want to be more precise about the inflammatory diseases where oxidation products of PAPC are relevant.

5. Page 6, I am not sure there is any need for 'data not shown' when supplementary figures are allowed. The Authors should show these data and indeed indicate at what concentrations the distinct oxidised products (mixture and single products) become toxic for the cells.

6. Page 7. Am I right that arachidonate oxidation is the important event here, upon incubation with cupper sulphate? How the identified products like cEC and EC fare when assessed against other AA-derived autacoids like prostacyclin and lipoxin A4 and B4 with respect to potency? The Authors may want to acknowledge the existence of other anti-inflammatory products derived from oxidation of AA.

7. Page 10, Line 9. I would remove 'completely' as 'abrogated' already indicates 100% inhibition.

8. Page 10, Line 16. I would qualify the 'comparably' and indicate if it is comparable potency (i.e. related to the active concentrations) or comparable efficacy (i.e. related to the degree of gene induction).

The same detail is required on Page 13 (Line 10) where cEC is compared to EC (Fig 7H). I think this is efficacy, as single concentrations were used.

9. Page 15, Line 21. 'triggers'.

10. Page 16, End of first paragraph. The existence of a 'tonic role' exerted by Nrf2 is of great interest and a major biological contribution of the study. What is the effect of PECPC if given in vivo to the mice undergoing ALI? One could predict formation of EC and activity through Nrf2.

11. Figure Legends lack fundamental information (aside the statistical analyses and details mentioned above). As an example, Fig 3D and 3F, what concentration of the indicated lipids was used. The legend tells us about the TLR agonists. Please pay close attention to this and similar information throughout the Legends to the Figures.

Fig 4C,D: how were the concentrations of 1 microM and 20 microM chosen for EC and PGJ2 respectively?

Figure 5B,C: should not one average the vessels analysed for each mouse and then average the mice used for each experimental group? Maybe a single graph suffices here. If not, the Authors should explain why in this models the analyses are performed in this way.

Figure 7G: should not cEC be compared to EC and PGJ2 rather than DPPC which does not contain AA?

Referee #2 (Comments on Novelty/Model System):

This is an interesting study suitable for EMBO Mol Med. It provides insight into the nature of lipid molecules derived from OxPL that mediate anti-inflammatory activity, and has broader implications

for our current understanding of the mechanisms by which lipids regulate inflammation. The only major concern is potential duplication of the data with other manuscripts. There are also some technical issues that need to be addressed.

Referee #2 (Remarks):

It is well known that oxidized phospholipids (OxPL) are not just inert byproducts of lipid peroxidation but rather, many of them, are bioactive and can modulate inflammation. Yet, it has been an area of debate whether OxPL exhibit pro-inflammatory or anti-inflammatory effects. To address this issue, Bretscher et al. investigated the effects of a particular class of differentially oxidized phospholipids (OxPL) generated during oxidative modification of the phospholipid precursor PAPC. They found that among the spectrum of lipid species generated, PECPC and PEIPC (and their active components EC and EI) were the main mediators of the anti-inflammatory effects of bulk OxPAPC preparations. PECPC and PEIPC exhibit structural similarities with known anti-inflammatory and pro-resolving lipid mediators and act by activating the transcription factor Nrf2. Overall, this is an interesting study. It provides insight into the nature of lipid molecules derived from OxPL that mediate anti-inflammatory activity, and sheds light into the mechanisms by which lipids regulate inflammation. However, there are several points that need to be addressed.

1. A major concern is how much of this work has been published recently in another article of the same group (Egger et al. Angew Chem Int Ed Engl. 2013 May 10;52(20):5382-5). Also, how much of this work will be published in a new manuscript coming out (Egger et al, 2014). There are certainly data there showing the inhibitory effects of PECPC/PEIPC or related lipids in BMDC IL-6 and IL-12 production, yet this is not mentioned in this manuscript. The authors should be very clear what has been published before (with ref.) and what is new. They should ensure that there is no duplication of the same data.

2. In Fig.1 and other Figures later on (e.g. Fig.4, Fig.5), have the authors looked at the regulation of other prominent cytokines beyond IL-6 and IL-12? What about TNF, IL-10 or IL-23? Is the inhibitory effect of OxPAPC (or PECPCEC and PEIPC/EI) specific for IL-6 and IL-12?

3. If the effect is specific for IL-6 and IL-12 (and certain chemokines) but not other cytokines, how can that be explained in terms of Nrf2 activation? Are IL-6 and IL12 regulated by Nrf2 but other cytokines are not? Do they have Nrf2 binding sites?

4. How is Nrf2 activated in response to OxPAPC? How is Nrf2 interfering with TLR-induced pro-inflammatory gene expression? Is there a model the authors are envisioning?

5. In Fig.1b mRNA levels are expressed in relation to G6PDH as the housekeeping gene. However, this enzyme is responsible for maintaining the balance of NADP and NADPH in cells, and thus their oxidative state. Could the expression of this enzyme be regulated upon exposure to OxPL in culture? How do the data look like if other housekeeping genes are used?

6. In Fig.1, how efficient were the oxidation reactions from PAPC to OxPAPC? How were the resulting products quantified so they could be subsequently added into cultures at the indicated 20 and 40 g/mL concentrations? Also, could addition of these OxPL species cause any stress to the cells or alter the pH in culture that could in turn account (at least partially) for the observed phenotype?

7. Are the OxPAPC, PECPC or PEIPC preparation endotoxin-free?

8. Are PAPC or OxPAPC lipids soluble?

9. Are the concentrations of oxPLs or their components used for in vitro or in vivo experiments physiologically relevant? Can these concentrations be reached in real life? This should be discussed.

10. Data on the potential contribution of toxicity to the inhibitory effects of OxPAPC should be shown, possibly as Supplemental.

11. In Fiig.4A, it's not clear what the different colour bars represent. The labelling for each bar should be clear.

12. It is not clear what Figure 5A shows. These are sections from the airways? Is this from proximal (large) or distal airways? Where is the Diff-Quick positive staining?

13. For Fig. 5D-E, It is mentioned that EC reduces neutrophil numbers in the lungs of mice. Yet, in the FACS plot of Fig.5E, neutrophils increase, at least as a percentage. This is in contract to the 'reduction' claimed by the authors and needs to be clarified.

14. Information about the number of experiments, numbers of mice/experiment etc are missing.

15. Information about the source(s) for the ELISA assays used should be provided. Also, when the authors refer to IL-12, is that IL-12 p70? Is that what the ELISA kit is measuring?

Referee #3 (Remarks):

The Merits of this manuscript: A clean and thorough examination of bioactivity of various compounds within OxPAPC, and most of all, generation of synthetic compounds that share the inflammation inhibitory effect. Also, to show that Nrf2 expression in the myeloid cells is necessary for the inflammation inhibitory effect has been performed thoroughly and clean, and the claims are of high significance, as such lipid mediators are promising candidate molecules for anti-inflammatory drugs.

Concerns of the reviewer with this manuscript: The evidence on how and in which detail the described compounds within OxPAPC (especially the active ones) were identified is missing from the manuscript and not detailed in the cited reference. This is absolutely required for clarity and reproducibility, but can be moved to supplementary data. The authors, in this reviewer's concern overstate the novelty of certain aspects in this manuscript, and need to acknowledge Birukov, et al. Circ. Res 2004, where a protective effect of PECPC has been described, Bl¸ml, et al. Blood 2009, where inhibition of IL-12 by OxPAPC independent of TLR4 antagonism has been described, and Jyrkk‰nen et al, where the Nrf2 agonistic activity of OxPAPC was investigated in detail.

Minor, general remark "Cancer, abstract line 4":

Oxidized phospholipid species (most of them not unequivocally identified) agreeably have a causative role- or are correlated to- chronic inflammation and metabolic disorders, which in turn are generally regarded as promoting cancer formation.

There is however, to this reviewer's knowledge no direct evidence showing that OxPL species contribute to cancer formation.

There is some evidence on mutagenicity of reactive carbonyl compounds that can arise from phospholipid oxidation (e.g.: 4-HNE), but not of those compounds that remain esterified to the backbone. Enzymes that can metabolize OxPL are strongly expressed in neoplastic tissue (Rolfs, et al), but no functional or causative "contribution to cancer of OxPL species" has been proven.

Minor, Figure 1: How much OxPAPC was used?, please note

Minor, Results Text @ Figure 1 C Page 5 line 24 : "We concluded from this result that modification of PAPC by oxygen radicals...": Contribution of non-radical ROS cannot to be excluded in the oxidation protocol used, and PAPC can also be oxidized and gain HO-1 inducing activity through a singlet oxygen dependent mechanism(see Gruber F, JBC 07) thus refer to ROS rather than "oxygen radicals".

Minor, Results Text @ Figure 1 E, F Page 6 line 14: Metabolic activity not shown: Please do show these data (as supplement). OxPL at this concentration can initiate ER stress and an UPR (Oskolkova Blood 2008 and Afonyushkin ATVB 2010), that would lead to altered cyto/chemokine and chaperone protein synthesis and might confound the detected inhibitory effect. Thus, an assay ruling out UPR/ER stress would improve the manuscript, e.g. assessing ATF3/4 and HSPAIA expression in existing untreated and lipid treated samples. This concern is to be regarded as a mere suggestion, if the cells used in Figure 1 and 3 are from mouse (it's not indicated) and correspond to those used to generate Figure 4.

Minor "Results Text @ Figure 1: E,F Page 6 line 15: "...inactivation of a particular TLR ligand.." The ability of OxPAPC to diminish IL12 production in DC not only after TLR4 but also TLR2 stimulation has been demonstrated by Bl¸ml et al. (Blood 2009) and should be acknowledged, thus it is not novel that the inhibition of inflammatory responses is not confined to a single TLR ligand.

MAJOR "Resuts Text @ Figure 2 and 3": Mass spectrometric analysis: The identification of OxPL species has to be presented in detail, as the distinct anti-inflammatory properties of the species are a major point of this study (presentation as supplementary data is acceptable). Information on, e.g. which diagnostic fragments of OxPLs had been used to identify the components of OxPAPC detailed in the figures need to be disseminated in a way that the experiment is reproducible by someone skilled in the art. The authors should also mention the limitations of the method employed on unequivocally identifying, e.g. PEIPC, or its isobaric forms which differ in biological activity, if there were such limitations. Fauland, 2011 which is cited in the Mat&Met section does not yield this information, neither does the related document supplied.

Minor, Last paragraph of results section P 13 line 17: "novel class of OxPL": Birukov KG, et al (2004), have described the epoxycyclopentenone oxidation product of PAPC (m/z 810) and it's protective function in lung damage and inflammation, so unless the authors are describing a different compound (for which evidence is needed) these OxPL class should not be referred to as new.

1st Revision - authors' response 26 December 2014

Point-by-point reply to the concerns raised by the reviewers

Referee #1

Bretscher et al have prepared a manuscript that illustrates a very interesting study where the Authors have employed multiple technologies to identify novel species of oxidised phospholipids with anti-inflammatory properties. A link with activation of a specific pathway leading to Nrf2 engagement with its downstream genes, ultimately leading to inhibition of cytokine synthesis in response to distinct TLR agonists, is unveiled.

Authors We thank the reviewer for the positive evaluation of our manuscript and for the helpful comments, which allowed us to significantly improve the revised manuscript.

Model of ALI is fine. The issue relies with the lack of proper negative (and positive? PGJ2 or LXA4) control.

Authors:
_______To address this concern of the reviewer, we have repeated the in vivo ALI experiment using "BisRed", a synthetic EC variant lipid, as a negative control. BisRed lacks the two essential electrophilic sites required for the anti-inflammatory bioactivity of EC (Fig 6). In addition, we now show that BisRed also lacks the ability to trigger Nrf2 signaling (Supplementary Fig S6), which confirms Nrf2 as the critical mediator of the anti-inflammatory bioactivity of EC. Provided that BisRed represents a 'Nrf2-signaling-dead' variant of EC (Fig 6 and Supplementary Fig S6) that is structurally much more similar to EC than the DPPC used previously, this result also suggested BisRed as ideal negative control lipid for our in vivo studies. As is evident from the revised Fig 7, only lipids that activate Nrf2 in vivo (i.e. EC and cEC; Supplementary Fig S4) prevent the sepsis-associated pulmonary inflammation, whereas BisRed does not. While we fully agree with the reviewer that the novel negative control BisRed significantly improves the quality of this experiment, we believe that inclusion of a positive control would not. Such a positive control would be critical to validate the experimental setup and detection methods, if we were trying to prove the absence of a biological effect, but not to demonstrate a bioactivity that can be directly measured. In addition, the in vitro characterization of other arachidonic acid-derived lipid mediators indicated that the anti-inflammatory bioactivity of epoxycyclopentenone lipids is shared by autacoids in general, but appears to be restricted to epoxycyclopentenones (Supplementary Fig S5). Thus, owing to the helpful comment of the reviewer, our revised version of the manuscript now includes a much more precise characterization of the anti-inflammatory bioactivity of EC.

1. I am very uneasy with the lack of statistical information. Methods do not tell us which stats was applied: this is not surprising because bar one figure, there is no statistical analysis in any figure! Therefore, what is the N number of experiments tested? What is the number of replicates in each single experiment? Are data shown mean {plus minus} SEM or SD? What is the P value? In some instances it is quite evident that differences MIGHT be statistically significance (e.g. Fig 1E and 1F for LTA, or Fig 1I) but in many other instances this is unclear, e.g. Fig 1E for Poly-IC, LPS and R837 at 20 micrograms/ml, or indeed Figure 1H. We will all agree that if two groups are not statistically significance, when experiments have been repeated at least 3 times with an appropriate number of replicates, then discussion can take a certain angle or indeed, data may no merit discussion at all.

Authors:
We apologize for the thoughtlessness not to include relevant statistics. We now provide all required statistical information in the respective Figures and Figure legends. We would like to point out to the reviewer that this statistical analysis fully supports our original

interpretation of the data. We therefore sincerely hope to have removed any of the reviewer's concerns in this matter.

2. **Throughout the study the Authors use the word 'potency' or 'potent effect' in a manner that is not pharmacologically accepted. In some cases, like Fig 3C or 7E, proper comparison for potency can be made since multiple concentrations were used, hence the ED50 can be derived with confidence. However in other cases, for instance on Page 9, is the effect of OxPAPC truly potent when indeed activity is seen at 1 microM?**

Authors:

We fully agree with the reviewer that the potency of different compounds can only be assessed and compared by determining the respective dose-response relationship, as we have done in the experiments depicted in Fig 3C or Fig 7E. To address the reviewer's concern, we have now adjusted the text of the manuscript accordingly.

3. The in vivo data are interesting and well presented, with some statistical analysis in the graphs (however, the reader does not learn how many mice were used, if the data are mean {plus minus} SEM or SD, which statistical assay was applied [likely student's T test]) but I have a concern on the control used. Is DPPC - a phospholipid - the best negative control for EC, which is a single unsaturated fatty acid derivative? Surely, an oxidised version of the epoxycyclopentenone would have been better rather than a glycerol base phospholipid.

Authors:

In response to this and previous comments of the reviewer regarding the validity of the controls, we have now repeated the in vivo experiments using an improved negative control. Although we still believe that its resistance to oxidation makes DPPC a valid control when compared to other phospholipids, including native PAPC and its oxidized derivatives, such as PECPC, we fully agree that DPPC may not be ideal when compared to the fatty acid-epoxycyclopentenone EC. To address this issue, we repeated the key experiments of our study using the EC-variant "BisRed" as negative control (Supplementary Fig S5, S6, S7 and S10). BisRed is structurally very similar to EC (Fig 6B), yet lacks its anti-inflammatory activity due to the absence of two essential electrophilic sites (Fig 6A,B and Supplementary Fig S5 and S7). As a consequence, BisRed also lacks the ability to induce Nrf2-mediated transcription of target genes (revised Fig 7, Supplementary Fig S6, S7 and S10). As could be expected from these results, BisRed confers no protection in the LPS-induced lung injury model, whereas EC and cEC significantly reduce both the total and neutrophilic infiltration into the lungs of endotoxin-challenged animals (revised Fig 7). These data clearly demonstrate that it is the ability to activate Nrf2-signaling in vivo that makes EC and cEC such highly potent anti-inflammatory compounds.

3. One senses that the in-vivo experiments have been performed out of phase with other sections of the study (which is fine and it may happen). However, would not have been of fundamental importance establish if Nrf2 and downstream genes expression in lung tissue extracts was modulated by EC? I would think this is required to close the circle and demonstrate causality versus casualty.

Authors reply:

In fact, we have performed the experiment as suggested by the reviewer and examined the induction of Nrf2 target genes in the infiltrating cells upon injection of the epoxycyclopentenone-containing lipids PEPCP, EC and cEC (Supplementary Fig S4). The results show a titrated induction of NQO1 and HMOX1 as would be expected from the respective potencies of these lipids, which we had previously determined in vitro (Fig 3C). This finding is complemented by the fact that the EC variant BisRed, which lacks the ability to activate Nrf2-signaling while still being structurally very similar to EC (revised Fig 7, Supplementary Fig S6 and S7), does not protect against LPS-induced lung injury in vivo

(revised Fig 7). Altogether, these findings indicate that activation of Nrf2-signaling is the common denominator of anti-inflammatory OxPL species, such as PECPC and EC. However, given that our results identified EC as the bioactive component of PECPC, we focused our efforts on this prostanoid during our further analysis.

4. Page 5. The first paragraph of the Results seems more appropriate within the Introduction, and the Authors may want to be more precise about the inflammatory diseases where oxidation products of PAPC are relevant.

Authors:

The text has been changed according to the reviewer's suggestion in the revised manuscript.

5. **Page 6, I am not sure there is any need for 'data not shown' when supplementary figures are allowed. The Authors should show these data and indeed indicate at what concentrations the distinct oxidised products (mixture and single products) become toxic for the cells.**

Authors:

As requested by the reviewer, we have included the data on potential toxicity of the lipid compounds as a new Supplementary Fig S1 and S2 in the revised manuscript. In particular, we now show both the metabolic activity (using Alamar Blue method) and the viability (using the viability dye eFluor780) of cells at the endpoint of experiments (Supplementary Fig S1, S2). These data reveal that cells remained metabolically active and viable at the concentrations where strong bioactivities (i.e. suppression of cytokine and chemokine secretion) were observed. We are therefore confident that our interpretation of the data is valid. Still, we agree that inclusion of this data set in the supplement helps to remove any concern regarding a potential toxicity of these lipids.

6. Page 7. Am I right that arachidonate oxidation is the important event here, upon incubation with cupper sulphate? How the identified products like cEC and EC fare when assessed against other AA-derived autacoids like prostacyclin and lipoxin A4 and B4 with respect to potency? The Authors may want to acknowledge the existence of other anti-inflammatory products derived from oxidation of AA.

Authors:

Indeed, oxidation of the arachidonic acid (AA) present in PAPC is the central event leading to the generation of a large array of bioactive lipid mediators from arachidonic acid-containing phospholipids (e.g. see Fig 2), including the epoxycyclopentenone-containing OxPL species we have characterized in our study in detail in vitro and in vivo. Since the experiments described in Fig 2 were focused on identifying OxPL species still linked to the phospholipid backbone, we did not detect any of the AA-derived lipid mediators mentioned by the reviewer in this analysis. However, we have now followed the reviewer's suggestion and assessed the ability of other prominent lipid mediators for their anti-inflammatory effects when compared side-by-side to EC and cEC. As is evident from Supplementary Fig S5 of the revised MS, neither Prostacyclin, Lipoxin B4 nor the pro-resolving lipid mediator Resolvin D2 influenced the secretion of the pro-inflammatory cytokines IL-12 or IL-6 in myeloid cells. In addition, Prostacyclin, Resolvin D2 and Lipoxin B4 did not trigger Nrf2-signaling (Supplementary Fig S6). These results suggest that these lipids regulate inflammation via other pathways than the one characterized in our study. Moreover, they highlight the importance of the cyclopentenone motif (present in PECPC, EC, cEC and PGJ2) to trigger Nrf2-singaling and downstream anti-inflammatory effects. We have adapted the manuscript text to comment on this result and refer to the importance of other AA-derived lipid mediators.

7. Page 10, Line 9. I would remove 'completely' as 'abrogated' already indicates 100% inhibition.

Authors: The text has been changed according to the reviewer's suggestion.

8. Page 10, Line 16. I would qualify the 'comparably' and indicate if it is comparable potency (i.e. related to the active concentrations) or comparable efficacy (i.e. related to the degree of gene induction).

Authors: The text has been changed according to the reviewer's suggestion.

8. The same detail is required on Page 13 (Line 10) where cEC is compared to EC (Fig 7H). I think this is efficacy, as single concentrations were used.

Authors: The text has been adjusted according to the reviewer's comment.

9. **Page 15, Line 21. 'triggers'.**

Authors: This has been corrected in the revised manuscript.

10. **Page 16, End of first paragraph. The existence of a 'tonic role' exerted by Nrf2 is of great interest and a major biological contribution of the study. What is the effect of PECPC if given in vivo to the mice undergoing ALI? One could predict formation of EC and activity through Nrf2.**

Authors: Our present study characterizes the anti-inflammatory bioactivity of epoxycyclo-pentenone-containing OxPL and provides strong evidence that this bioactivity is mediated via the transcription factor Nrf2. Thus, it is essential to show that EC-containing lipids do activate Nrf2 in vivo to mediate their effects. To address this, we have therefore performed two sets of experiments. First, we examined the ability of PEPCP, EC and cEC to activate Nrf2-signaling in lungs in vivo (Supplementary Fig S4). This experiment proves that like EC and cEC also PECPC induces Nrf2-signaling in vivo, most likely due to the release of the epoxycyclo-pentenone. Second, we then repeated the analysis of sepsis-associated lung injury and compare the activity of the active component of PECPC, EC, and that of the EC-derived lactone, cEC, to that of the 'Nrf2-signaling-dead' EC variant, BisRed (revised Fig 7). This study unequivocally demonstrated that only lipids able to activate Nrf2 protect against LPS-induced lung injury, which emphasizes the central message of our manuscript.

11. Figure Legends lack fundamental information (aside the statistical analyses and details mentioned above). As an example, Fig 3D and 3F, what concentration of the indicated lipids was used. The legend tells us about the TLR agonists. Please pay close attention to this and similar information throughout the Legends to the Figures.

Authors: We thank the reviewer for making us aware of this. The Figure legends have been adjusted accordingly throughout the revised manuscript to include all required information.

11. Fig 4C,D: how were the concentrations of 1 microM and 20 microM chosen for EC and PGJ2 respectively?

Authors: These concentrations of EC and 15d-PGJ2 were chosen considering the different potencies of these lipids. The dose-response-curves of the respective lipids (determined in Fig 3C, Fig 6A, Fig 7E) clearly indicated that EC is much more potent than 15d-PGJ2, and we therefore used a higher concentration of 15d-PGJ2 to compensate for this difference. In the experiment shown in Fig 4C,D we examined whether both lipids activated Nrf2 and whether both lipids could negatively regulate IL-6 and IL-12 responses at the transcriptional level.

Therefore, we used each lipid at a concentration for which we had observed a comparable level of bioactivity (inhibition of cytokine secretion) in our earlier experiments.

11. Figure 5B,C: should not one average the vessels analysed for each mouse and then average the mice used for each experimental group? Maybe a single graph suffices here. If not, the Authors should explain why in this models the analyses are performed in this way.

Authors: We thank the reviewer for bringing this issue to our attention. In fact, we had performed the analysis of the data exactly as suggested by the reviewer. First, a defined number of vessels was evaluated for each animal, and this data is depicted in Fig 5B. Second, we calculated the average values per individual mouse and then plotted the mean values of individual mice in Fig 5C. We believe that this presentation of the data allows the reader to appreciate the overall distribution of the data in both groups (Fig 5B) as well as the degree of variability between individual animals in the groups (Fig 5C). In order to clarify this issue, we have adjusted the respective Figure legend in the revised manuscript.

11. Figure 7G: should not cEC be compared to EC and PGJ2 rather than DPPC which does not contain AA?

Authors: As we have already discussed in detail in response to previous comments of the reviewer, the revised Fig 7 now included an experiment that compares the in vivo bioactivity of EC and cEC to that of the control lipid BisRed. As stated above and shown in Supplementary Fig S6 and S7, BisRed does not activate Nrf2 to induce transcription of its target genes and therefore does not inhibit the inflammatory cytokine response of myeloid cells in vitro (Supplementary Fig S5 and S6). Accordingly, BisRed provides a better control lipid than DPPC when assessing the bioactivity of EC and cEC in vivo. We find that the lipids able to activate Nrf2 in vitro (Fig 4, Fig 6, Supplementary Fig S5 and S6) and in vivo (Supplementary Fig S4) also protect mice from LPS-induced lung injury. In contrast, BisRed does not activate Nrf2 (Supplementary Fig S6 and S7) and does not protect against LPS-induced neutrophil infiltration in vivo (revised Fig 7). Together, these findings further support the notion that the anti-inflammatory activity of EC-containing lipids is imparted by their ability to activate Nrf2-mediated transcription. Thus, our revised manuscript clearly demonstrates that the ability to activate Nrf2 in vivo is common to all EC-containing lipids, including PECPC, EC and cEC.

Referee #2

This is an interesting study suitable for EMBO Mol Med. It provides insight into the nature of lipid molecules derived from OxPL that mediate anti-inflammatory activity, and has broader implications for our current understanding of the mechanisms by which lipids regulate inflammation. The only major concern is potential duplication of the data with other manuscripts. There are also some technical issues that need to be addressed.

It is well known that oxidized phospholipids (OxPL) are not just inert byproducts of lipid peroxidation but rather, many of them, are bioactive and can modulate inflammation. Yet, it has been an area of debate whether OxPL exhibit pro-inflammatory or anti-inflammatory effects. To address this issue, Bretscher et al. investigated the effects of a particular class of differentially oxidized phospholipids (OxPL) generated during oxidative modification of the phospholipid precursor PAPC. They found that among the spectrum of lipid species generated, PECPC and PEIPC (and their active components EC and EI) were the main mediators of the anti-inflammatory effects of bulk OxPAPC preparations. PECPC and PEIPC exhibit structural similarities with known anti-inflammatory and pro-resolving lipid mediators and act by activating the transcription factor Nrf2. Overall, this is an interesting study. It provides insight into the nature of lipid molecules derived from OxPL that mediate anti-inflammatory activity, and sheds light into the mechanisms by which lipids regulate inflammation. However, there are several points that need to be addressed.

1. A major concern is how much of this work has been published recently in another article of the same group (Egger et al. Angew Chem Int Ed Engl. 2013 May 10;52(20):5382-5). Also, how much of this work will be published in a new manuscript coming out (Egger et al, 2014). There are certainly data there showing the inhibitory effects of PECPC/PEIPC or related lipids in BMDC IL-6 and IL-12 production, yet this is not mentioned in this manuscript. The authors should be very clear what has been published before (with ref.) and what is new. They should ensure that there is no duplication of the same data.

Authors:

We would like to stress that the previous publication by Egger et al (Angew. Chem. Int. Ed. 2013) describes the chemical synthesis of EC-containing OxPL and uses cytokine secretion only as an in vitro readout for activity. This publication has been cited appropriately in the original version of our current study. In addition, for the purpose of full disclosure, we had provided to the editors of EMBO Mol Med and reviewers of the present manuscript, a second study that had been submitted in parallel to our study by our collaborators (Egger *et al*, 2014, JACI), which focuses on the development and chemical synthesis of epoxycyclopentenone variants including cyclo-EC from a chemist's perspective. Importantly, our present manuscript describes the identification and the biological activity of EC-containing OxPL in vitro and in vivo, including the effects on pro-inflammatory cytokines and chemokines, gene expression, signaling and the resulting regulation of endotoxin-induced lung injury and deviation of adaptive T cell responses (a switch from Th1 to Th2 cell development). While both studies show the effects of epoxyisoprostanes on IL-6 and IL-12 secretion in vitro, the respective data sets have been generated in independent experiments and are supportive. .The focus of both studies is completely different (chemical synthesis versus biological roles of epoxyisoprostanes in vitro and in vivo). To respond to this comment, we cite the first study of Egger *et al*. also with respect to the effects of OxPL on cytokine secretion in addition to the citation referring to the chemical synthesis. We sincerely hope that this satisfyingly answers the reviewer's comment and removes any concerns regarding the data presented in our study.

2. In Fig.1 and other Figures later on (e.g. Fig.4, Fig.5), have the authors looked at the regulation of other prominent cytokines beyond IL-6 and IL-12? What about TNF, IL-10

or IL-23? Is the inhibitory effect of OxPAPC (or PECPCEC and PEIPC/EI) specific for IL-6 and IL-12?

Authors:

To address the reviewer's comment, we have examined the effects of EC, cEC and the control BisRed on the expression of various cytokines. Results indicate that in addition to the transcriptional regulation of IL-6, IL-12p40 and IL-12p35 as well as a range of chemokines (Fig 4), the anti-inflammatory bioactivity of EC also extends to the cytokines IL-23p19 and TNF-alpha, whereas IL-10 appears to be unaffected (Supplementary Fig S7). This result further illustrates the broad anti-inflammatory activity of epoxycyclopentenone-containing OxPLs.

3 If the effect is specific for IL-6 and IL-12 (and certain chemokines) but not other cytokines, how can that be explained in terms of Nrf2 activation? Are IL-6 and IL12 regulated by Nrf2 but other cytokines are not? Do they have Nrf2 binding sites?

Authors:

As we have already discussed in our response to the previous comment, the effect of EC/Nrf2-signaling is much broader than only inhibition of IL-6 and IL-12 secretion, and includes several important chemokines (Fig 4 and Fig 7) as well as IL-23 and TNFalpha (Supplementary Fig S7). The most likely explanation for this broad anti-inflammatory activity of EC-mediated Nrf2-signaling is a direct inhibition of NFkB activation. We completely agree with the reviewer that the mechanisms linking OxPL/Nrf2-signaling to the regulation of pro-inflammatory responses in myeloid cells definitely merit further investigation. However, we feel that a detailed characterization of this molecular pathway is beyond the scope of the current manuscript, and we will therefore pursue this topic as a separate project in the future.

4. How is Nrf2 activated in response to OxPAPC? How is Nrf2 interfering with TLR-induced pro-inflammatory gene expression? Is there a model the authors are envisioning?

Authors:

At this point we can only speculate on how OxPL activate Nrf2-signaling, with modification of the chaperone protein Keap-1 by these electrophilic lipids being a feasible scenario. As already discussed in response to the previous comment, the most likely explanation for the broad anti-inflammatory activity of EC-mediated Nrf2-signaling would be the negative regulation of NFκB activation or signaling. We completely agree with the reviewer that the responsible mechanisms through which OxPL modulate the pro-inflammatory response in myeloid cells definitely merit further investigation. However, a more detailed characterization of this molecular pathway is beyond the scope of the current manuscript, and we will therefore pursue this topic as a separate project in the future.

5. In Fig.1b mRNA levels are expressed in relation to G6PDH as the housekeeping gene. However, this enzyme is responsible for maintaining the balance of NADP and NADPH in cells, and thus their oxidative state. Could the expression of this enzyme be regulated upon exposure to OxPL in culture? How do the data look like if other housekeeping genes are used?

Authors:

We have obtained comparable data when normalizing the gene expression levels to different housekeeping genes, including TATA-box-binding protein (TBP), eukaryotic translation elongation factor 1alpha 1 (Eef1a1), and beta-actin (Actb). For reasons of consistency we chose to present all data in the revised manuscript relative to G6PDH, but include the expression levels of the above housekeeping genes relative to G6PDH as Supplementary Fig S10A. This data demonstrates that no significant differences with respect

to a particular housekeeping gene exist between groups. Thus, the results reported in the initial version of the manuscript remain valid and support our interpretation of the data in full.

6. In Fig.1, how efficient were the oxidation reactions from PAPC to OxPAPC? How were the resulting products quantified so they could be subsequently added into cultures at the indicated 20 and 40microg/mL concentrations? Also, could addition of these OxPL species cause any stress to the cells or alter the pH in culture that could in turn account (at least partially) for the observed phenotype?

<u>Authors:</u>

Fig 1 of our study characterizes the anti-inflammatory bioactivity of different OxPAPC preparations that had been generated by oxidizing PAPC in presence of $CuSO_4$ or $FeSO_4$ for 24 hours or by exposure of PAPC to air for 72 hours. The mass spec analysis of these OxPAPC preparations (Fig 2) revealed that such in vitro oxidation resulted in the oxidative modification of more that 99% percent of the PAPC precursor phospholipid. We therefore based our calculation of the resulting concentration OxPAPC on the amount of PAPC that was subjected to oxidation. We think that this approximation is valid, given the high efficiency of oxidation achieved with this method (Fig 2). Furthermore, when added directly to our in vitro assays, PAPC did not show any bioactivity, suggesting that even small amounts of residual PAPC present in our OxPAPC preparations would not influence results (Fig 1). In addition, the difference in concentration should be negligible, especially considering that these OxPAPC preparations contain a broad spectrum of different oxidized lipid species with distinct bioactivities. As we demonstrate in our manuscript for the case of PECPC and EC, a given biological effect will most likely be caused only by a fraction of the whole bulk OxPL mixture. Importantly, we would like to stress at this point that the large majority of experimental data shown in our manuscript, i.e. the assessment of potency, the characterization of the bioactivity in vitro and in vivo, the examination of the structure-activity-relationship, the identification of the Nrf2-signaling pathway, as well as the gene expression studies, was generated using synthetic compounds used at well-defined concentrations.

Regarding the second part of this comment – we are confident that lipid-induced cellular stress or effects on pH can be excluded as explanation for the observed bioactivity. In particular, exposure of myeloid cells to EC did not induce the expression of genes involved in the ER stress response, such as *Xbp1* and *Ddit3* (CHOP) (Supplementary Fig S10C).

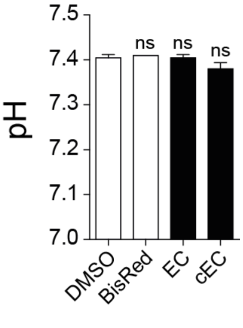

Moreover, no signification change in pH was observed upon addition of 20 microM EC or cEC (i.e. an 10-fold excess of the maximal concentration used to demonstrate the bioactivity for these lipids) to normal cell culture medium not containing HEPES (see above).

7. Are the OxPAPC, PECPC or PEIPC preparation endotoxin-free?

<u>Authors:</u>

Yes, the lipids used in our study were endotoxin-free. We used highly pure lipids obtained from commercial sources to generate our OxPAPC preparations. All buffers and media were prepared under endotoxin-free conditions using endotoxin-free material. In addition, PECPC and PEIPC were synthesized via established routes, including a final

evaluation of the purity of compounds by NMR analysis, which should have identified such potential contamination. More importantly, we here describe an anti-inflammatory activity of OxPL and actually use microbial products, such as endotoxin, in this process. Therefore, if these lipids contained any endotoxin-like activity, such contaminations should rather boost than inhibit the resulting cytokine responses in our experiments. Furthermore, if an endotoxin contamination of the synthetic lipid stocks were contributing to the observed effects, one would expect to also see these effects when using the modified EC-variants BisRed or MonoRedA (Fig 6), which were processed using the same methods and reagents as EC or cEC. However, these lipids completely lost their bioactivity, indicating that the presence of certain electrophilic sites within the epoxycyclopentenone, and not contaminating endotoxin was responsible for Nrf2 activation and subsequent inhibition of pro-inflammatory cytokine expression.

8. Are PAPC or OxPAPC lipids soluble?

Authors:

To examine the effect of lipid solubility on the observed biological effects, we have compared the bioactivity of different lipid formulations using the inhibition of TLR7-induced inflammatory cytokine production as a functional read-out. For this purpose, the effect of 15d-PGJ2 was analyzed as an isolated lipid, as a complex with fatty acid-free BSA, or incorporated into 100nm liposomes. However, neither solubilization of the lipid with BSA or in form of liposomes increased its bioactivity (Supplementary Fig S9). The lack of bioactivity of PAPC in our bioassay precluded us from performing a similar analysis for PAPC. Based on these results we concluded that the isolated lipid was readily available for cellular uptake and therefore used isolated compounds for all subsequent in vitro and in vivo experiments.

9. Are the concentrations of oxPLs or their components used for in vitro or in vivo experiments physiologically relevant? Can these concentrations be reached in real life? This should be discussed.

Authors:

We completely agree with the reviewer that the bioactivity of OxPL reported here by us is only physiologically relevant if the respective OxPL are also generated at these concentrations in vivo. In fact, we would have very much liked to quantify the different OxPL species, especially PECPC and EC, in samples isolated ex vivo from our animal studies. Unfortunately, our attempts have so far been hampered by technical constraints due the high background of unmodified phospholipids present in such lipid extracts. However, while we are not aware of studies quantifying PECPC or even EC in vivo, biologically relevant amounts (40-60 microg / g tissue) of the related OxPL PEIPC have been reported for atherosclerotic rabbit aortas in vivo (Bochkov *et al*, 2010). As suggested by the reviewer, we refer to this report in the revised manuscript.

10. **Data on the potential contribution of toxicity to the inhibitory effects of OxPAPC should be shown, possibly as Supplementary.**

Authors:

As requested by the reviewer, we have now included the data on potential toxicity of the lipid compounds as a new Supplementary Fig S1 and S2 in the revised manuscript. In particular, we have assessed both the metabolic activity (using Alamar Blue method) and the viability (using the viability dye eFluor780) of cells at the endpoint of experiments (Supplementary Fig S1 and S2). These data clearly show that at the concentrations where strong bioactivities (i.e. suppression of cytokine and chemokine secretion) are observed, cells remained metabolically active and viable. We are therefore confident that our interpretation of our data is valid. We agree that inclusion of this supplementary data will help to remove any concern regarding a potential toxicity of these lipids.

11- **In Fig.4A, it's not clear what the different colour bars represent. The labelling for each bar should be clear.**

Authors: This has been corrected in the revised Fig 4A.

12. It is not clear what Figure 5A shows. These are sections from the airways? Is this from proximal (large) or distal airways? Where is the Diff-Quick positive staining?

Authors:
The sections shown in Fig 5A shoe examples of the lung vasculature used to quantify leukocyte adhesion in mice treated with LPS and indicated lipids. These sections were stained with hematoxylin, not DiffQuik. We thank the reviewer for bringing this mistake to our attention and we have corrected this in the revised manuscript accordingly.

13. For Fig. 5D-E, It is mentioned that EC reduces neutrophil numbers in the lungs of mice. Yet, in the FACS plot of Fig.5E, neutrophils increase, at least as a percentage. This is in contract to the 'reduction' claimed by the authors and needs to be clarified.

Authors:
We thank the reviewer for bringing this potentially misleading figure to our attention and for giving us the opportunity to correct this in the revised manuscript. We have now replaced the respective dot plots in Fig 5 with a more representative data set. However, we would like to point out that also in the experiment used for the dot plots shown in the previous version of Fig 5, EC significantly reduced the absolute number of neutrophils infiltrating into the lungs of endotoxin-challenged mice. Thus, neither the result nor the interpretation of this result change.

14. Information about the number of experiments, numbers of mice/experiment etc are missing.

Authors:
We thank the reviewer for making us aware of this. The Figure legends have been adjusted accordingly throughout the revised manuscript to include all required information.

15. Information about the source(s) for the ELISA assays used should be provided. Also, when the authors refer to IL-12, is that IL-12 p70? Is that what the ELISA kit is measuring?

Authors:
We determined IL-12p70. However, we now provide data that EC/Nrf2-signaling similarly regulates the mRNA expression of IL-12p40, IL-12p35, and IL-23p19 - suggesting that it affects the secretion of both, IL-12 and IL-23.

Referee #3

Comment 1

***The Merits of this manuscript:* A clean and thorough examination of bioactivity of various compounds within OxPAPC, and most of all, generation of synthetic compounds that share the inflammation inhibitory effect. Also, to show that Nrf2 expression in the myeloid cells is necessary for the inflammation inhibitory effect has been performed thoroughly and clean, and the claims are of high significance, as such lipid mediators are promising candidate molecules for anti-inflammatory drugs.**

***Concerns of the reviewer with this manuscript:* The evidence on how and in which detail the described compounds within OxPAPC (especially the active ones) were identified is missing from the manuscript and not detailed in the cited reference. This is absolutely required for clarity and reproducibility, but can be moved to supplementary data.**

Answer to comment 1:

We thank the reviewer for the generally positive evaluation of our manuscript. With respect the reviewer's concerns regarding identification of the different OxPL species, we provide a detailed response to the related comment 9 of this reviewer and also include an additional Figure and explanation as supplementary data.

Comment 2

The authors, in this reviewer's concern overstate the novelty of certain aspects in this manuscript, and need to acknowledge Birukov, et al. Circ. Res 2004, where a protective effect of PECPC has been described, Blüml, et al. Blood 2009, where inhibition of IL-12 by OxPAPC independent of TLR4 antagonism has been described, and Jyrkkänen et al, where the Nrf2 agonistic activity of OxPAPC was investigated in detail.

Answer to comment 2:

We apologize that we missed to quote some manuscripts related to our story, which we make good for in the revised manuscript, as suggested by the reviewer. However, while we are aware of these studies and acknowledge their relevance to our manuscript, we would also like to point out to the reviewer that our study significantly extends these earlier reports and puts them into a completely new perspective. In particular, we have identified from the complex mixture of OxPAPC a distinct OxPL species that mediates strong anti-inflammatory effects in vitro as well as in vivo. Furthermore, we identified Nrf2 as the responsible transcription factor that mediates this bioactivity. In addition, our study employs novel synthetic variant lipids of this OxPL species to identify critical structural determinants of this anti-inflammatory bioactivity, as well as to demonstrate the importance of in vivo Nrf2-signaling for this anti-inflammatory effect. Finally, we describe the in vitro and in vivo activity of a novel EC-derived lactone that exhibits unprecedented anti-inflammatory activity.

Comment 3

Minor, general remark "Cancer, abstract line 4": Oxidized phospholipid species (most of them not unequivocally identified) agreeably have a causative role- or are correlated to- chronic inflammation and metabolic disorders, which in turn are generally regarded as promoting cancer formation. There is however, to this reviewer's knowledge no direct evidence showing that OxPL species contribute to cancer formation. There is some evidence on mutagenicity of reactive carbonyl compounds that can arise from phospholipid oxidation (e.g.: 4-HNE), but not of those compounds that remain esterified to the backbone. Enzymes that can metabolize OxPL are strongly expressed in neoplastic tissue (Rolfs, et al), but no functional or causative "contribution to cancer of OxPL species" has been proven.

Answer to comment 3:

We have adjusted the text of the revised manuscript accordingly.

Comment 4

Minor, Figure 1: How much OxPAPC was used?, please note

Answer to comment 4:

This information has been added to the legend describing Fig 1.

Comment 5

Minor, Results Text @ Figure 1 C Page 5 line 24: "We concluded from this result that modification of PAPC by oxygen radicals...": Contribution of non-radical ROS cannot to be excluded in the oxidation protocol used, and PAPC can also be oxidized and gain HO-1 inducing activity through a singlet oxygen dependent mechanism(see Gruber F, JBC 07) thus refer to ROS rather than "oxygen radicals".

Answer to comment 5:

We have adjusted the text of the revised manuscript accordingly.

Comment 6

Minor, Results Text @ Figure 1 E, F Page 6 line 14: Metabolic activity not shown: Please do show these data (as supplement).

Answer to comment 6:

As requested by the reviewer, we have now included the data on potential toxicity of the lipid compounds as a new Supplementary Fig S1 and S2 in the revised manuscript. In particular, we have assessed both the metabolic activity (using Alamar Blue method) and the viability (using the viability dye eFluor780) of cells at the endpoint of experiments (Supplementary Fig S1 and S2). These data clearly show that at the concentrations at which strong bioactivities (i.e. suppression of cytokine and chemokine secretion) are observed, cells remained metabolically active and viable. We are therefore confident that our interpretation of our data is valid. However, we agree that inclusion of this supplementary data helps to remove any concern regarding a potential toxicity of these lipids.

Comment 7

OxPL at this concentration can initiate ER stress and an UPR (Oskolkova Blood 2008 and Afonyushkin ATVB 2010) that would lead to altered cyto/chemokine and chaperone protein synthesis and might confound the detected inhibitory effect. Thus, an assay ruling out UPR/ER stress would improve the manuscript, e.g. assessing ATF3/4 and HSPAIA expression in existing untreated and lipid treated samples. This concern is to be regarded as a mere suggestion, if the cells used in Figure 1 and 3 are from mouse (it's not indicated) and correspond to those used to generate Figure 4.

Answer to comment 7:

All data presented in our manuscript were generated using primary mouse cells, and therefore the murine cells used in Fig 1 and Fig 3 correspond to those used to generate Fig 4. We understand that this fact may already answer the reviewer's comment. Nevertheless, to clear out any other concern of the reviewer, we addressed the potential induction of ER stress by the epoxycyclopentenone OxPL. However, no significant induction of *Xbp1* and *Ddit3* (CHOP) expression was observed at concentrations that induced the anti-inflammatory bioactivity (Supplementary Fig S10C). In addition, we now also include data on the metabolic activity and viability of cells exposed to the different lipids as Supplementary Fig S1 and S2. These results clearly demonstrate that cells remained viable and metabolically active at the endpoint of experiments. We conclude from these results that lipid-induced ER stress or

toxicity are an unlikely explanation for the strong anti-inflammatory effects observed in our experiments.

Comment 8

Minor "Results Text @ Figure 1: E,F Page 6 line 15: "...inactivation of a particular TLR ligand.." The ability of OxPAPC to diminish IL12 production in DC not only after TLR4 but also TLR2 stimulation has been demonstrated by Blüml et al. (Blood 2009) and should be acknowledged, thus it is not novel that the inhibition of inflammatory responses is not confined to a single TLR ligand.

Answer to comment 8:

The observation that OxPAPC and epoxycyclopentenone-containing OxPL inhibit the pro-inflammatory responses elicited by several TLR ligands is clearly not a central finding of our study. Nevertheless, in view of previous reports suggesting that one major effect of OxPAPC was inactivation of TLR ligands, and provided that we now also show this inhibitory activity for the TLR3, TLR7 and TLR9 in addition to TLR2 and TLR4, we felt that this aspect of the experiment should be stated. To address this concern of the reviewer, we refer to the study of Blüml et al. in the revised manuscript.

Comment 9

MAJOR "Results Text @ Figure 2 and 3": Mass spectrometric analysis: The identification of OxPL species has to be presented in detail, as the distinct anti-inflammatory properties of the species are a major point of this study (presentation as supplementary data is acceptable). Information on, e.g. which diagnostic fragments of OxPLs had been used to identify the components of OxPAPC detailed in the figures need to be disseminated in a way that the experiment is reproducible by someone skilled in the art. The authors should also mention the limitations of the method employed on unequivocally identifying, e.g. PEIPC, or its isobaric forms which differ in biological activity, if there were such limitations. Fauland, 2011 which is cited in the Mat&Met section does not yield this information, neither does the related document supplied.

Answer to comment 9:

We now included a supplementary data section (Supplementary Fig S8 and supplementary method) on mass spectrometric analysis of oxidized PAPC, which deals with this topic in depth. Nevertheless, we decided to leave the citation of Fauland *et al*. in the manuscript, since it covers all the experimental LC-MS settings applied to our oxidized PAPC samples like HPLC column, solvents and gradient or the detailed settings in the ion source, the ion cyclotron and the linear ion trap. The supplementary data section now covers the details about the process of identification of oxidized PAPC. Of course, full structural characterization including stereochemistry and exact location of double bonds will not be possible by low energy CID LC-MS/MS methods. To that end one would need high energy CID mass spectrometry like three- or four-sector instrumentation allowing for charge remote fragmentation. But since this kind of instrumentation can neither be efficiently coupled to chromatography nor shows the sensitivity required for analysis of OxPL it is no option of choice. These limitations of our method are now stated in the supplement. Nevertheless we use retention time, elemental composition of molecular ions by high resolution FT-ICR-MS and linear ion trap fragment spectra as lines of defense selectivity wise. Additionally we also factor in literature knowledge about possible oxidized products of PAPC. We exemplify this process at the example of P-HOdiA-PC (m/z 666.39762) because this shows the benefits of high resolution mass spectrometry, which would enable the separation of this compound from PAzPC (m/z 666.43401) if it were in the mixture. Now the inclined reader should be able to comprehend the process of identification and to subsequently repeat the LC-MS experiments.

Comment 10

Minor, Last paragraph of results section P 13 line 17: "novel class of OxPL": Birukov KG, et al (2004), have described the epoxycyclopentenone oxidation product of PAPC (m/z 810) and it's protective function in lung damage and inflammation, so unless the authors are describing a different compound (for which evidence is needed) these OxPL class should not be referred to as new.

<u>Answer to comment 10:</u>

We thank the reviewer for this comment and we are aware of the work of Birukov et al. However, our study demonstrates for the first time that the isolated fatty acid epoxycyclopentenone mediates the bioactivity described in our study and that it requires Nrf2. In addition, whereas Birukov et al. studied human pulmonary endothelial cells in vitro (which is not equivalent to lung damage), our manuscript characterizes the anti-inflammatory bioactivity of EC in an in vivo model of sepsis-associated lung injury. We also report the strong anti-inflammatory effects of EC and EC-containing lipids on primary dendritic cells, which are central for the induction of adaptive immunity. Moreover, our study characterizes the strong in vivo bioactivity of cyclo-EC, which clearly represents a novel, epoxycyclopentenone-derived OxPL species. In that sense it is novel. However, to avoid any misunderstanding in this regard, we have rephrased the respective text in the revised manuscript.

2nd Editorial Decision 22 January 2015

Thank you for the submission of your revised manuscript to EMBO Molecular Medicine. We have now received the enclosed report from the referee asked to re-assess it. As you will see this reviewer is now fully supportive and I am pleased to inform you that we will be able to accept your manuscript pending some final editorial amendments.

Please submit your revised manuscript within two weeks. I look forward to seeing a revised form of your manuscript as soon as possible.

***** Reviewer's comments *****

Referee #1 (Comments on Novelty/Model System):

I think the manuscript has greatly improved. The Authors have taken on board my comments, as well as those of the other reviewers. Definition of novel chemical structures and identification of active components in otherwise mixtures with biological efficacy is truly novel and may impact on medical developments.

Referee #1 (Remarks):

I am satisfied by the extra experiments the Authors have conducted. I am also satisfied with their reasoning and data interpretation.

I am glad new in-vivo experiments have been conducted and, in general, with the choice of an appropriate negative control. I agree with Authors that a positive control may be over-pushing it for the present study, though it would remain important for future development of cEC in order to gauge its pharmacodynamics.

I am delighted that statistical analyses have now been included, even if the overall message has not changed that much... clearly EMM could not data presentation without proper replicates and statistics.

© EMBO
